# Supplementary material for: Correction of a Factor VIII genomic inversion with designer-recombinases
Source: Nat Commun. 2022 Jan 20;13:422. doi: 10.1038/s41467-022-28080-7 (PMC8776779; doi:10.1038/s41467-022-28080-7)
Supplement: Supplementary file 1 — Supplementary Information [file 41467_2022_28080_MOESM1_ESM.pdf]

Supplementary information

Correction of a Factor VIII genomic inversion with designer-recombinases

Felix Lansing[1], Liliya Mukhametzyanova[1], Teresa Rojo-Romanos[1], Kentaro Iwasawa[2,3], Masaki Kimura[2], Maciej Paszkowski-Rogacz[1], Janet Karpinski[1], Tobias Grass[1,4], Jan Sonntag[1], Paul Martin Schneider[1], Ceren Günes[5], Jenna Hoersten[1], Lukas Theo Schmitt[1], Natalia Rodriguez-Muela[4], Ralf Knöfler[6], Takanori Takebe[2,3,7] & Frank Buchholz[1*]

[1] Medical Systems Biology, Medical Faculty, Technical University Dresden, 01307 Dresden, Germany

[2] Division of Gastroenterology, Hepatology and Nutrition, Division of Developmental Biology, Center for Stem Cell and Organoid Medicine (CuSTOM) Cincinnati Children's Hospital Medical Center, Cincinnati, OH, USA

[3] Department of Pediatrics, University of Cincinnati College of Medicine, Cincinnati, OH, USA

[4] German Center for Neurodegenerative Diseases, Helmholtz Association, 01307 Dresden, Germany

[5] Department of Cell and Developmental Biology, Max Planck Institute for Molecular Biomedicine, Röntgenstrasse 20, Münster 48149, Germany

[6] Department of Pediatric Hematology and Oncology, University Hospital Dresden, 01307 Dresden, Germany

[7] Institute of Research, Tokyo Medical and Dental University (TMDU), Tokyo, Japan

* Corresponding author: frank.buchholz@tu-dresden.de

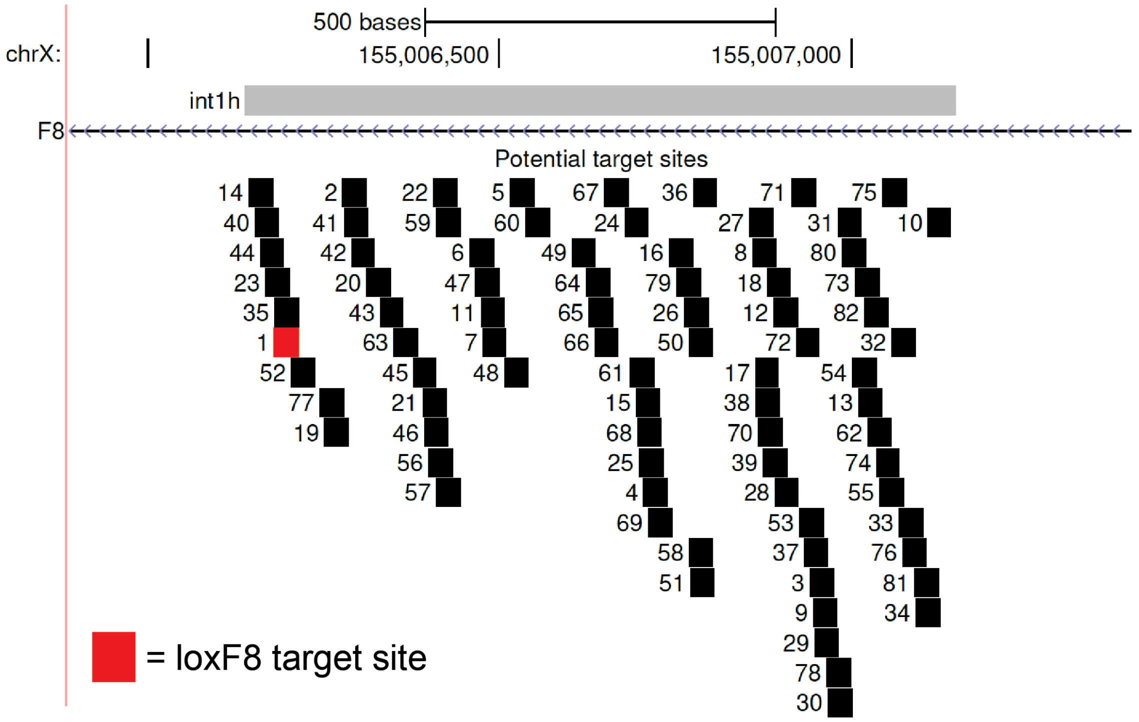

Supplementary Figure 1: loxF8 target site identification. Genome browser view of int1h-1 repeat (grey) on the X-chromosome with potential unique 34 bp recombinase target sites (black boxes). The target site that was used to evolve recombinases is marked in red. In total 82 potential target sites were nominated and sorted based on the number of mismatches and similarity to previously addressed target sites.

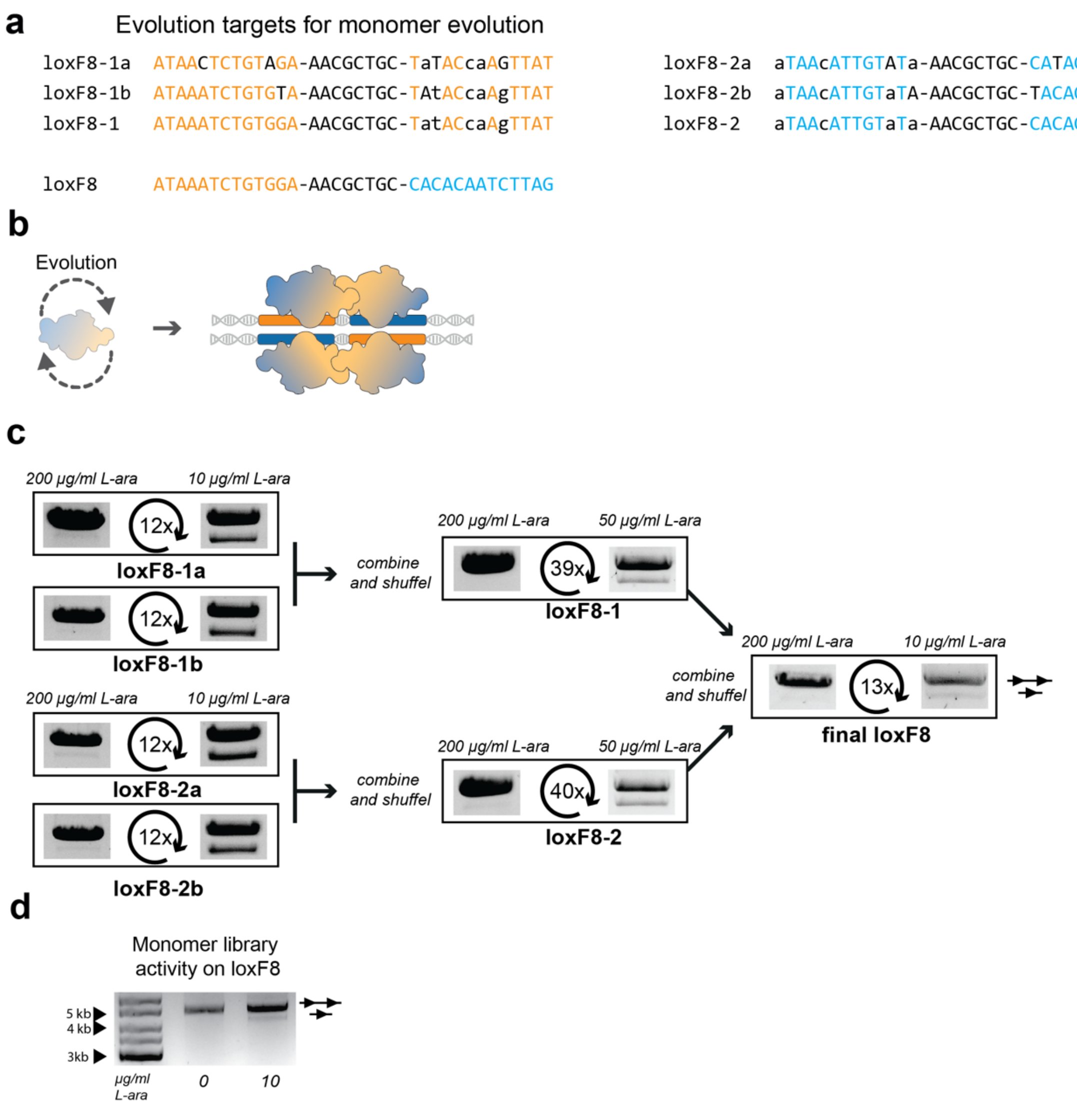

Supplementary Figure 2: Evolution targets and trajectory of the homotetramer evolution. a) loxF8 target site and the evolution subsites that were used for the homotetramer evolution. The left half-sites are depicted in orange and the right half-sites in blue. Differences compared to the loxF8 site or its half-sites are presented with black letters. Lower case letters show asymmetry in the evolution targets. b) Strategy to evolve a homodimer of recombinases to target the loxF8 site. The monomer is able to bind to both half-sites of the loxF8 target site. Recombinases are shown as homotetramers binding to two target sites in the recombination synapses. c) Schematic overview of the evolution of a monomer for the loxF8 target site. Evolutions for the subsites loxF8-1a, loxF8-1b, loxF8-2a and loxF8-2b were stepwise collapsed via loxF8-1 and loxF8-2 to finally obtain a recombinase library active on the loxF8 target site. The numbers indicate the evolutions cycles needed for each subsite. In total 183 evolution cycles were needed to acquire an active library that recombines loxF8 at low recombinase induction levels (1-10 µg/ml L-ara). For each subsite and final target site the starting and end activity is shown as a plasmid-based activity test digest. The recombinase expression levels are indicated above the gel picture. The upper band represents the unrecombined plasmid (illustrated by a line with two triangles) and the lower band shows the recombined plasmid (a line with one triangle). d) Plasmid-based activity assay of the homotetramer recombinase library evolved for recombining loxF8. The agarose gel picture of the final homotetramer library recombining the loxF8 site is shown at two different expression levels (0 and 10 ug/ml L-arabinose). The upper band represents the unrecombined plasmid (illustrated by a line with two triangles) and the lower band shows the recombined plasmid (a line with one triangle). Marker (M) and band sizes are indicated. Source data are provided as a Source Data file.

a

Evolution targets for heterodimer evolution

loxF8-L2	ATAAATCTGTGGA-AACGCTGC-TaCACcGATTTAT	loxF8-R2	CTAAcATTGTaTG-AACGCTGC-CACACAATCTTAG
loxF8-L1	ATAAATCTGTGGA-AACGCTGC-TCCACcGATTTAT	loxF8-R1	CTAAcATTGTGTG-AACGCTGC-CACACAATCTTAG
loxF8-L	ATAAATCTGTGGA-AACGCTGC-TCCACAGATTTAT	loxF8-R	CTAAGATTGTGTG-AACGCTGC-CACACAATCTTAG
loxF8	ATAAATCTGTGGA-AACGCTGC-CACACAATCTTAG		

b

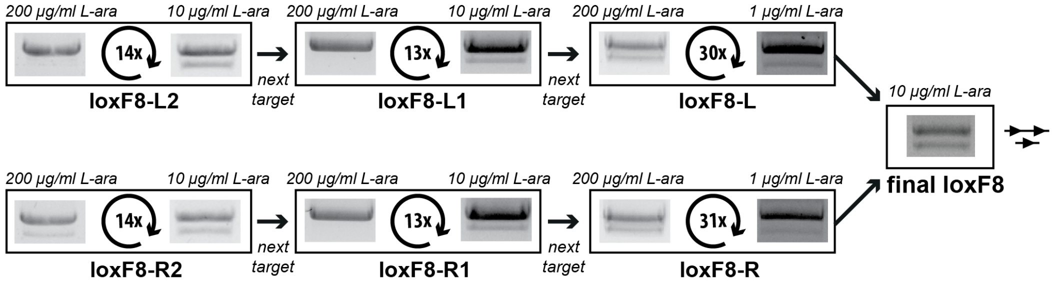

Supplementary Figure 3: Evolution targets and trajectory of the heterotetramer evolution. a) loxF8 target site and the evolution subsites that were used for the heterotetramer evolution. The left half-sites are depicted in orange and the right half-sites in blue. Differences compared to the loxF8 site or its half-sites are presented with black letters. Lower case letters show asymmetry in the evolution targets. b) Schematic overview of the evolution of two monomers that can be combined as heterotetramer recombining the loxF8 site. Independent evolutions were performed on the subsites loxF8-L2, loxF8-L1, loxF8-L, loxF8-R2, loxF8-R1 and loxF8-R. Libraries with activity on the loxF8-L (orange) and loxF8-R (blue) were co-expressed to recombine loxF8. The numbers indicate the evolutions cycles needed for each subsite. In total 115 evolution cycles were needed to acquire an active library that recombines loxF8 at low recombinase induction level. For each subsite and final target site the starting and end activity is shown as a plasmid-based activity test digest. The recombinase expression levels are indicated above the gel picture. The upper band represents the unrecombined plasmid (illustrated by a line with two triangles) and the lower band shows the recombined plasmid (a line with one triangle). Source data are provided as a Source Data file.

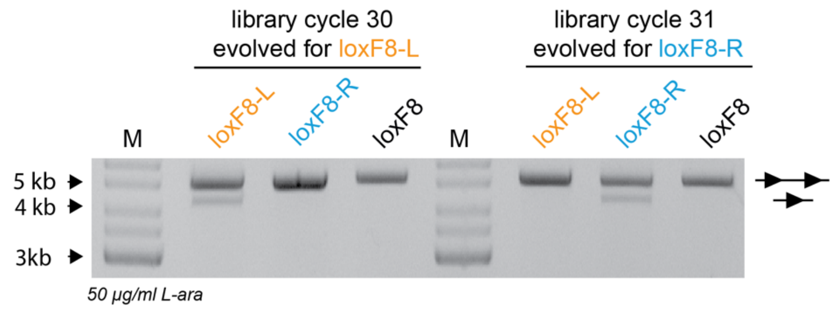

Supplementary Figure 4: Plasmid-based cross-reactivity of the final recombinase libraries evolved for either loxF8-L or loxF8-R. Both final libraries for loxF8-L or loxF8-R were expressed on the displayed target sites. The marker lane (M) and the band sizes are shown. The recombinase expression levels are indicated below the gel picture. The upper band represents the unrecombined plasmid (illustrated by a line with two triangles) and the lower band shows the recombined plasmid (a line with one triangle). Source data are provided as a Source Data file.

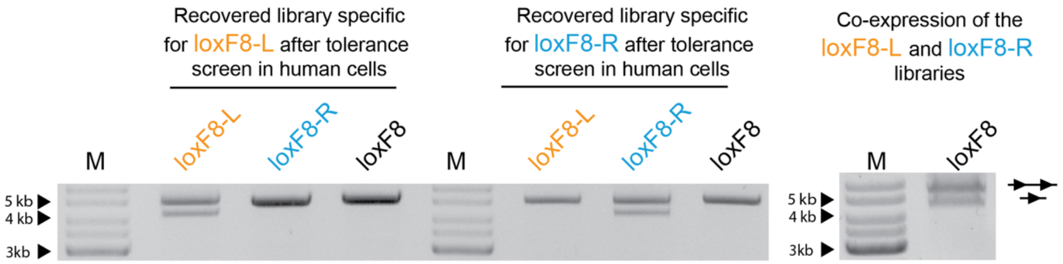

Supplementary Figure 5: Plasmid-based cross-reactivity of the final recombinase libraries evolved for either loxF8-L or loxF8-R after the tolerance selection in human cells. Both retrieved libraries for loxF8-L or loxF8-R were expressed in bacteria on the displayed target sites. Co-expression of both libraries is shown for on the loxF8 final target site. The marker lane (M) and the band sizes are shown. The recombinase expression levels are indicated below the gel picture. The upper band represents the unrecombined plasmid (illustrated by a line with two triangles) and the lower band shows the recombined plasmid (a line with one triangle). Source data are provided as a Source Data file.

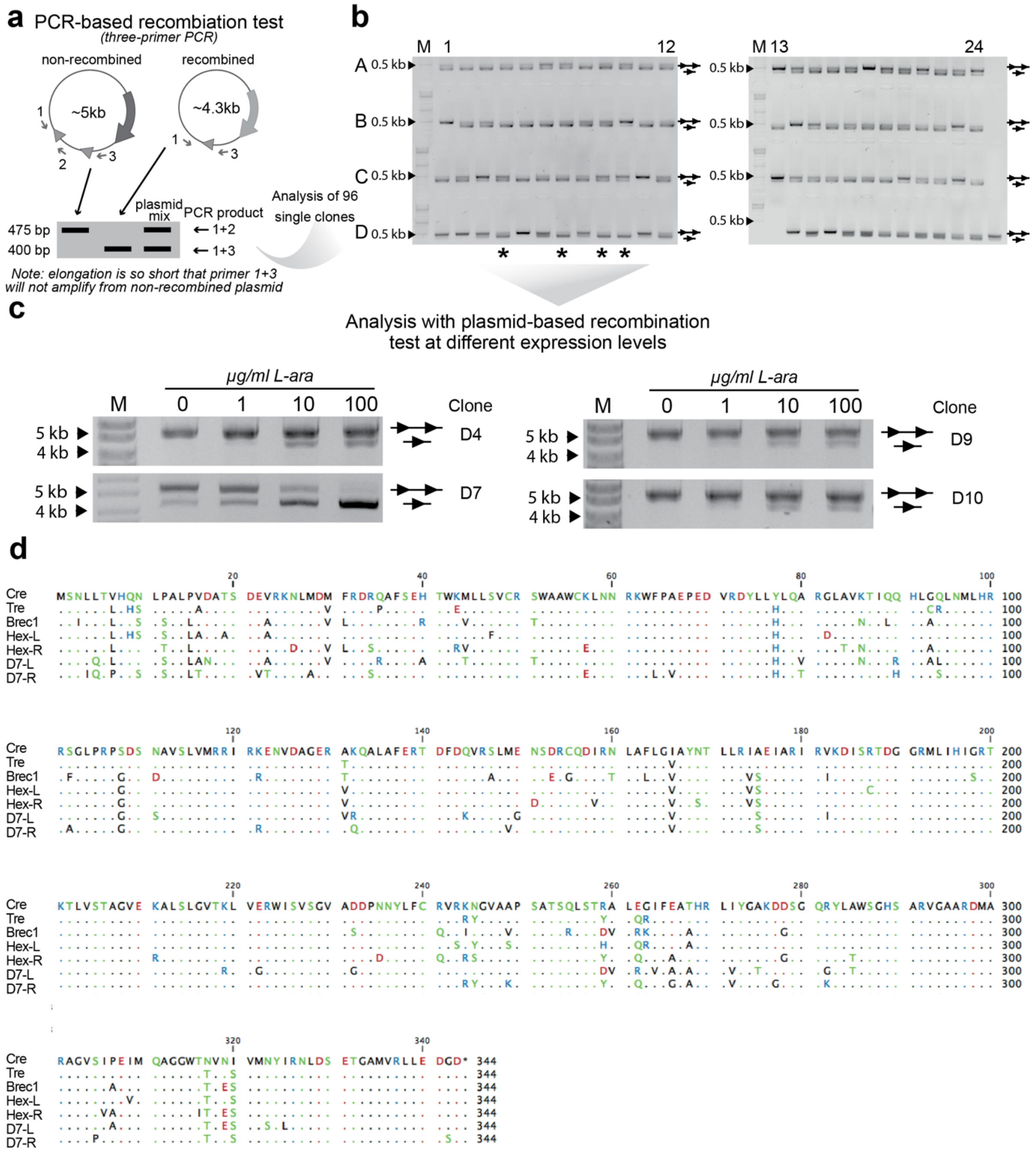

Supplementary Figure 6: Screening of single heterodimer clones that recombine loxF8 in bacteria. a) Schematic overview of the PCR-based recombination assay with the possible amplicons if non-recombined pEVO plasmid (475 bp), recombined pEVO plasmid (400 bp) or a mix of both plasmids are used as a template for the PCR. Grey numbered arrows show the primer binding sites and black triangles show the loxF8 target site in the pEVO vector. b) Ninety-six heterodimer clones were expressed in *E. coli* and analyzed with the PCR-based recombination assay. The marker lane (M) for 0.5 kb is indicated with an arrow. The upper band represents the PCR product from unrecombined plasmid (illustrated by a line with two triangles) and the lower band shows the PCR product from recombined plasmid (a line with one triangle). Clones that were further analyzed by a plasmid-based recombination assay are marked with asterisks (D4, D7, D9 and D10). c) Plasmid-based activity assay of the loxF8 candidate recombinase (D4, D7, D9 and D10). Recombinases were expressed in *E. coli* at four different L-arabinose concentrations (0, 1, 10 and 100 ug/ml). The upper band represents non-recombined substrate (line with two triangles), whereas the lower band shows the recombined plasmid (line with one triangle). The marker lanes (M) for 4 kb and 5 kb are indicated with arrows.d) Amino acid alignment of selected Cre-type recombinases compared to Cre. Tre, Brec1, Hex-L and HexR were previously evolved in the lab[16,17,21]. The alignment was performed using the CLC Genomics Workbench (https://wwww.qiagenbioinformatics.com). Residues were colored based on polarity. The same amino acids found in the reference sequence and the alignment are

represented as dots. Yellow - non-polar, green - polar uncharged, red - polar acidic, blue - polar basic. Source data are provided as a Source Data file.

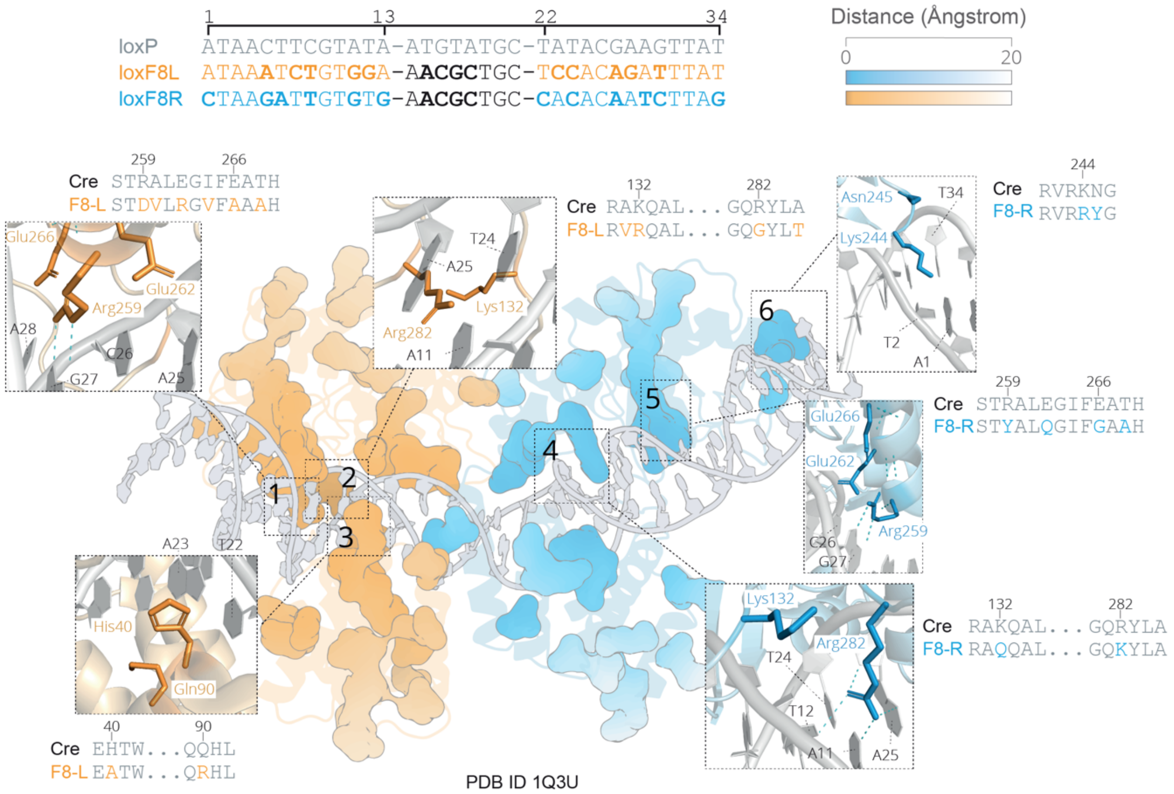

Supplementary Figure 7: Mapping of mutations to a Cre protein structure bound to loxP (model: PDB ID 1Q3U). Residues that have changed during the evolution in the D7 heterodimer are highlighted with the intensities of the color representing their proximity to DNA (dark = close, faint = far). Changes found in the left monomer are shown in orange and changes found in the right monomer are shown in blue. The protein structure was colored and adapted using Protein Imager (3dproteinimaging.com) {Tomasello:2020cy}. Boxes represent magnifications of six regions within the two monomers. The amino acid positions are displayed with the 3-letter amino acid code and the position number within Cre. Residues from the left monomer are colored in orange and residues from the right monomer are colored in blue. Nucleotide positions of the bound target site are indicated with the nucleotide and its position in the target site (e.g. box 1 A25, adenine at position 25 in loxP). A comparison of the amino acid sequence (1-letter code) to Cre is shown next to the boxes. Highlighted residues (orange or blue) show amino acid changes compared to Cre. The numbers indicate the amino acid position within Cre. On top of the structure the two symmetric evolution sites loxF8-L (blue) and loxF8-R (orange) are shown. Differences in the target site sequence are shown in bold. The nucleotides are numbered from left to right (1-34).

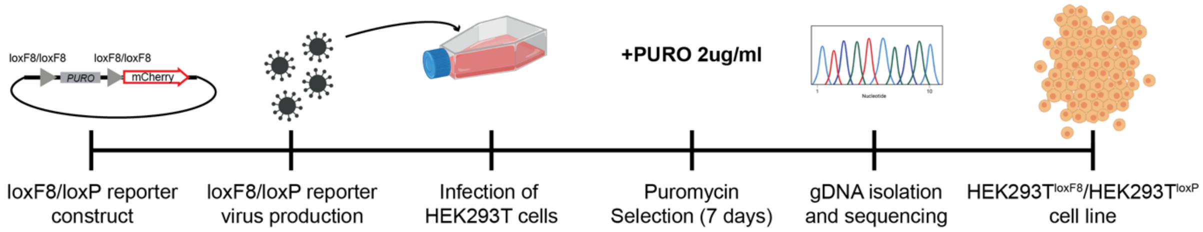

Supplementary Figure 8: Schematic overview of the generation of the HEK293T^{loxF8} and HEK293T^{loxP} reporter cell lines. A lentiviral reporter construct was cloned carrying a puromycin gene (PURO) flanked by lox sites (loxF8 or loxP). An out-of-frame mCherry gene is located downstream of the puromycin gene. HEK293T cells are infected with viral particles generated from the reporter construct. Successfully infected cells are selected by adding 2ug/ml puromycin to the culture medium for 7 days. The integrated reporter sequence was confirmed by sequencing.

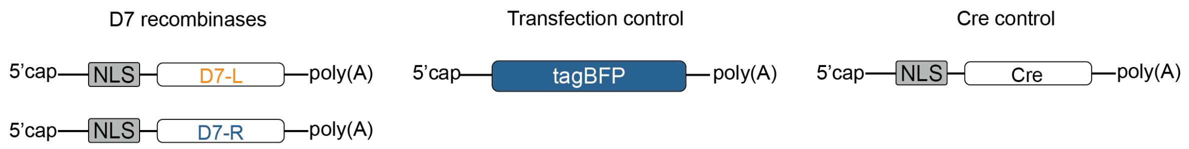

Supplementary Figure 9: Schematic representation of the mRNA used for transfection of human cells. To produce mRNA a T7 promoter, a SV40-NLS and a poly(A) polymerase signal is added to the recombinase coding sequence by PCR. During in vitro mRNA synthesis a 5'-cap and a 3'-poly(A) tail is added.

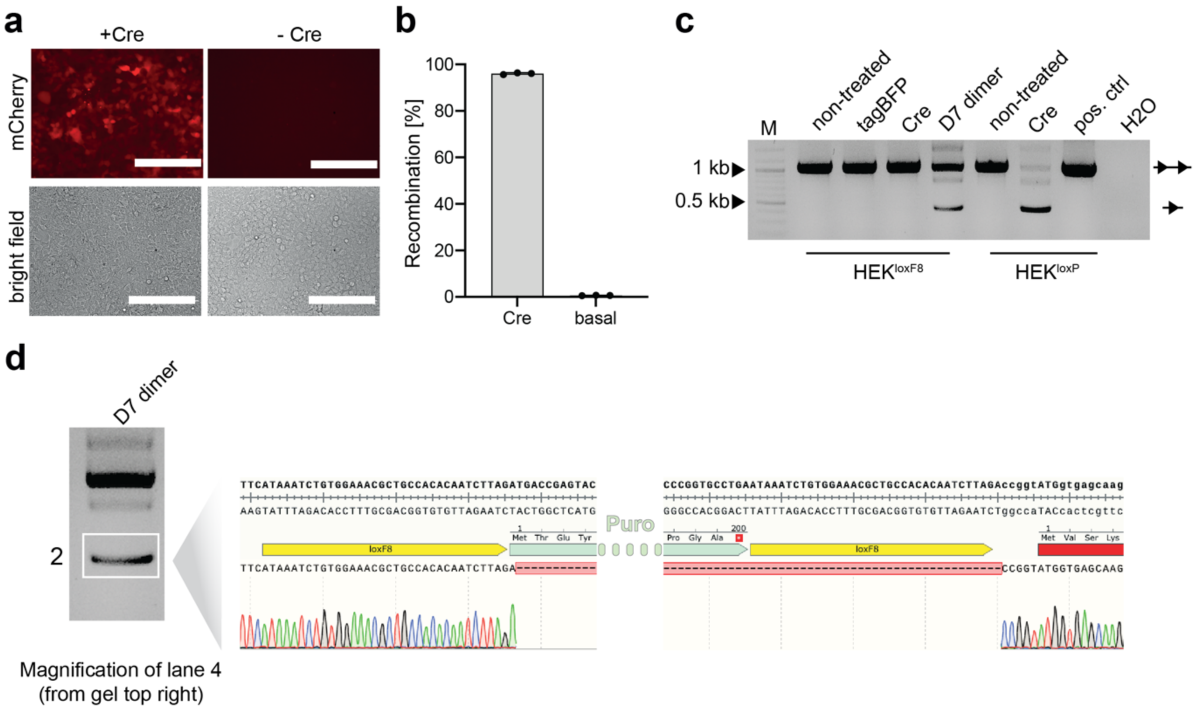

Supplementary Figure 10: Recombinase activity in HEK293T reporter cell lines. a) Fluorescent and brightfield images of transfected HEK293T^{loxP} reporter cells with and without Cre. 200 µm scale bars are indicated. b) Quantification of the recombination efficiency 48 h after transfection of HEK293T^{loxP} reporter cells with and without Cre, analyzed by flow cytometry (n = 3, biological replicates are shown as dots). Error bars represent standard deviation of the mean (SD). c) Validation of the right recombination product of the integrated reporter construct of the HEK293T^{loxF8} and HEK293T^{loxP} cell lines. Gel picture of PCR products obtained when using genomic DNA as template of the indicated cell lines are shown. The upper band represents the non-recombined reporter construct. The lower band shows the recombined reporter construct. Only when expressing D7 or Cre, the lower PCR bands are visible. d) Sequencing reads of the recombined PCR product generated from HEK293T^{loxF8} reporter cells transfected with D7. The loxF8 target sites are indicated as well as the puromycin gene (PURO) which is excised upon recombinase activity. Source data are provided as a Source Data file.

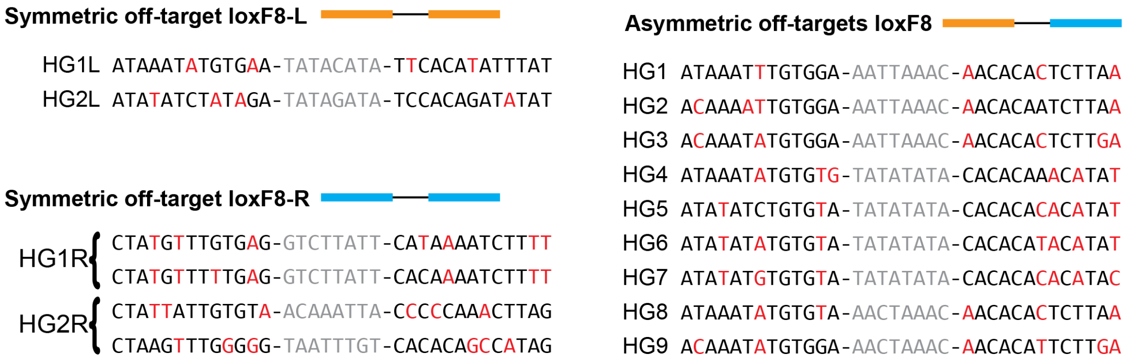

Supplementary Figure 11: Bioinformatically predicted off-targets (asymmetric and symmetric). Nucleotide sequences of computationally predicted off-targets with high similarity to loxF8, loxF8-L and loxF8-R are shown. Differences to the respective target sites (loxF8) are marked in red.

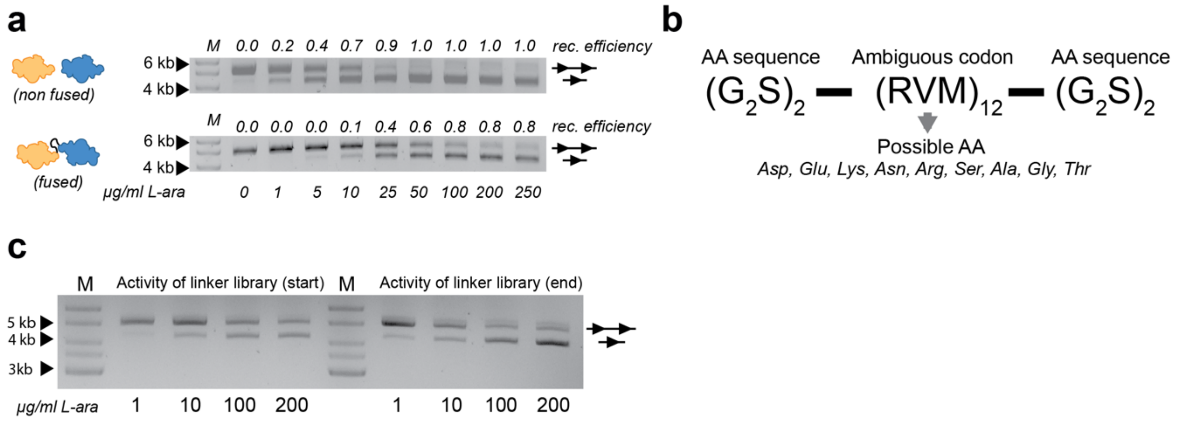

Supplementary Figure 12: Linker library and selection for fusion of the D7 monomers. a) Dose dependent activity of the non-fused and fused (GGS_8-linker) D7 dimer at different expression levels. The upper band represents non-recombined substrate whereas the lower band shows recombination. L-arabinose induction levels are shown on the bottom. The recombination efficiencies were calculated from the ratios of the intensities of the bands using Fiji {Schindelin:2012ir}. b) Linker library sequence used to create a pool of differently linked recombinases (the recombinases are always the same). c) Activity of the fused recombinases with the linker library before and after directed evolution of the linker at indicated L-arabinose induction levels on loxF8. Source data are provided as a Source Data file.

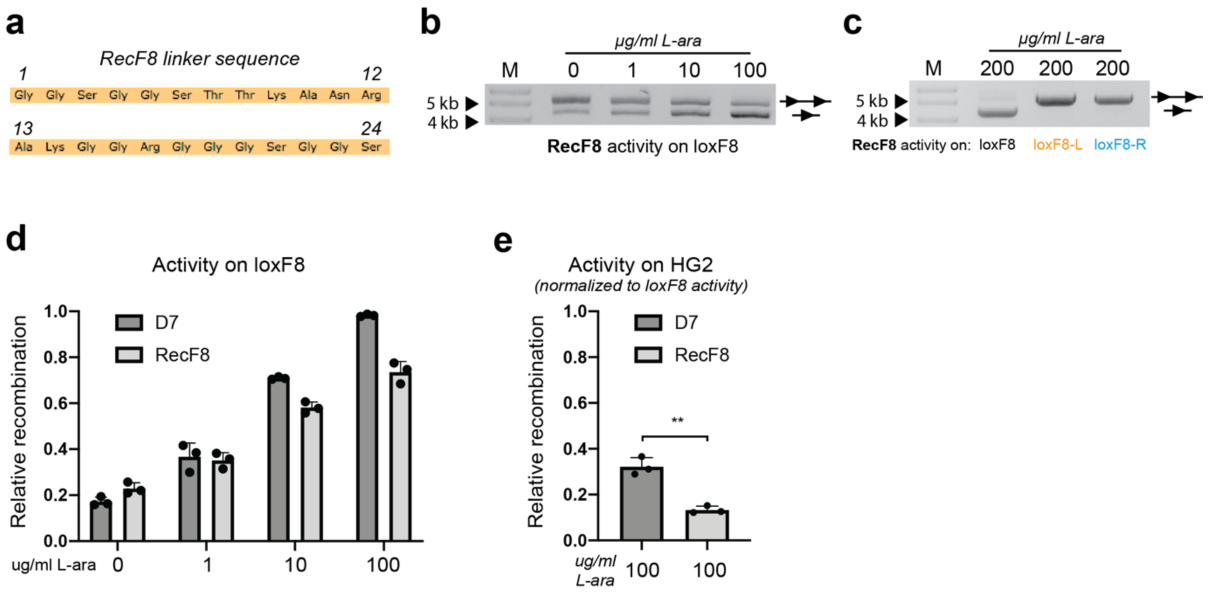

Supplementary Figure 13: Characteristics of RecF8 fused heterodimer. a) Linker sequence of RecF8 shown as 3-letter amino acid code. The order is indicated by numbers. b) Plasmid-based activity assay of RecF8. RecF8 was expressed in *E. coli* at three different L-arabinose concentrations (1, 10 and 100 ug/ml). The upper band represents non-recombined substrate (line with two triangles), whereas the lower band shows the recombined plasmid (line with one triangle). The marker lane (M) for 4 kb and 5 kb is shown with arrows. c) Cross-reactivity of RecF8 on the symmetric target sites loxF8-L and loxF8-R at high induction level (200 ug/ml L-arabinose). d) Quantification of dose depended activity of D7 and RecF8 at different induction levels (0, 1, 10, 100 ug/ml L-arabinose). The recombination efficiencies are calculated by the ratio of the intensity of the non-recombined and recombined band from three independent biological replicates. e) Quantification of D7 and RecF8 activity on the HG2 off-target. The recombination efficiencies are calculated by the ratio of the intensity of the non-recombined and recombined band from three independent biological replicates. The recombination of HG2 is normalized to the activity of D7 or RecF8 on loxF8 at the same induction level. *$p < 0.05$; **$p < 0.01$, ***$p < 0.001$ using unpaired two-sided t-test (two-stage step up method, Prism 8). p value = 0.001582. Source data are provided as a Source Data file.

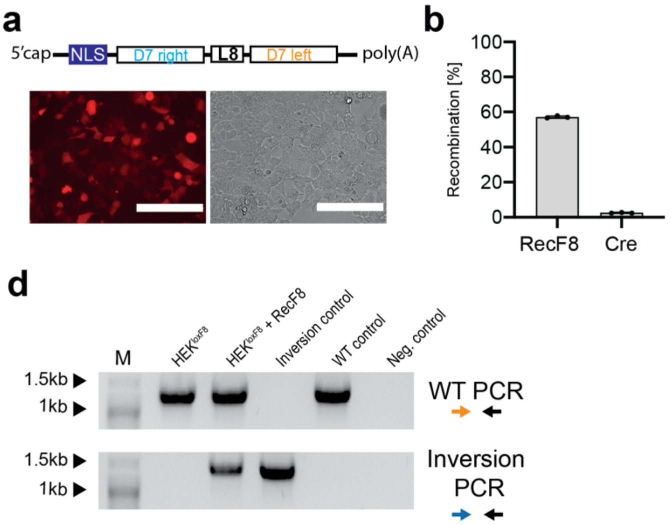

Supplementary Figure 14: RecF8 activity in HEK293T^{loxF8} reporter cell line. a) Fluorescent and brightfield images of transfected HEK293T^{loxF8} reporter cells with RecF8. 200 µm scale bars are indicated. b) Quantification of the recombination efficiency 48 h after transfection of HEK293T ^{loxF8} reporter cells with RecF8, or Cre as control, analyzed by flow cytometry (n = 3, biological replicates are shown as dots). Error bars represent standard deviation of the mean (SD). c) Schematic overview of a fraction of the F8 gene displaying the PCR primers used to detect the orientation of the loxF8 locus. Exons are displayed in magenta and the repeated regions int1h-1 and int1h-2 are shown in grey. Primer binding sites are indicated with arrows. The transcription start site of the F8 gene is depicted by a black arrow. d) Gel image of PCR products generated using indicated primer combinations to detect the orientation of the loxF8 locus with and without treatment with RecF8 heterodimer. Marker (M) lanes at 1 kb and 1.5 kb are indicated. WT = iPSCs from a donor that does not carry the exon 1 inversion of the F8 gene. F8 = iPSCs from a hemophilic donor carrying the exon 1 inversion of the F8 gene. Neg. = water control. Source data are provided as a Source Data file.

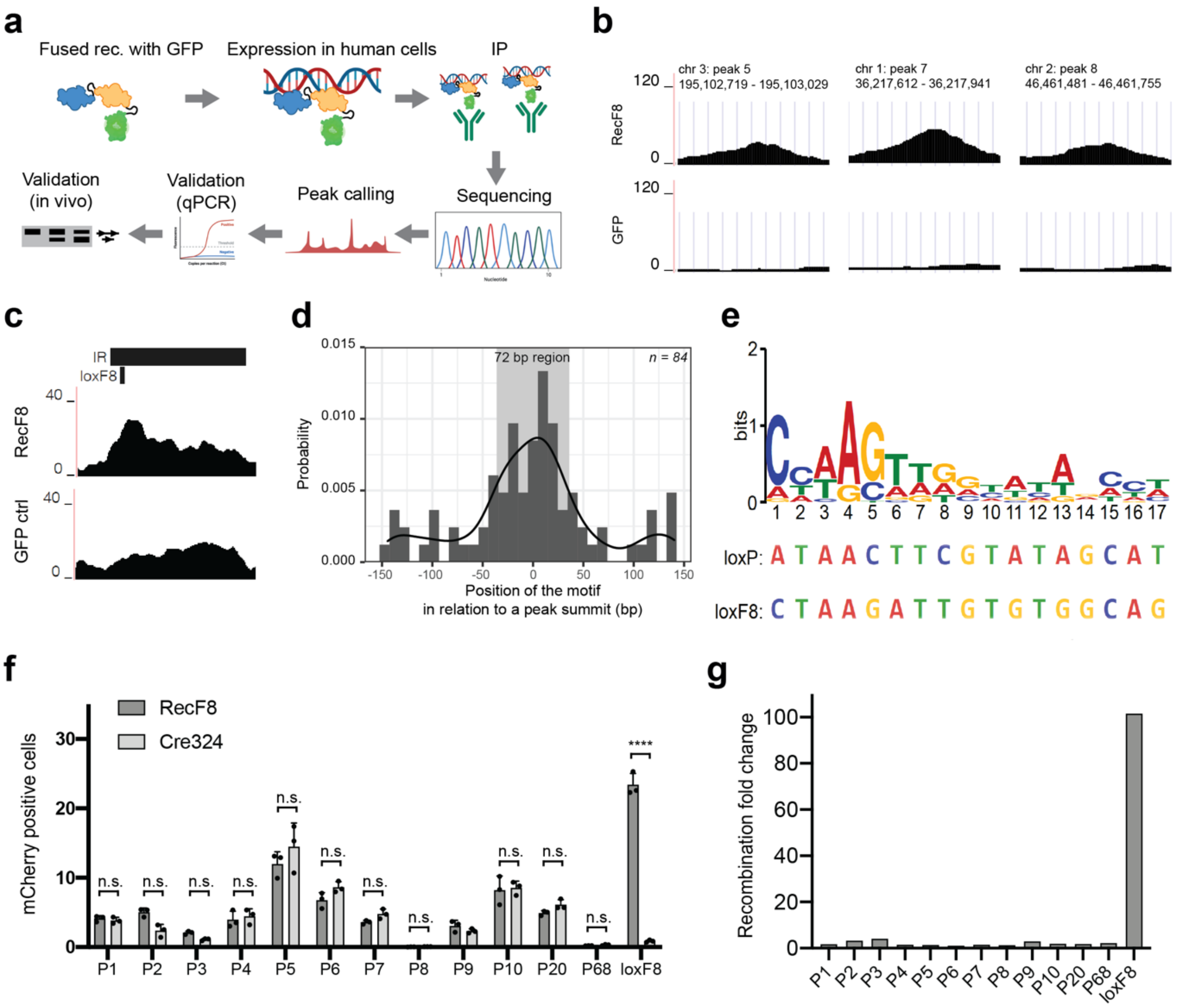

Supplementary Figure 15: Identification and validation of RecF8 binding sites using ChIP-seq. a) Pipeline of the ChIP-seq experimental procedure to identify RecF8 binding sites in the human genome. Important steps are indicated by arrows. b) Exemplary ChIP-seq. pile ups at three different loci for RecF8 and GFP control sample. c) Genome browser view of raw reads of ChIP-Seq. data at the genomic loxF8 target site for RecF8 and GPF control samples. The 1kb inverted repeat (IR) is indicated with the location of the loxF8 target site. d) lox-like target site motif analysis of the peak summits identified by ChIP-Seq. The x-axis indicates the distance (bp) of the lox-like motif from the summit. The probability of finding a motif is shown on the y-axis. The highest probability of finding a lox-like motif is in a window of 72 bp surrounding the peak summit (highlighted in grey). e) Motif consensus of the ChIP-seq peaks. The height of the letters represents the abundance at each position. Numbers below show the 5'-3' position of the identified motif. The target sites of Cre (loxP) and RecF8 (loxF8) are depicted below the motif. f) Human plasmid-based RecF8 activity assay of the twelve RecF8-binding sequences. Ninety-five bp surrounding the peak summit of twelve peaks were cloned as an excision substrate in the human fluorescent reporter vector. Recombination (mCherry positive cells) of the loxF8 site is shown as a reference. Inactive Cre324 mutant was included as a control to account for the leakiness of the reporter plasmid. n.s. = not significant, *$p < 0.05$; **$p < 0.01$, ***$p < 0.001$, ****$p < 0.0001$ using unpaired two-sided t-test (two-stage step up method, Prism 8). loxF8 p value = 0.000235. g) Fold-change of the peak recombination efficiency when comparing cells transfected with RecF8 to cells transfected with inactive Cre324 mutant. Source data are provided as a Source Data file.

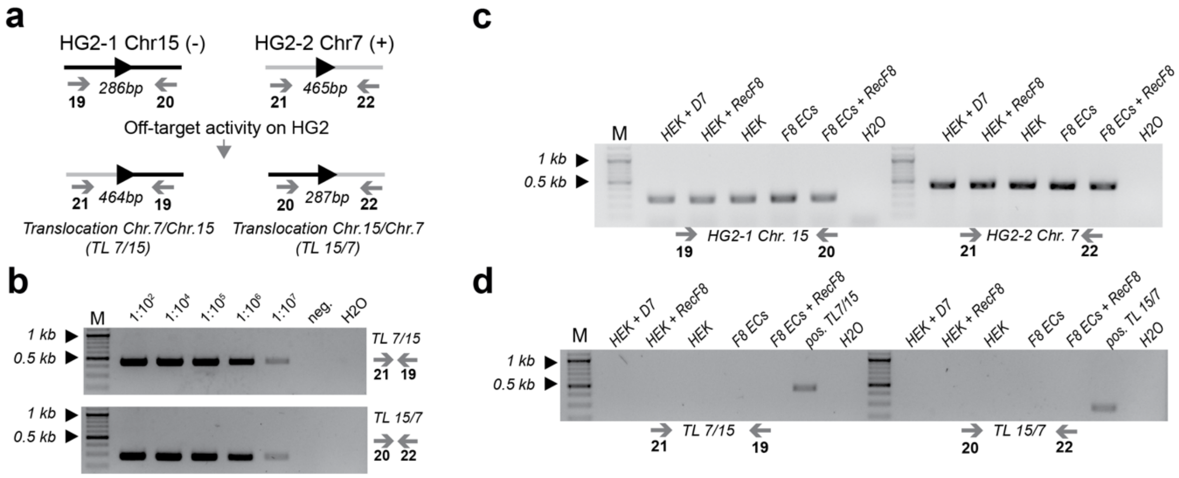

Supplementary Figure 16: Recombination test of HG2 in human cells. a) Scheme of a PCR assay that would detect RecF8-mediated recombination of the asymmetric HG2 off-target site on chromosomes 7 and 15. Successful recombination would lead to a translocation between chromosome 7 and 15 (TL7/15 and TL15/7). The PCR product sizes are indicated for each PCR primer combination. b) Sensitivity of the assay on synthesized DNA fragments that resembles the translocation products 7/15 and 15/7. Dilutions of the DNA fragments are indicated in the top lane. E.g., 1:10^2 equals 1 copy of the translocation product with 100 copies of the non-translocated product of the HG2 off-target site. Marker (M) lanes at 0.5 kb and 1 kp are indicated. Primer binding sites are depicted with grey arrows. c) Gel image of the PCR products obtained from human cells treated with and without RecF8 to detect the normal integrity of the HG2 off-target locus. Marker (M) lanes at 0.5 kb and 1 kb are indicated. Primer binding sites are depicted with grey arrows. d) Gel image of the PCR products obtained from human cells treated with and without RecF8 to detect the translocation of the HG2 off-target locus after recombination. The synthesized fragments to resemble TL7/15 and 15/7 are included at a ratio of 1:200 genome equivalence as positive controls. Marker (M) lanes at 0.5 kb and 1 kp are shown. Primer binding sites are depicted with grey arrows. Source data are provided as a Source Data file.

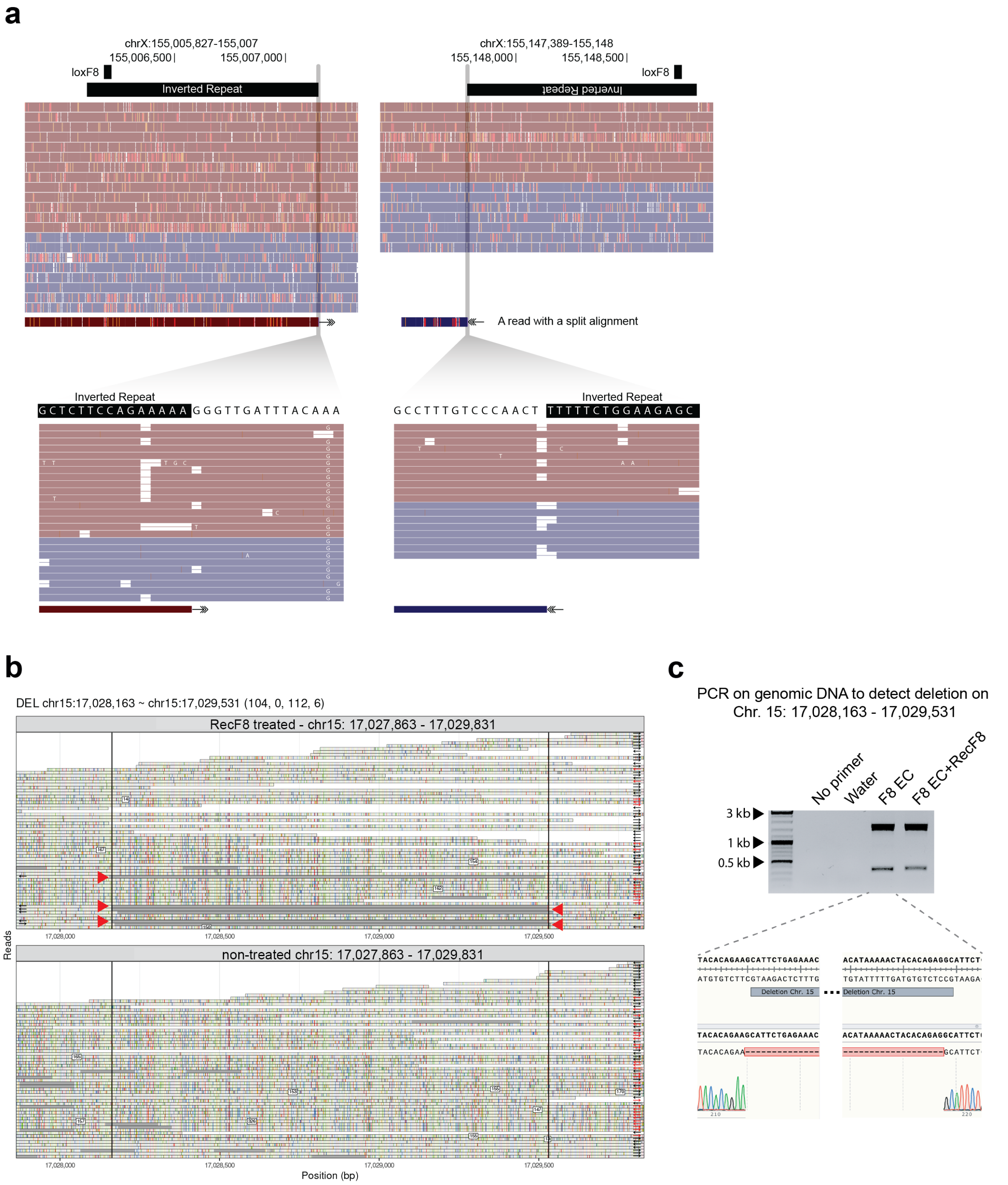

Supplementary Figure 17: Variant calling in the whole genome sequencing data. a) Genome browser views showing two regions spanning inverted repeats (IRs) containing loxF8 sites (depicted as black bars) and sequencing reads aligning at their locations. Red and blue bars indicate reads aligning to the forward and reverse strand respectively. Colored stripes scattered along the reads indicate mismatches and short INDELs. The last read, shown in darker colors, shows a split alignment, where one part of the read aligns with the first IR and one part aligns with the sequence downstream of the second IR, in a reverse orientation. Breakpoint positions are indicated by arrows. Breakpoints of the split read perfectly coincide with ends of the IRs, which indicates that the read originates from an inverted allele. Zoom-ins below show 30 bp sequences centered around ends of the sequence lying between the IRs. Breakpoints of the split read perfectly coincide with ends of the IRs, which indicates that the read originates from an inverted allele. b) Example of long sequencing reads obtained from the Nanopore whole genome sequencing. The upper panel shows reads of a locus on chromosome 15 from RecF8-treated cells and the lower panel shows reads at the same locus from non-treated cells. Six reads in the upper panel support the presence of a deletion at this position (marked with an arrow). c) Gel picture of the PCR product obtained from genomic DNA of cell treated with and without RecF8 when amplifying over the deletion locus on chromosome 15.

Two bands are visible in both samples indicating the presence of the non-deleted and deleted locus independent of RecF8 expression. The deletion band was sequenced and confirmed the results obtained by the Nanopore whole genome sequencing. Marker (M) and band sizes are indicated. Source data are provided as a Source Data file.

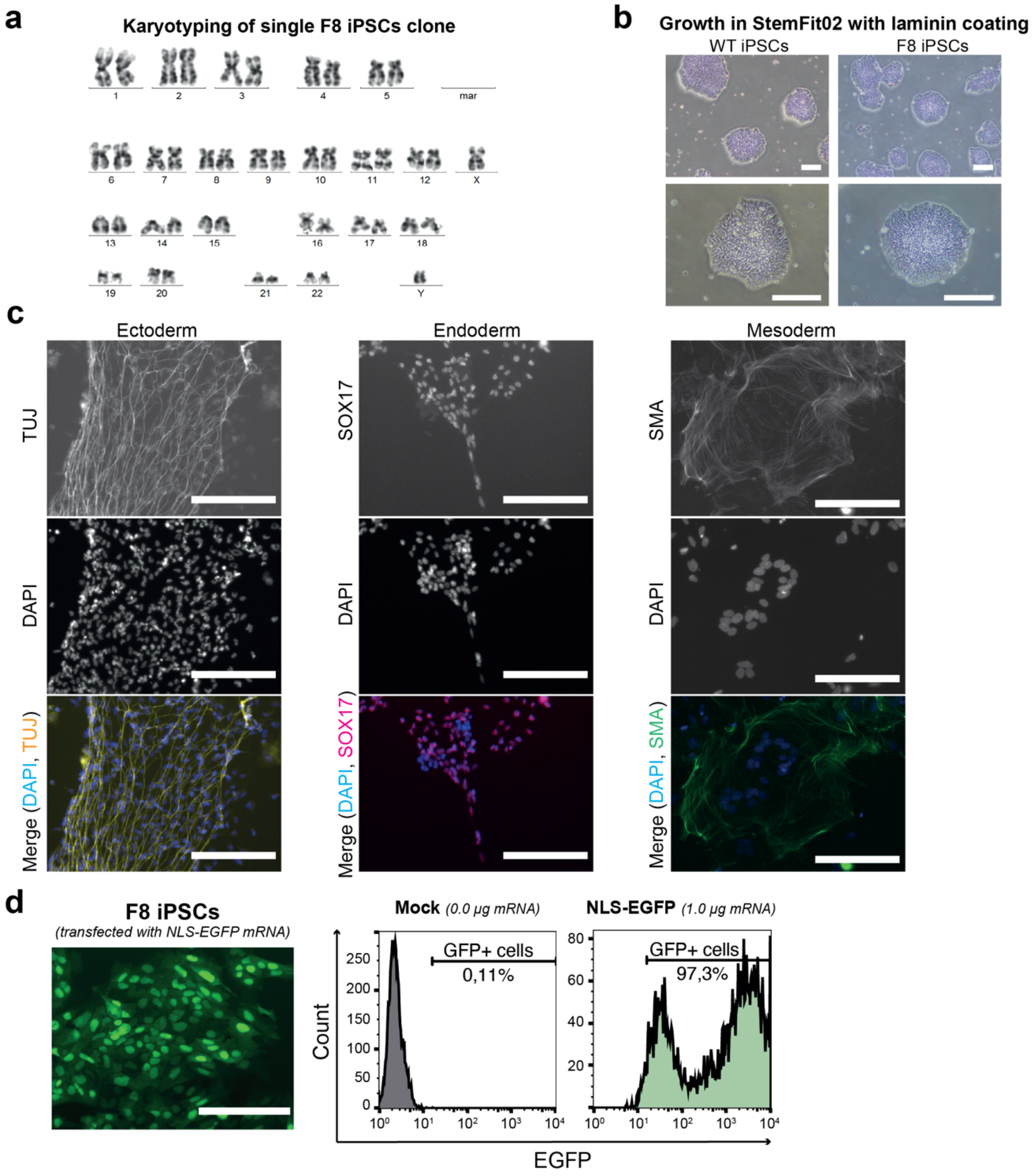

Supplementary Figure 18: Characterization of patient specific iPSCs derived from blood cells. a) Karyotyping of patient derived iPSCs. A normal male karyotype without abnormalities is seen for the patient derived iPSCs. b) Morphological comparison of patient derived iPSCs to WT iPSCs cell line grown in StemFit02 with laminin coating. White bars indicate 200 µm scale bars. c) Differentiation capacity of patient derived iPSCs to ectoderm, endoderm and mesoderm. The ectodermal marker beta-III-tubulin (TUJ) was used to show ectodermal differentiation capacity. The endodermal marker SRY-related HMG-box 17 (SOX17) was used to show endodermal differentiation capacity. The mesodermal marker smooth muscle actin (SMA) was used to show mesodermal differentiation capacity. DAPI was used in all three differentiations to stain the nucleus of the cells. The black and white images of the staining's as well as the merge images with the respective colors are shown. White bars indicate 200 µm scale bars. d) Transfection of patient derived induced pluripotent stem cells (iPSCs) with NLS-EGFP mRNA. A representative microscopy image of 48 h post transfection with NLS-EGFP mRNA is shown. White bar indicates 200 µm scale bar. A FACS histogram of mock and NLS-EGFP transfected iPSCs 48 h post transfection is shown to the right. NLS, nuclear localization sequence. Source data are provided as a Source Data file.

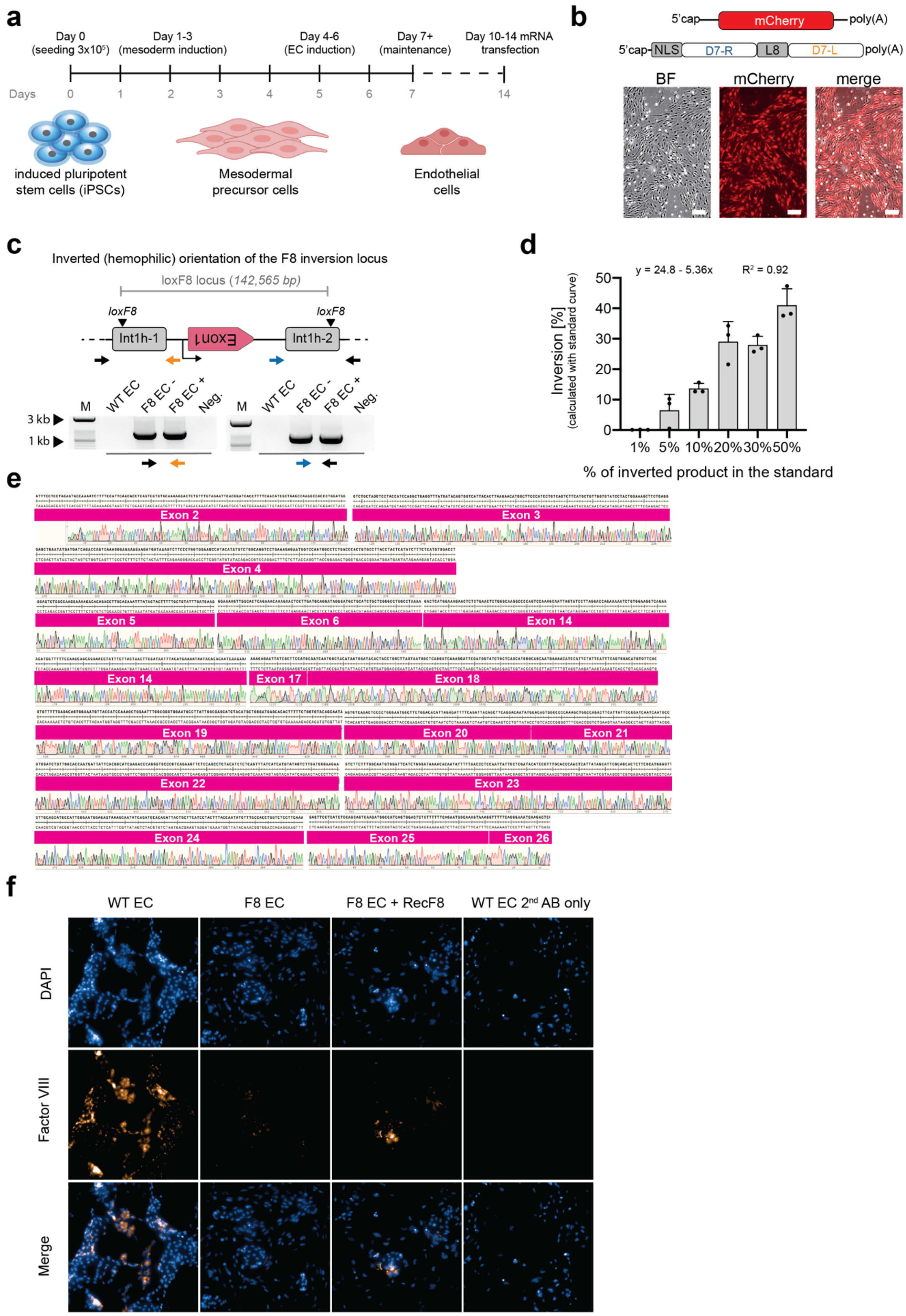

Supplementary Figure 19: a) Schematic overview of the EC differentiation protocol. Important steps are highlighted on the timeline. b) Efficiency of transfection of mCherry mRNA to differentiated endothelial cells. Brightfield (BF) image, mCherry image and the merged image a shown. Images were taken 48 h post transfection. White bars indicate 50 µm scale bar. c) Confirmation of the inverted orientation of the loxF8 locus in differentiated ECs from patient specific iPSCs. The arrangement of different primers around the

first and second loxF8 site to detect the orientation of the full 140 kb fragment before and after RecF8 treatment are shown in the top panels. The lower panels show gels of PCR products obtained on genomic DNA from patient specific ECs with and without treatment with RecF8. The combination of the primers used for every PCR gel picture is shown below. Marker sizes of 1kb and 3kb are indicated. WT = ECs differentiated from a donor that does not carry the exon 1 inversion of the F8 gene. F8 = ECs differentiated from a hemophilic donor carrying the exon 1 inversion of the F8 gene. Neg. = water control. d) qPCR-based inversion measurement of a defined standard dilution to extrapolate a standard curve formula (y = 24.8 - 5.36x) for calculating the inversion efficiencies of the genomic loxF8 locus. The standard was prepared by mixing genomic DNA from WT and F8 patient iPSCs in defined ratios (1%, 5%, 10%, 25% and 50% normal orientation of the loxF8 locus, n=3, replicates are shown as dots). The standard curve formula is shown on top of the graph. Error bars represent standard deviation of the mean (SD). e) Sequencing reads of the Factor VIII transcript (cDNA) generated from patient specific ECs treated with RecF8. Exons are marked in magenta and numbered. The sequence and the chromatogram for each read are indicated. f) Immunocytochemical staining for Factor VIII (F8) of fixed ECs differentiated from WT or F8 iPSCs. WT EC = positive control, F8 EC = non-treated patient specific EC, F8 EC + RecF8 = patient specific EC treated with RecF8 mRNA for 72 h, WT EC 2nd AB only = positive control where only the secondary antibody was applied. DAPI in blue, Factor VIII in orange. Source data are provided as a Source Data file.

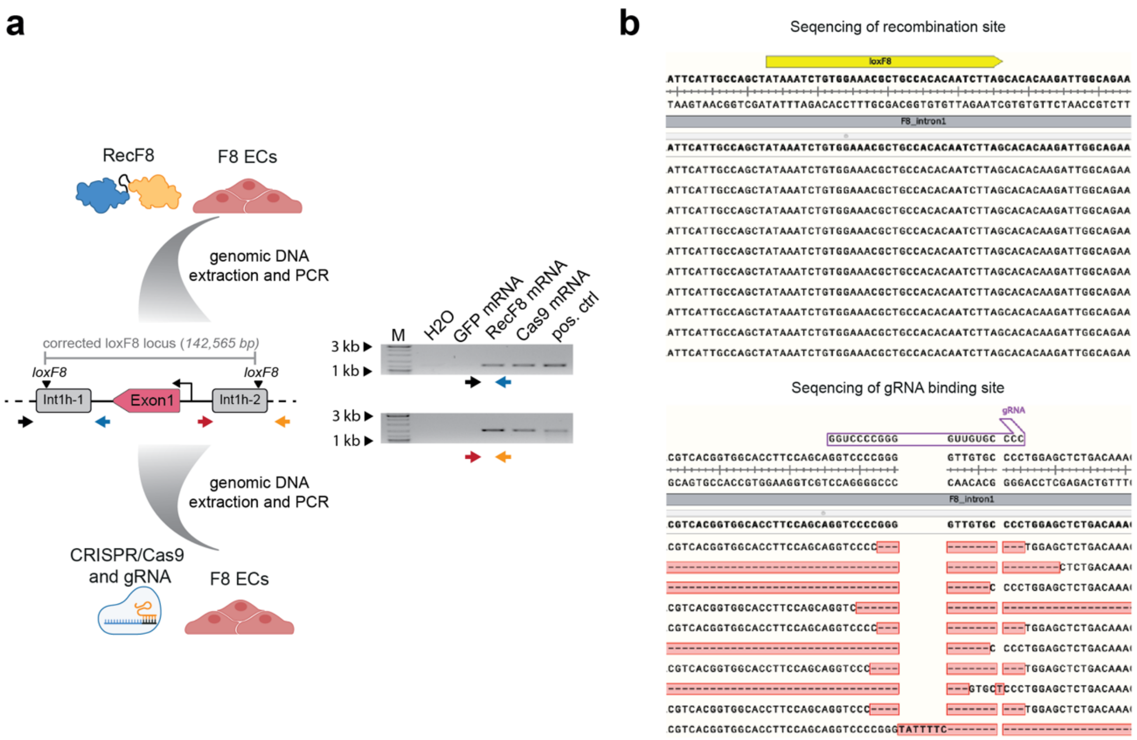

Supplementary Figure 20: Clonal analysis of inversion fidelity of the loxF8 locus after treatment with RecF8 or CRISPR/Cas9. a) Schematic overview of the clonal analysis after RecF8 or CRISPR/Cas9 treatment with subsequent PCR to amplify the inversion product. M = Marker, H2O = water control. GFP mRNA = genomic DNA of F8 EC transfected with GFP mRNA, RecF8 mRNA = genomic DNA of F8 EC transfected with RecF8 mRNA, Cas9 mRNA = genomic DNA of F8 EC transfected with Cas9 mRNA and gRNA control, pos. ctrl = genomic DNA of a health individual. b) 10 clonal exemplary sequencing reads are shown for RecF8 treated and CRISPR/Cas9 treated samples. The loxF8 binding site is depicted in yellow. The gRNA binding site is depicted above in purple. Deletions are marked by dashes in red boxes. Source data are provided as a Source Data file.

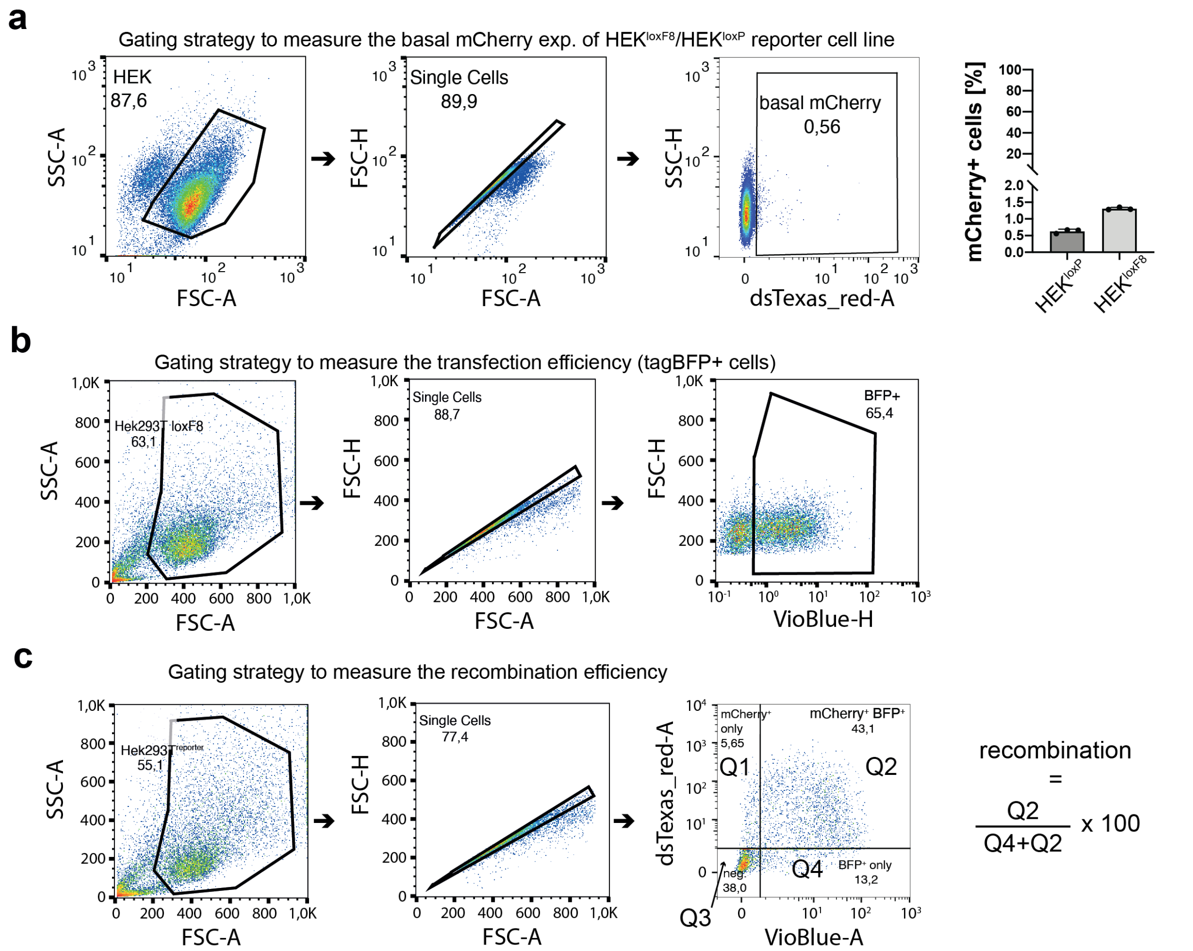

Supplementary Figure 21: Gating strategies for the analysis by flow cytometry. a) Exemplary gating strategy for basal levels of mCherry expression in the reporter cell lines HEK^{loxF8} and HEK^{loxP} b) Exemplary gating strategy for the transfection efficiency. c) Exemplary gating strategy for the recombination efficiency. The recombination efficacy is calculated using following formula: recombination = %Q2/(%Q4+%Q2) x 100. Source data are provided as a Source Data file.

Supplementary Table 1: Cycling programs used for different PCRs.

Count	Cycling Protocol	Temperature (°C)	Time (s)	Cycles
1	F8 inversion PCR	94	45	
		94	20	40x
		52	20	
		72	45	
		72	300	
		8	infinite	
2	qPCR to qunatify the inversion	94	180	
		94	20	50x
		56	20	
		72	180	
3	In vitro transcription	37	30	
		8	pause	
		37	15	add Dnase
		8	pause	
		37	30	add poly(A) polymerase
		8	infinite	
4	Peak insert generation	94	15	30x
		58	15	
		72	30	
		72	300	
		8	infinite	
5	HG2 translocation	94	15	30x
		56	15	
		72	20	
		72	300	
		8	infinite	
