# Supplementary material for: Correction of a Factor VIII genomic inversion with designer-recombinases
Source: Nat Commun. 2022 Jan 20;13:422. doi: 10.1038/s41467-022-28080-7 (PMC8776779; doi:10.1038/s41467-022-28080-7)
Supplement: Supplementary file 2 — Reporting Summary [file 41467_2022_28080_MOESM2_ESM.pdf]

nature research

Corresponding author(s): Frank Buchholz

Last updated by author(s): Dec 14, 2021

Reporting Summary

Nature Research wishes to improve the reproducibility of the work that we publish. This form provides structure for consistency and transparency in reporting. For further information on Nature Research policies, see our Editorial Policies and the Editorial Policy Checklist.

Statistics

For all statistical analyses, confirm that the following items are present in the figure legend, table legend, main text, or Methods section.

n/a	Confirmed	
☐	☒	The exact sample size (*n*) for each experimental group/condition, given as a discrete number and unit of measurement
☐	☒	A statement on whether measurements were taken from distinct samples or whether the same sample was measured repeatedly
☐	☒	The statistical test(s) used AND whether they are one- or two-sided<br>*Only common tests should be described solely by name; describe more complex techniques in the Methods section.*
☒	☐	A description of all covariates tested
☒	☐	A description of any assumptions or corrections, such as tests of normality and adjustment for multiple comparisons
☐	☒	A full description of the statistical parameters including central tendency (e.g. means) or other basic estimates (e.g. regression coefficient) AND variation (e.g. standard deviation) or associated estimates of uncertainty (e.g. confidence intervals)
☐	☒	For null hypothesis testing, the test statistic (e.g. *F*, *t*, *r*) with confidence intervals, effect sizes, degrees of freedom and *P* value noted<br>*Give P values as exact values whenever suitable.*
☒	☐	For Bayesian analysis, information on the choice of priors and Markov chain Monte Carlo settings
☒	☐	For hierarchical and complex designs, identification of the appropriate level for tests and full reporting of outcomes
☒	☐	Estimates of effect sizes (e.g. Cohen's *d*, Pearson's *r*), indicating how they were calculated

Our web collection on statistics for biologists contains articles on many of the points above.

Software and code

Policy information about availability of computer code

Data collection	no software was used
Data analysis	EMBOSS Water v6.6.0 (http://emboss.sourceforge.net/), PatMaN v1.2.2 (http://bioinf.eva.mpg.de/patman), BEDTools suite v2.29.2 (https://github.com/arq5x/bedtools2), STAR v2.7.3a (https://github.com/alexdobin/STAR), Samtools v1.10 (http://www.htslib.org/), Genrich (https://github.com/jsh58/Genrich), minimap2 v2.17 (https://github.com/lh3/minimap2), Sniffles v1.0.12 (https://github.com/fritzsedlazeck/Sniffles), SURVIVOR v1.0.7 (https://github.com/fritzsedlazeck/SURVIVOR), bcftools v1.10 (https://github.com/samtools/bcftools)

For manuscripts utilizing custom algorithms or software that are central to the research but not yet described in published literature, software must be made available to editors and reviewers. We strongly encourage code deposition in a community repository (e.g. GitHub). See the Nature Research guidelines for submitting code & software for further information.

Data

Policy information about availability of data

All manuscripts must include a data availability statement. This statement should provide the following information, where applicable:

- Accession codes, unique identifiers, or web links for publicly available datasets
- A list of figures that have associated raw data
- A description of any restrictions on data availability

The datasets generated during and/or analyzed during the current study are not publicly available due to the current filing process of the patent but are available from the corresponding author F.B. on reasonable request. The patient specific WGS data are available under restricted access due to data privacy laws, but may be obtained with Data Use Agreements with the Technical University of Dresden, Germany. Researchers interested in access to the data may contact F.B. at frank.buchholz@tu-dresden.de. It can take some months to negotiate data use agreements and gain access to the data. The author will assist with any reasonable replication attempts for two years following publication. After positive request the WGS data access is available and can be accessed at the European Genome-

phenome Archive (https://ega-archive.org/) under a Study ID: EGAS00001005496. The ChIP-Seq data generated in this study have been deposited in the at the NCBI Gene Expression Omnibus (https://www.ncbi.nlm.nih.gov/geo) under a Series ID:GSE159492. Other data generated in this study are provided in the Supplementary Information/Source Data file.

Field-specific reporting

Please select the one below that is the best fit for your research. If you are not sure, read the appropriate sections before making your selection.

☒ Life sciences ☐ Behavioural & social sciences ☐ Ecological, evolutionary & environmental sciences

For a reference copy of the document with all sections, see nature.com/documents/nr-reporting-summary-flat.pdf

Life sciences study design

All studies must disclose on these points even when the disclosure is negative.

Sample size	Sample sizes were determined based on other studies in the field of genome editing (e.g. Karpinski et. al 2016 or Anzalone et al. 2019)
Data exclusions	No data was excluded.
Replication	Directed evolution experiment: The protocol for evolution of recombinases is reproducible (>300 evolutions were performed in the Buchholz Lab). The here described directed evolution for the target sites loxF8-L, loxF8-R and loxF8 was not replicated.<br>Activity assays of recombinases (PCR-based or plasmid based) were reproduced and successful.<br>Recombination assays in human cells were reproduced (n=3) and successful.<br>Genomic inversion and its quantification were reproduced (n=3) and successful.<br>Transfection of human iPSCs and ECs was reproduced (n=3) and successful.<br>qPCR-based quantification of the Factor VIII transcript was reproduced (n=3) and successful.
Randomization	Allocation was random.
Blinding	Bacterial and mammalian cell experiment were performed under the same conditions. No blinding was used in this study.

Reporting for specific materials, systems and methods

We require information from authors about some types of materials, experimental systems and methods used in many studies. Here, indicate whether each material, system or method listed is relevant to your study. If you are not sure if a list item applies to your research, read the appropriate section before selecting a response.

Materials & experimental systems

n/a	Involved in the study	
☐	☒	Antibodies
☐	☒	Eukaryotic cell lines
☒	☐	Palaeontology and archaeology
☒	☐	Animals and other organisms
☒	☐	Human research participants
☒	☐	Clinical data
☒	☐	Dual use research of concern

Methods

n/a	Involved in the study	
☐	☒	ChIP-seq
☐	☒	Flow cytometry
☒	☐	MRI-based neuroimaging

Antibodies

Antibodies used	anti-Factor VIII antibody (Abcam, ab236284), goat GFP-antibody (MPI-CBG antibody facility)
Validation	ab236284: Abcam validation: Paraffin-embedded human placental tissue stained for Factor VIII using ab236284, Paraffin-embedded human renal tissue stained for Factor VIII using ab236284, HeLa (human epithelial cell line from cervix adenocarcinoma) cells labeling Factor VIII using ab236284 (website: https://www.abcam.com/factor-viii-antibody-ab236284.html) Publications: Wang W et al. Comparative Transcriptional Analysis of Pulmonary Arterial Hypertension Associated With Three Different Diseases. Front Cell Dev Biol 9:672159 (2021). Liu J et al. Insulin-receptor substrate 1 protects against injury in endothelial cell models of ox-LDL-induced atherosclerosis by inhibiting ER stress/oxidative stress-mediated apoptosis and activating the Akt/FoxO1 signaling pathway. Int J Mol Med 46:1671-1682 (2020). Lou L et al. Lycium barbarum polysaccharide induced apoptosis and inhibited proliferation in infantile hemangioma endothelial cells via down-regulation of PI3K/AKT signaling pathway. Biosci Rep 39:N/A (2019). Zhao G et al. Clinical diagnosis of adult patients with acute megakaryocytic leukemia. Oncol Lett 16:6988-6997 (2018).<br>goat GFP-antibody: Chakraborty D, Paszkowski-Rogacz M, Berger N, Ding L, Mircetic J, Fu J, Iesmantavicius V, Choudhary C, Anastassiadis K, Stewart AF, Buchholz F. lncRNA Panct1 Maintains Mouse Embryonic Stem Cell Identity by Regulating TOBF1 Recruitment to Oct-Sox Sequences in Early G1. Cell Rep. 2017 Dec 12;21(11):3012-3021. doi: 10.1016/j.celrep.2017.11.045. PMID: 29241531.

Poser I, Sarov M, Hutchins JR, Hériché JK, Toyoda Y, Pozniakovsky A, Weigl D, Nitzsche A, Hegemann B, Bird AW, Pelletier L, Kittler R, Hua S, Naumann R, Augsburg M, Sykora MM, Hofemeister H, Zhang Y, Nasmyth K, White KP, Dietzel S, Mechtler K, Durbin R, Stewart AF, Peters JM, Buchholz F, Hyman AA. BAC TransgeneOmics: a high-throughput method for exploration of protein function in mammals. Nat Methods. 2008 May;5(5):409-15. doi: 10.1038/nmeth.1199. Epub 2008 Apr 6. Erratum in: Nat Methods. 2008 Aug;5 (8):748. PMID: 18391959; PMCID: PMC2871289.

Eukaryotic cell lines

Policy information about cell lines

Cell line source(s)	HEK293T - ATCC, HeLa TDS - MPI-CBG Dresden, human iPSCs - CRTD iPSCs facility (https://biotp.tu-dresden.de/facilities/stem-cell-engineering/services/)
Authentication	HEK293T and HeLa cells were not authenticated. The human iPSCs lines were authenticated (tested for pluripotency (FACS-based) and contamination of Mycoplasma) by the CRTD iPSCs facility.
Mycoplasma contamination	HEK293T and HeLa cells were not tested for Mycoplasma. Human iPSCs were tested negative for Mycoplasma.
Commonly misidentified lines (See ICLAC register)	No commonly misidentified cell lines were used in this study.

ChIP-seq

Data deposition

- [x] Confirm that both raw and final processed data have been deposited in a public database such as GEO.
- [x] Confirm that you have deposited or provided access to graph files (e.g. BED files) for the called peaks.

Data access links<br>*May remain private before publication.*	https://www.ncbi.nlm.nih.gov/geo/query/acc.cgi?acc=GSE159492<br>Reviewer access via a security token: gvojkwomrrcljyj
Files in database submission	Sequencing reads from RecF8-EGFP and EGFP ChIP-Seq samples (fastq format) and a list of called peaks (narrowPeak format)
Genome browser session (e.g. UCSC)	http://genome.ucsc.edu/s/Wo4CSn0Ftx/RecF8

Methodology

Replicates	No replicates
Sequencing depth	RecF8-EGFP: 35438250 paired-end reads (152 bp long), from which 29425544 (83%) were uniquely mapped<br>EGFP: 32721767 paired-end reads (152 bp long), from which 24908022 (76%) were uniquely mapped
Antibodies	goat anti-GFP (MPI-CBG antibody facility), anti-Factor VIII antibody (Abcam, ab236284), fluorescently labeled secondary antibody (Invitrogen, Alexa fluor 546 goat anti-rabbit)
Peak calling parameters	Read mapping: --chimSegmentMin 20 --alignIntronMax 1 --alignMatesGapMax 400 --outFilterMultimapNmax 50 --outFilterMismatchNmax 9999 --outFilterIntronStrands None<br>Index file generated from GENCODE primary assembly release 30<br>Peak calling: -m 30 -v -s 5 -x -r -e chrM,GL...,KI... -E hg38-blacklist.v2.bed.gz<br>ENCODE blacklist file downloaded from https://github.com/Boyle-Lab/Blacklist/raw/master/lists/mm10-blacklist.v2.bed.gz
Data quality	85 peaks identified with default cut-offs: a maximum p-value of 0.01 and a minimum AUC of 200.
Software	Read mapping: STAR (https://doi.org/10.1093/bioinformatics/bts635)<br>Peak calling: https://github.com/jsh58/Genrich

Flow Cytometry

Plots

Confirm that:

- [x] The axis labels state the marker and fluorochrome used (e.g. CD4-FITC).
- [x] The axis scales are clearly visible. Include numbers along axes only for bottom left plot of group (a 'group' is an analysis of identical markers).
- [x] All plots are contour plots with outliers or pseudocolor plots.
- [x] A numerical value for number of cells or percentage (with statistics) is provided.

Methodology

Sample preparation	HEK293T cells and HeLa cells were washed once with PBS and then detached using Trypsin for 2 min. Afterwards cells were collected in FACS buffer (PBS with 2.5mM EDTA and 1% BSA) for analysis. hIPSCs were washed once with PBS and then detached using Accutase for 1 min. Cells were collected in FACS buffer (PBS with 2.5mM EDTA and 1% BSA) for analysis.
Instrument	MACSQuant® VYB Flow Cytometer, BD FACSCanto™ II Cell Analyzer
Software	FlowJo™ 10
Cell population abundance	Abundance of relevant cells within the post-sort fraction: HEK293T BFP+ 30-80%, HEK293T mCherry+ 10-80%, HeLa EGFP+ 10-25%, hIPSCs EGFP+ 90-98%. Since all the samples were derived from a culture system with only one cell type, the purity was not further determined.
Gating strategy	HEK293T reporter cell line basal mCherry gating strategy: starting population FSC-A 10^1-10^2.5, SSC-A 10^1.3-10^2.4, mCherry positive dsTexas_red-A > 10^0 (1)<br>HEK293T reporter cell line transfection efficiency gating strategy: starting population FCS-A 200-900 and SSC-A 50-950, tagBFP positive VioBlue-H > 10^-0.3<br>HEK293T reporter cell line recombination gating strategy: starting population FCS-A 200-900 and SSC-A 50-950, tagBFP and mCherry positive VioBlue-A > 10^-1 and dsTexas_red-A > 10^-1

☒ Tick this box to confirm that a figure exemplifying the gating strategy is provided in the Supplementary Information.
