# Supplementary material for: Correction of a Factor VIII genomic inversion with designer-recombinases
Source: Nat Commun. 2022 Jan 20;13:422. doi: 10.1038/s41467-022-28080-7 (PMC8776779; doi:10.1038/s41467-022-28080-7)
Supplement: Supplementary file 4 — Description of Additional Supplementary Files [file 41467_2022_28080_MOESM4_ESM.pdf]

Title: Supplementary Data 1:
Description: 82 potential recombinase target sites (34 bp) found in the int1 repeat surrounding the F8 gene. Genomic coordinates are indicated. The target sites are ranked by the number of mismatches (asymmetry) between the left and right half sites of a given spacer. The target sites are then compared to target sites of previous evolutions and the maximum mismatch count is displayed. The first sequence of this list has the lowest asymmetry and the lowest maximum mismatch count and was used in this study to evolve recombinases. It is called loxF8 (highlighted in yellow).

Title: Supplementary Data 2:
Description: Potential off-targets with similarity towards loxF8, loxF8-L and loxF8-R. The chromosomal coordinates and sequence (left half-site, right half-site and spacer) of each identified site are displayed. For each half-site of a potential off-target site the mismatches are displayed separately. The total mismatches indicate the similarity of the identified site compared to the full loxF8, loxF8-L or loxF8-R. A maximum of 7 mismatches was allowed to identify potential off-targets.

Title: Supplementary Data 3:
Description: Putative binding sites identified by recombinase ChIP-seq. 85 potential binding sites are shown with their genomic coordinates and the summit of each peak is displayed. Yellow marked lines indicate putative binding sites used for experimental investigation.

Title: Supplementary Data 4:
Description: Nucleotide sequences of primers used in this study. Top strand primers are marked with a '_F' and bottom strand primers are marked with a '_R'. A small description of use of the primer is shown.
