# Supplementary figures and images for: Correction of a Factor VIII genomic inversion with designer-recombinases
Source: Nat Commun. 2022 Jan 20;13:422. doi: 10.1038/s41467-022-28080-7 (PMC8776779; doi:10.1038/s41467-022-28080-7)

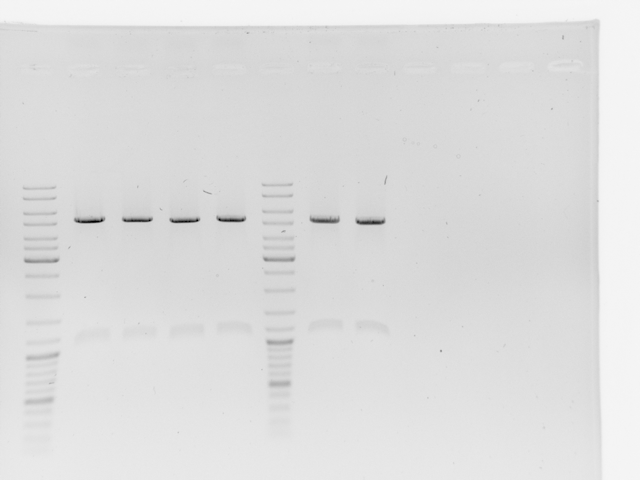

Supplement: Supplementary file 9 — Source data [file 41467_2022_28080_MOESM9_ESM.zip › Source Data/Figure 4d/Figure 4d - L8 symmetric off-targets.tiff]

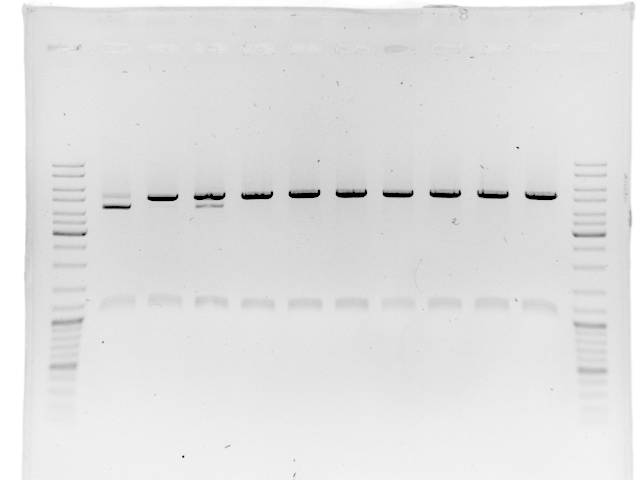

Supplement: Supplementary file 9 — Source data [file 41467_2022_28080_MOESM9_ESM.zip › Source Data/Figure 4d/Figure 4d - L8 asymmetric off-targets.tiff]

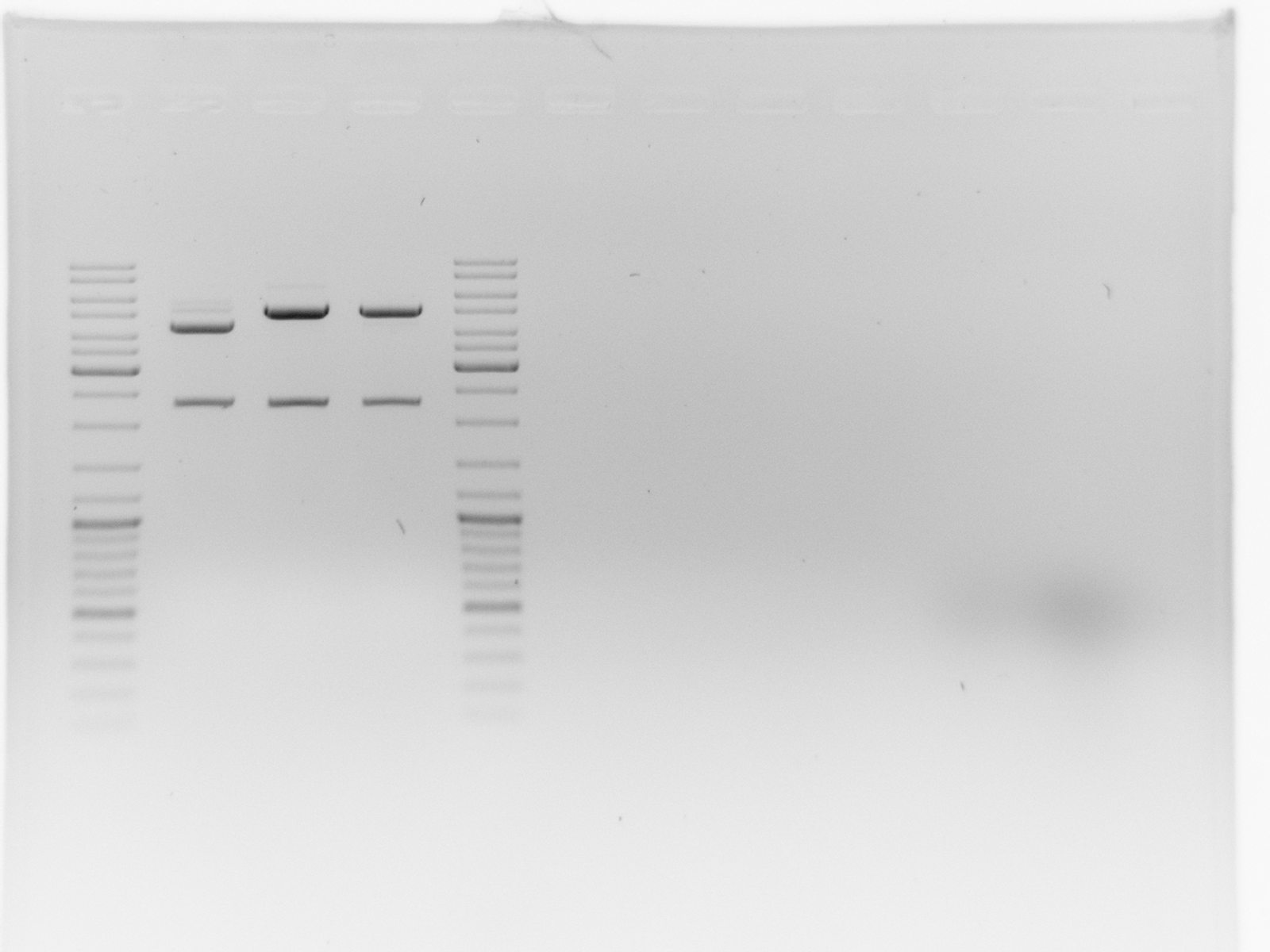

Supplement: Supplementary file 9 — Source data [file 41467_2022_28080_MOESM9_ESM.zip › Source Data/Supp. Figure 13c/Supp. Figure 13c - RecF8 on loxF8, Sym1, Sym2, at ara200 wh.Tif]

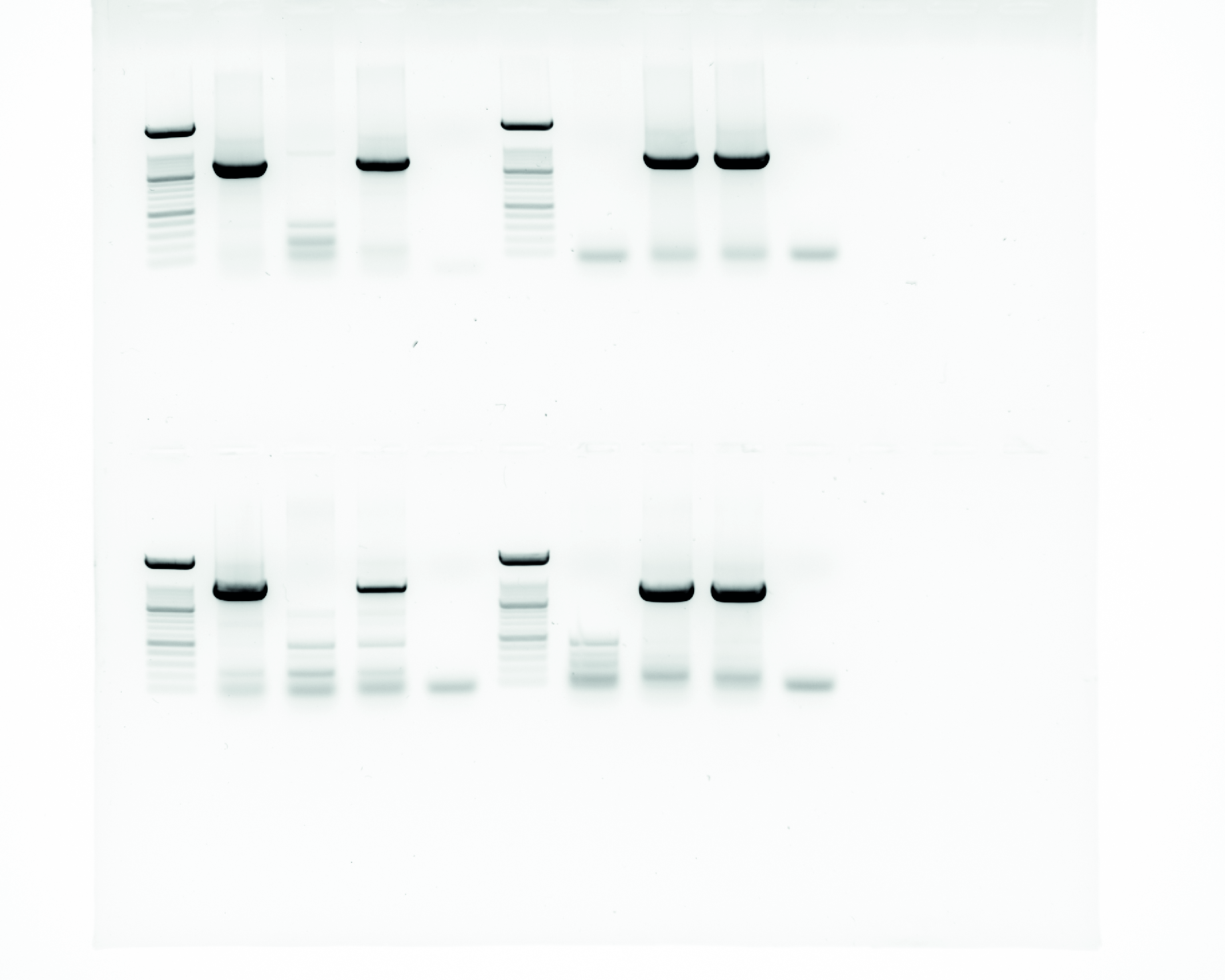

Supplement: Supplementary file 9 — Source data [file 41467_2022_28080_MOESM9_ESM.zip › Source Data/Supp. Figure 19c/Figure 19c - Inversion PCR on EC gDNA right part of the gel.tif]

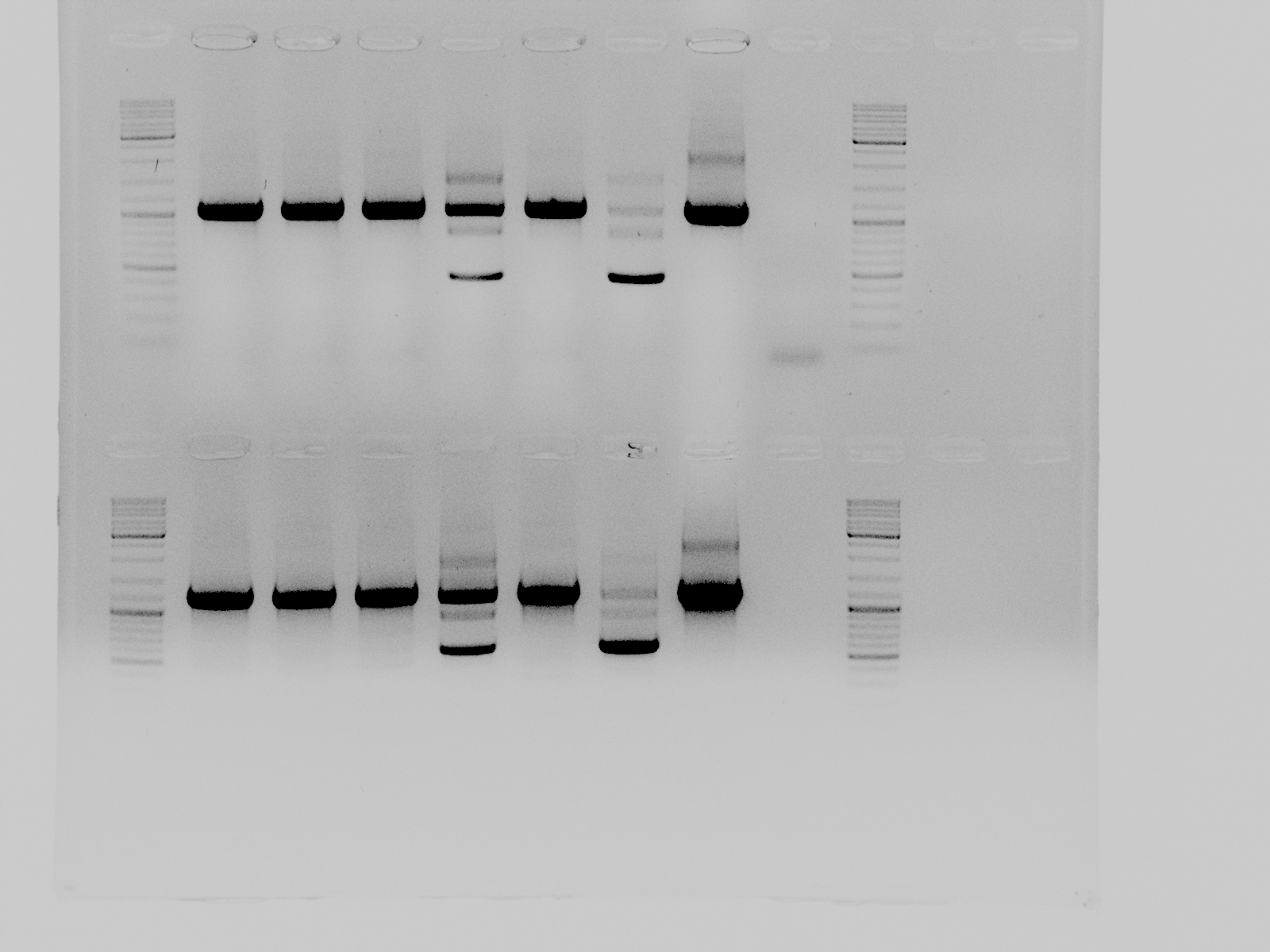

Supplement: Supplementary file 9 — Source data [file 41467_2022_28080_MOESM9_ESM.zip › Source Data/Supp. Figure 10c/Supp. Figure 10c - PCR on genomic DNA of reporter cell lines.Tif]

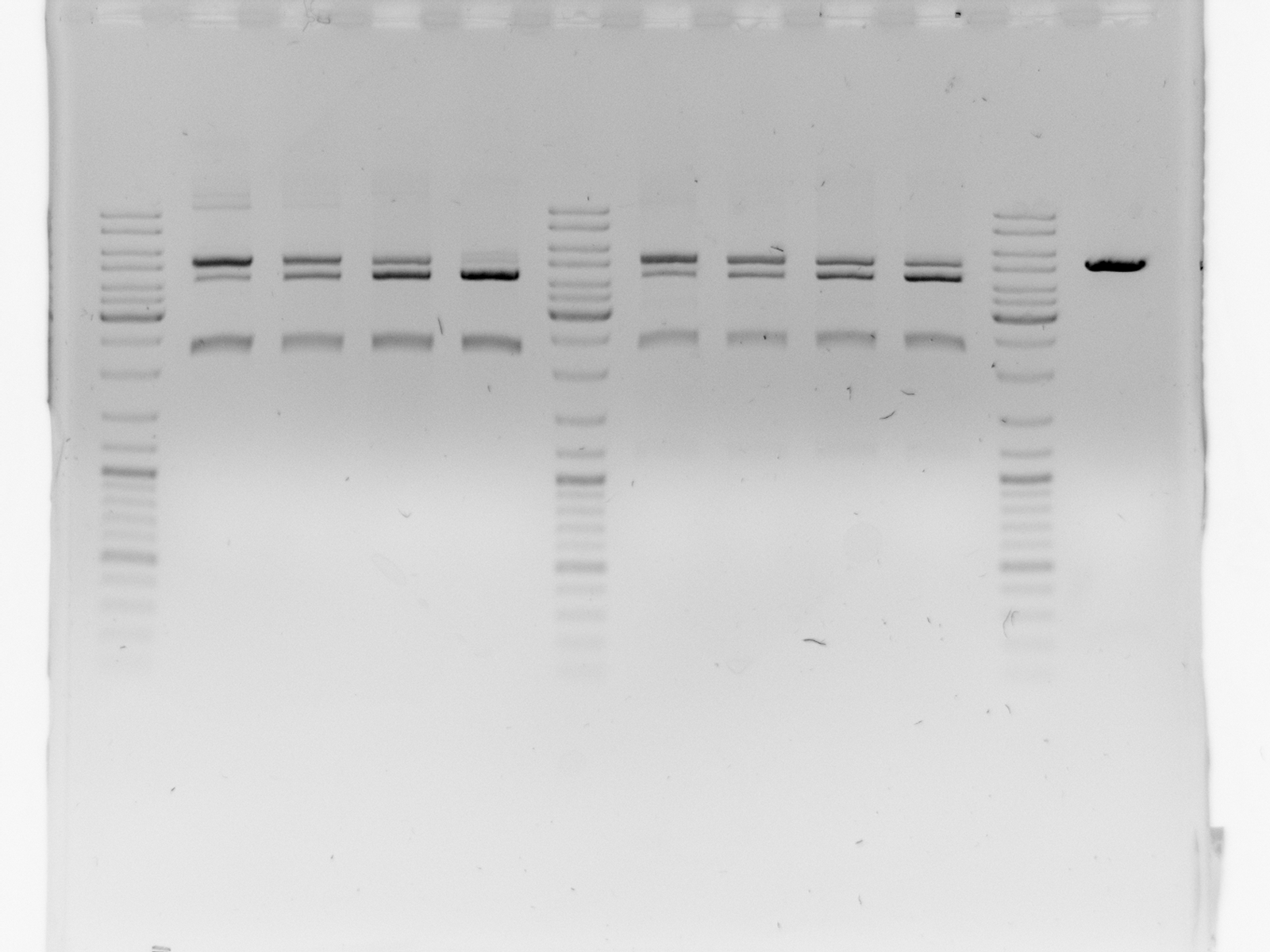

Supplement: Supplementary file 9 — Source data [file 41467_2022_28080_MOESM9_ESM.zip › Source Data/Supp. Figure 13b/Supp. Figure 13b - RecF8 activity on loxF8 right part of the gel.tif]

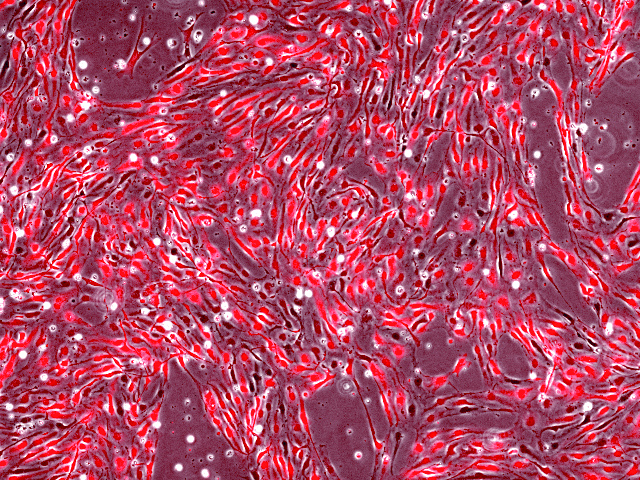

Supplement: Supplementary file 9 — Source data [file 41467_2022_28080_MOESM9_ESM.zip › Source Data/Supp. Figure 19b/Supp. Figure 19b - EC merge.tif]

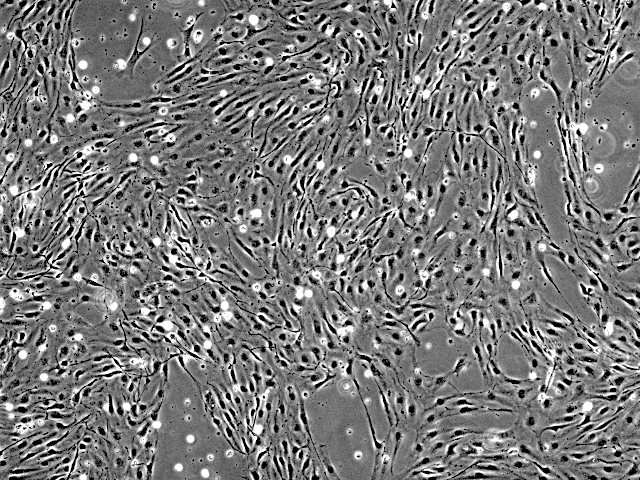

Supplement: Supplementary file 9 — Source data [file 41467_2022_28080_MOESM9_ESM.zip › Source Data/Supp. Figure 19b/Supp. Figure 19b - EC BF 10x.tif]

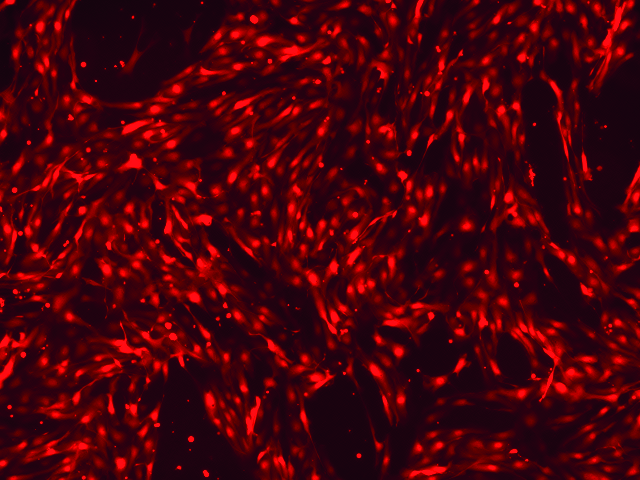

Supplement: Supplementary file 9 — Source data [file 41467_2022_28080_MOESM9_ESM.zip › Source Data/Supp. Figure 19b/Supp. Figure 19b - EC mCherry.tif]

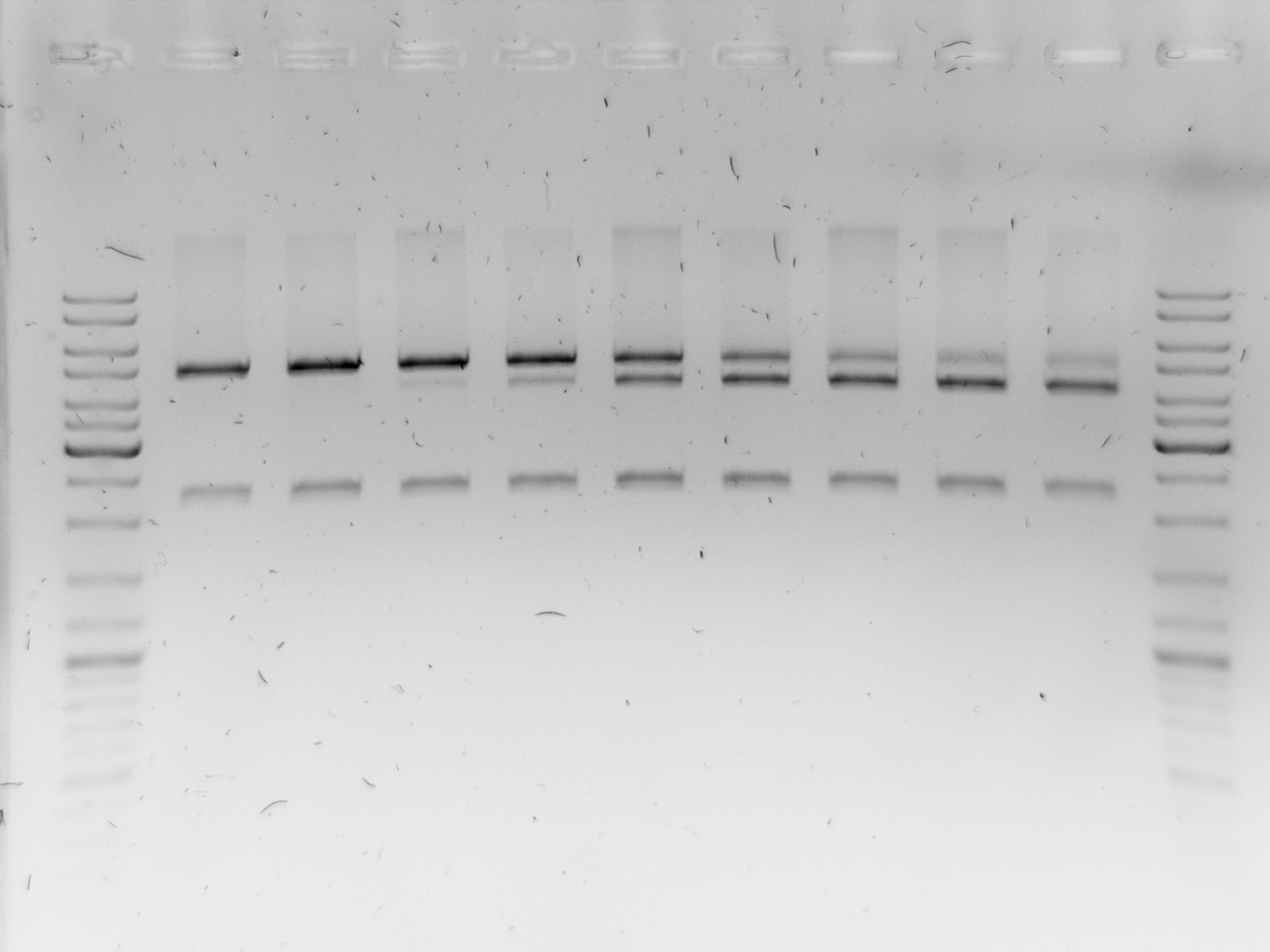

Supplement: Supplementary file 9 — Source data [file 41467_2022_28080_MOESM9_ESM.zip › Source Data/Supp. Figure 12a/Supp. Figure 12a - linked dimer on loxF8 Ara 0,1,5,10,25,50,100,200,250.tif]

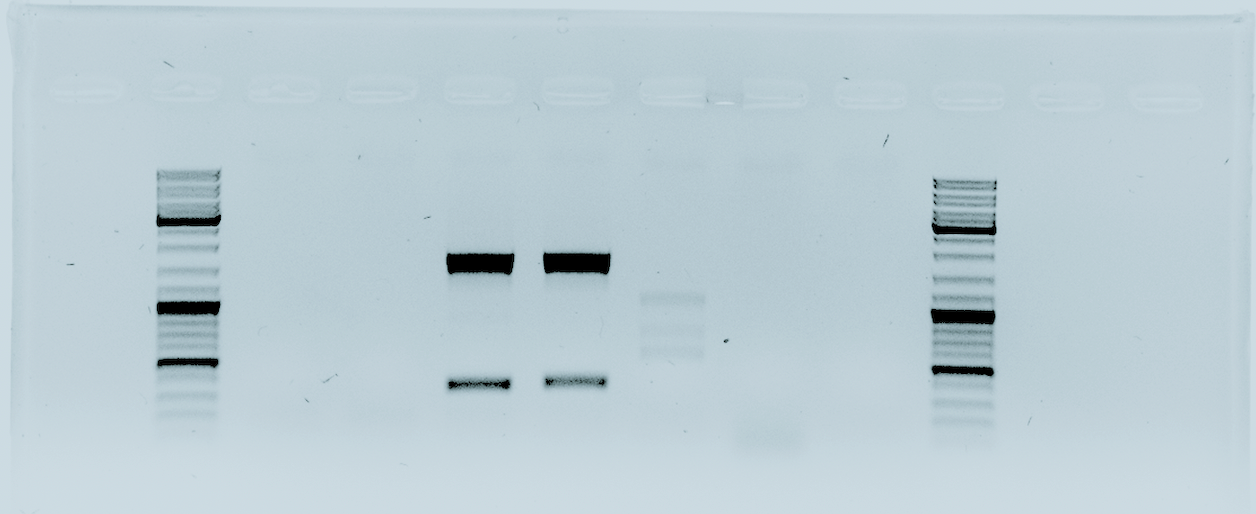

Supplement: Supplementary file 9 — Source data [file 41467_2022_28080_MOESM9_ESM.zip › Source Data/Supp. Figure 17c/Supp. Figure 17c - PCR on gDNA to test off-target recombination.tif]

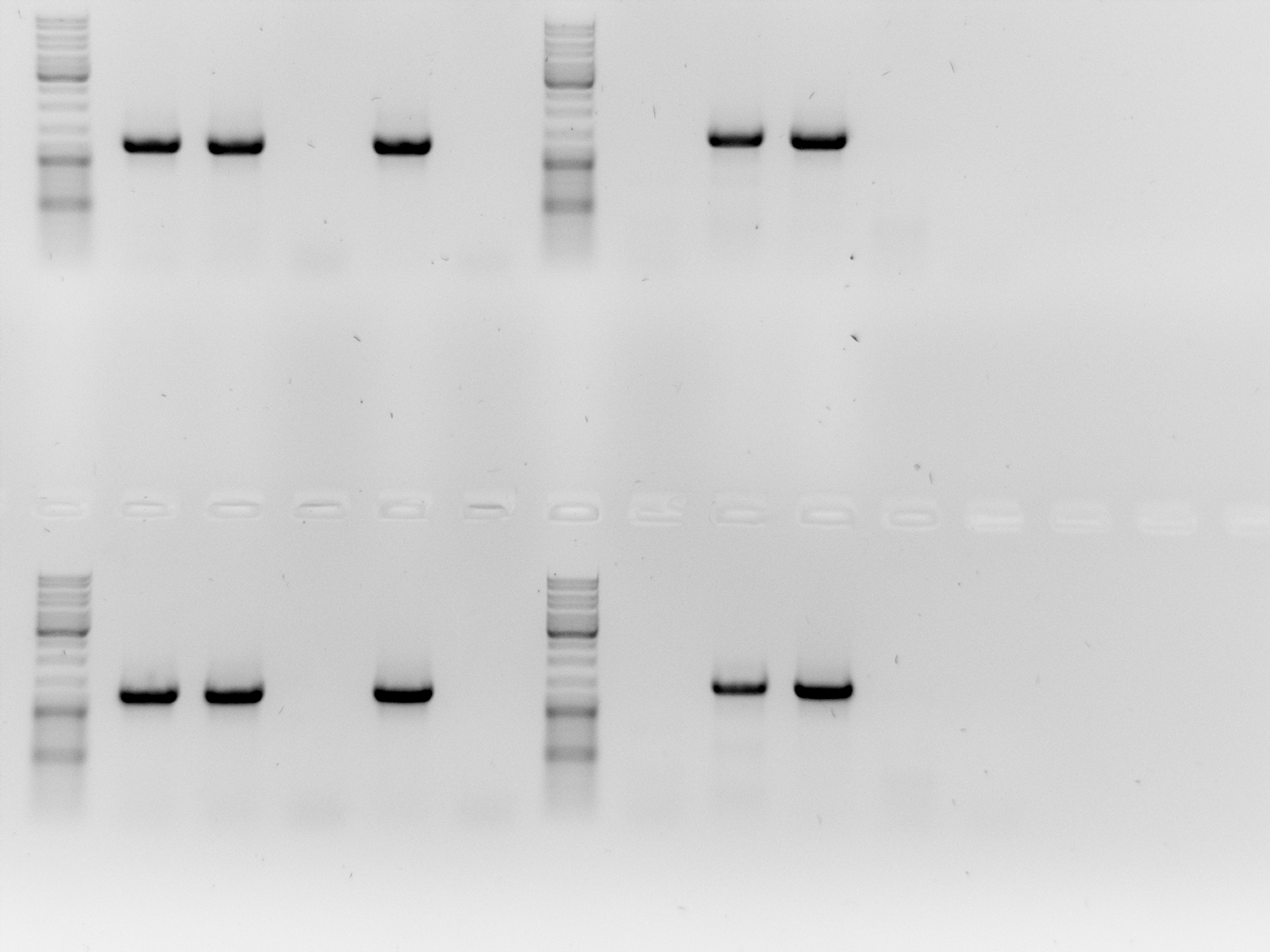

Supplement: Supplementary file 9 — Source data [file 41467_2022_28080_MOESM9_ESM.zip › Source Data/Figure 3e/Figure 3e - D7 mediated genomic inversion.Tif]

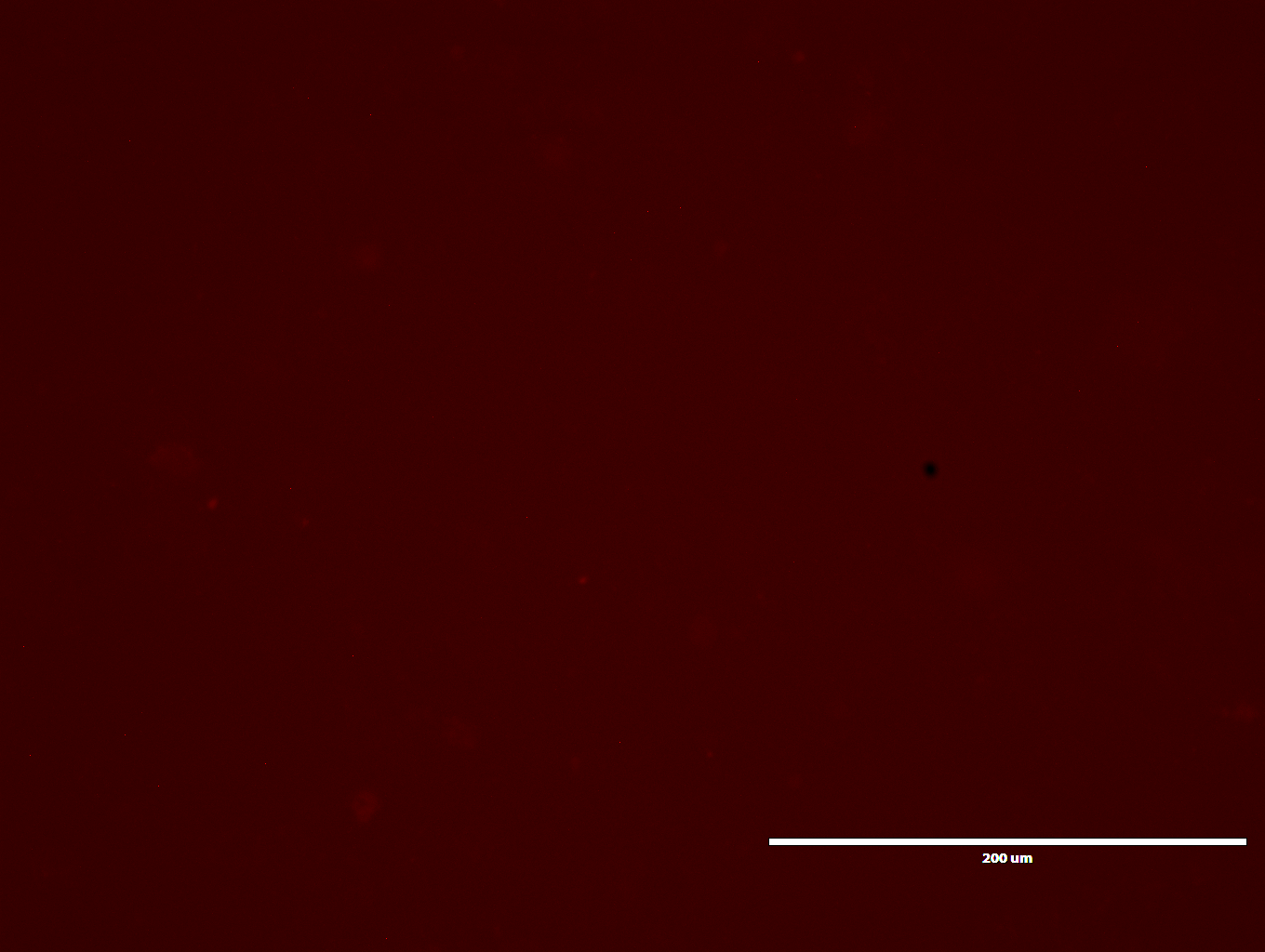

Supplement: Supplementary file 9 — Source data [file 41467_2022_28080_MOESM9_ESM.zip › Source Data/Figure 3b/D7left-loxF8rep-mCh.tif]

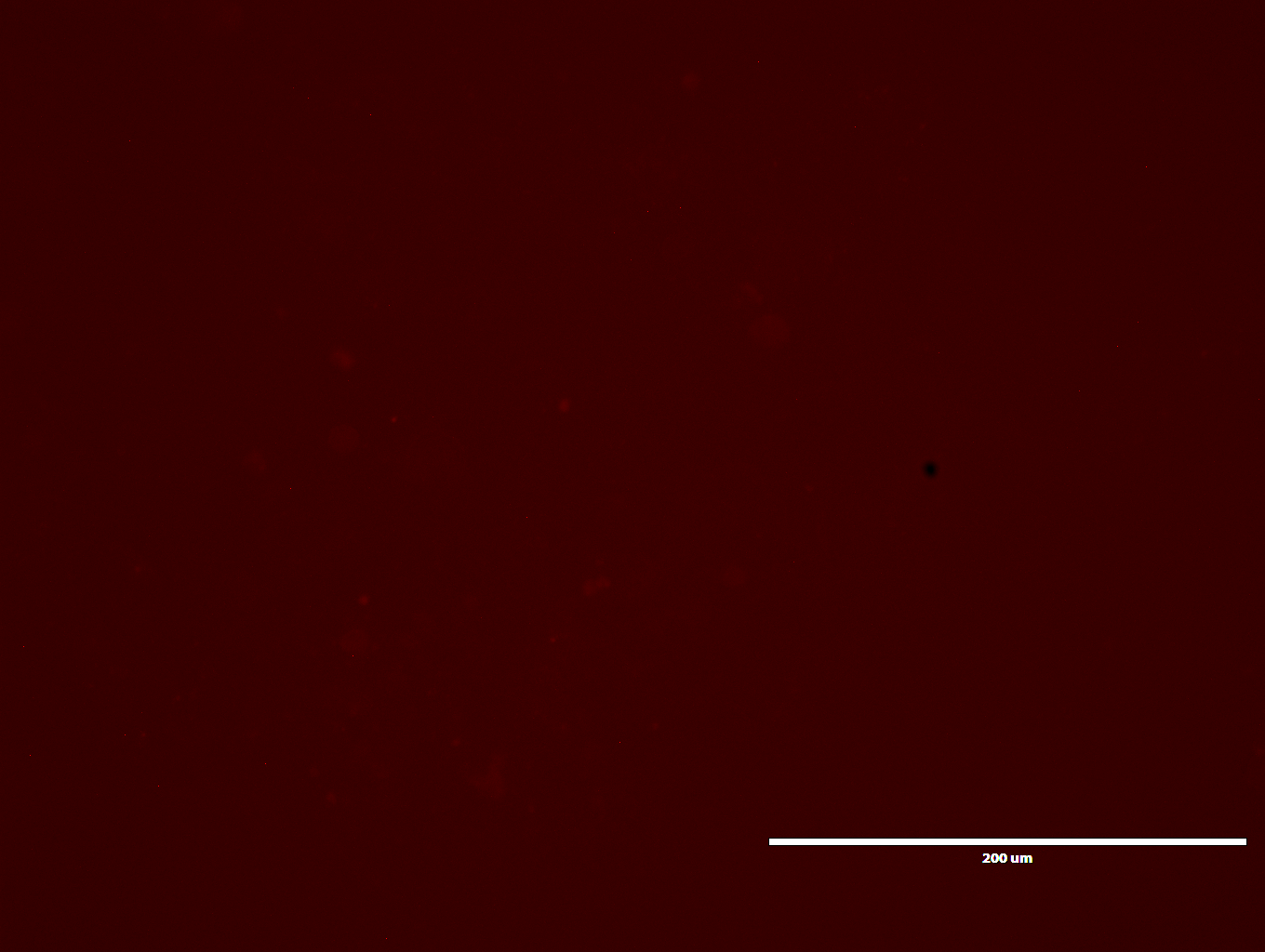

Supplement: Supplementary file 9 — Source data [file 41467_2022_28080_MOESM9_ESM.zip › Source Data/Figure 3b/D7rght-loxF8rep-mCh.tif]

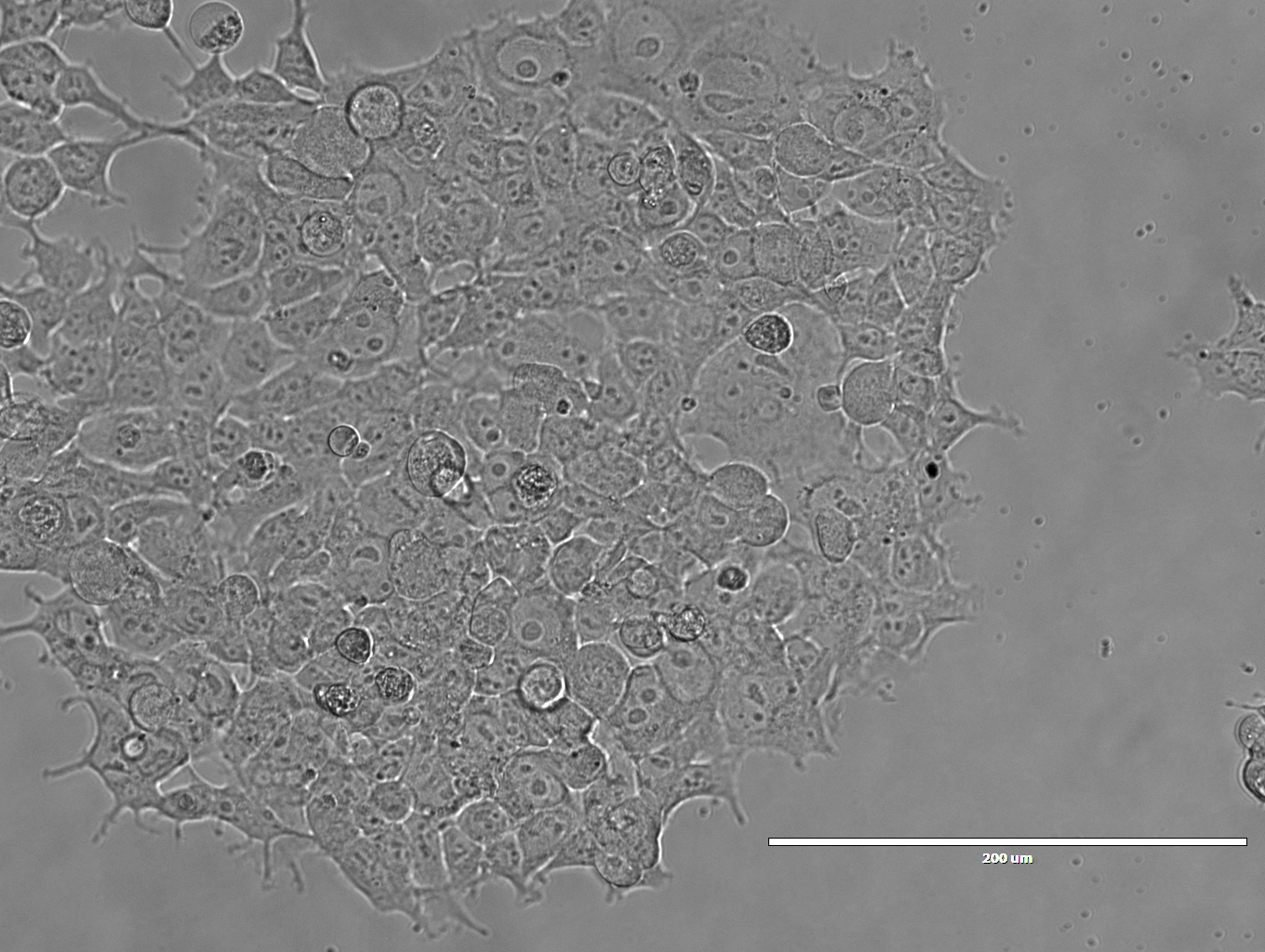

Supplement: Supplementary file 9 — Source data [file 41467_2022_28080_MOESM9_ESM.zip › Source Data/Figure 3b/D7rght-loxF8rep-BF.tif]

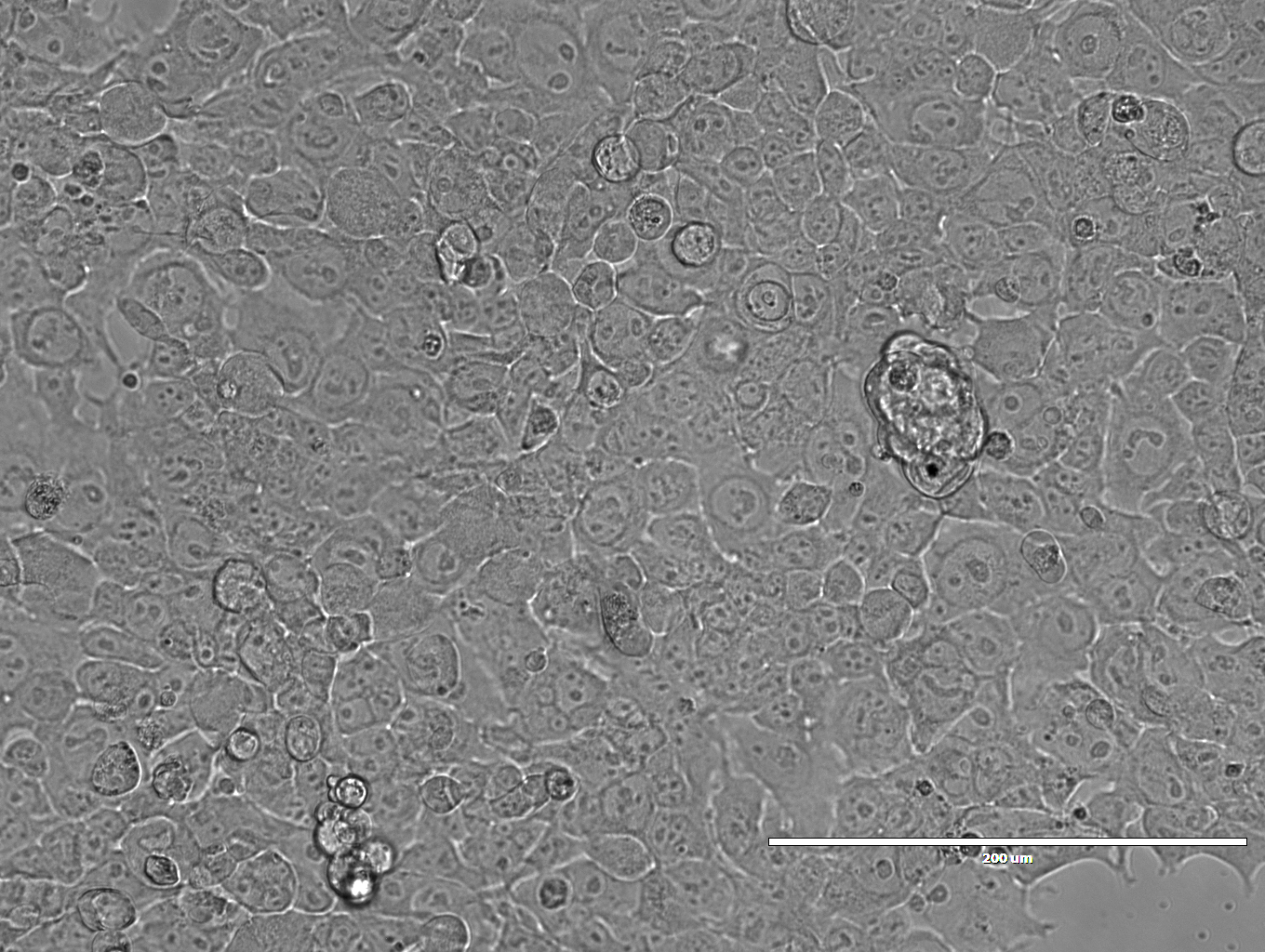

Supplement: Supplementary file 9 — Source data [file 41467_2022_28080_MOESM9_ESM.zip › Source Data/Figure 3b/D7-loxF8rep-BF.tif]

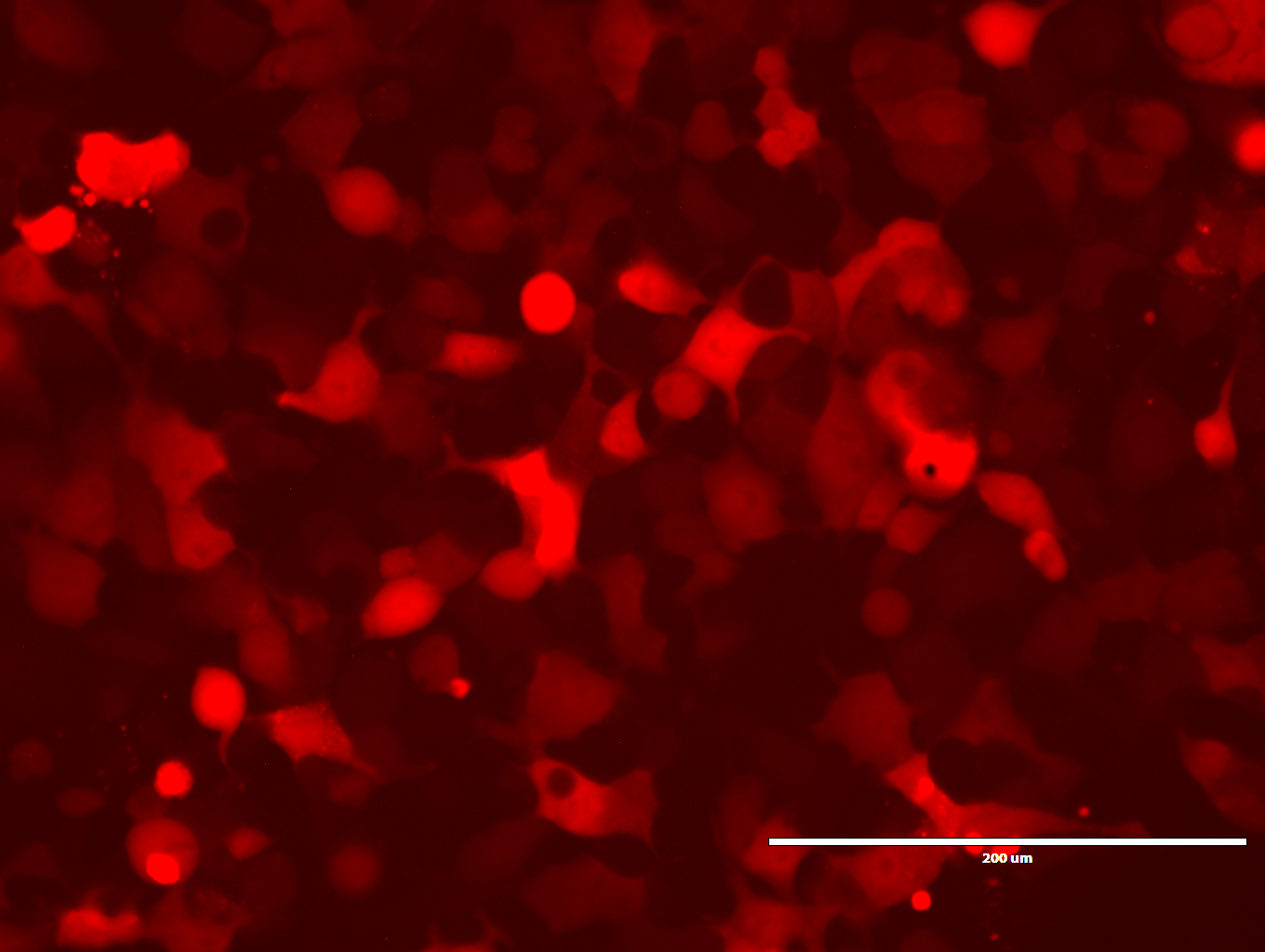

Supplement: Supplementary file 9 — Source data [file 41467_2022_28080_MOESM9_ESM.zip › Source Data/Figure 3b/D7-loxF8rep-mCh.tif]

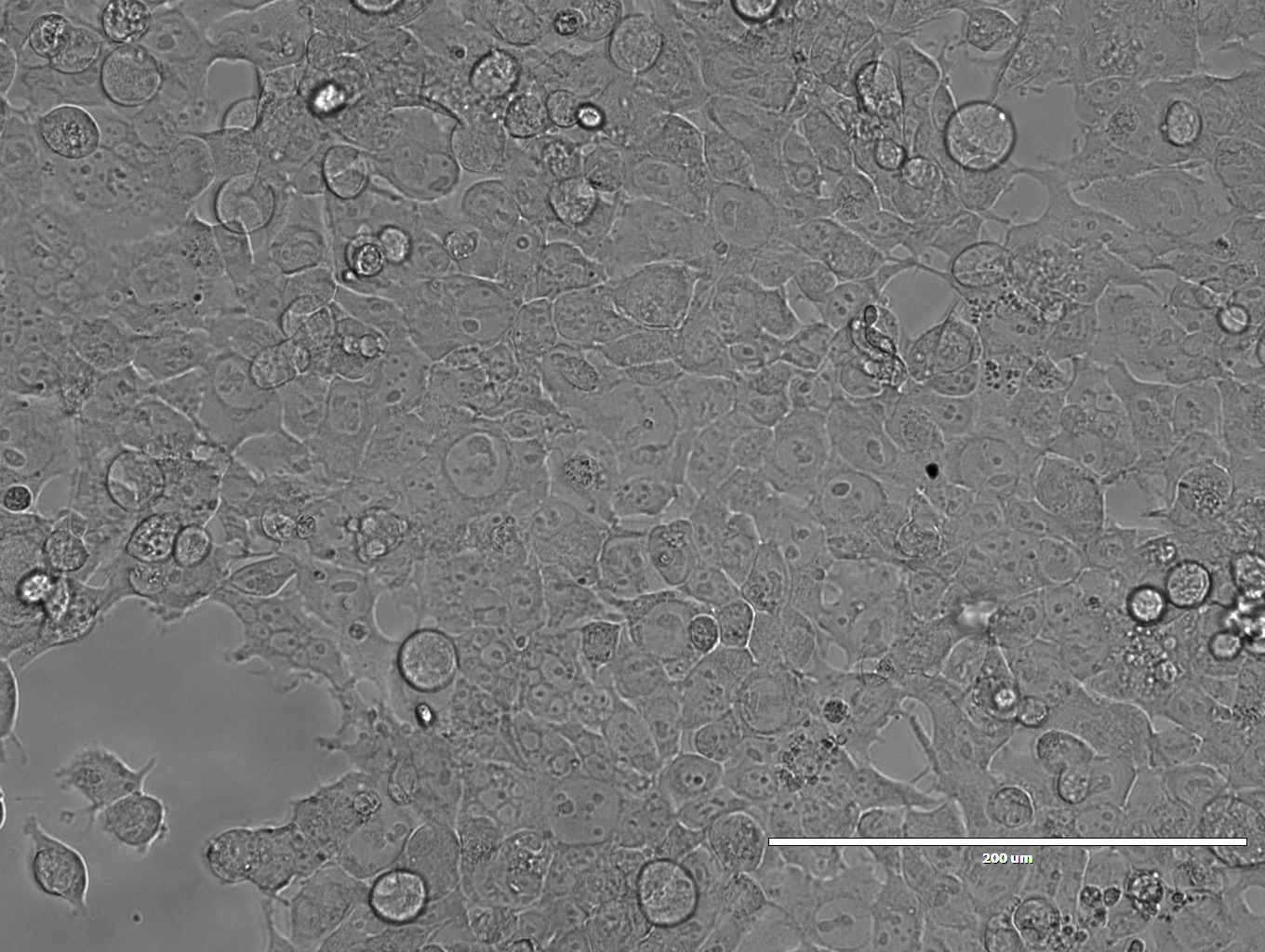

Supplement: Supplementary file 9 — Source data [file 41467_2022_28080_MOESM9_ESM.zip › Source Data/Figure 3b/D7left-loxF8rep-BF.tif]

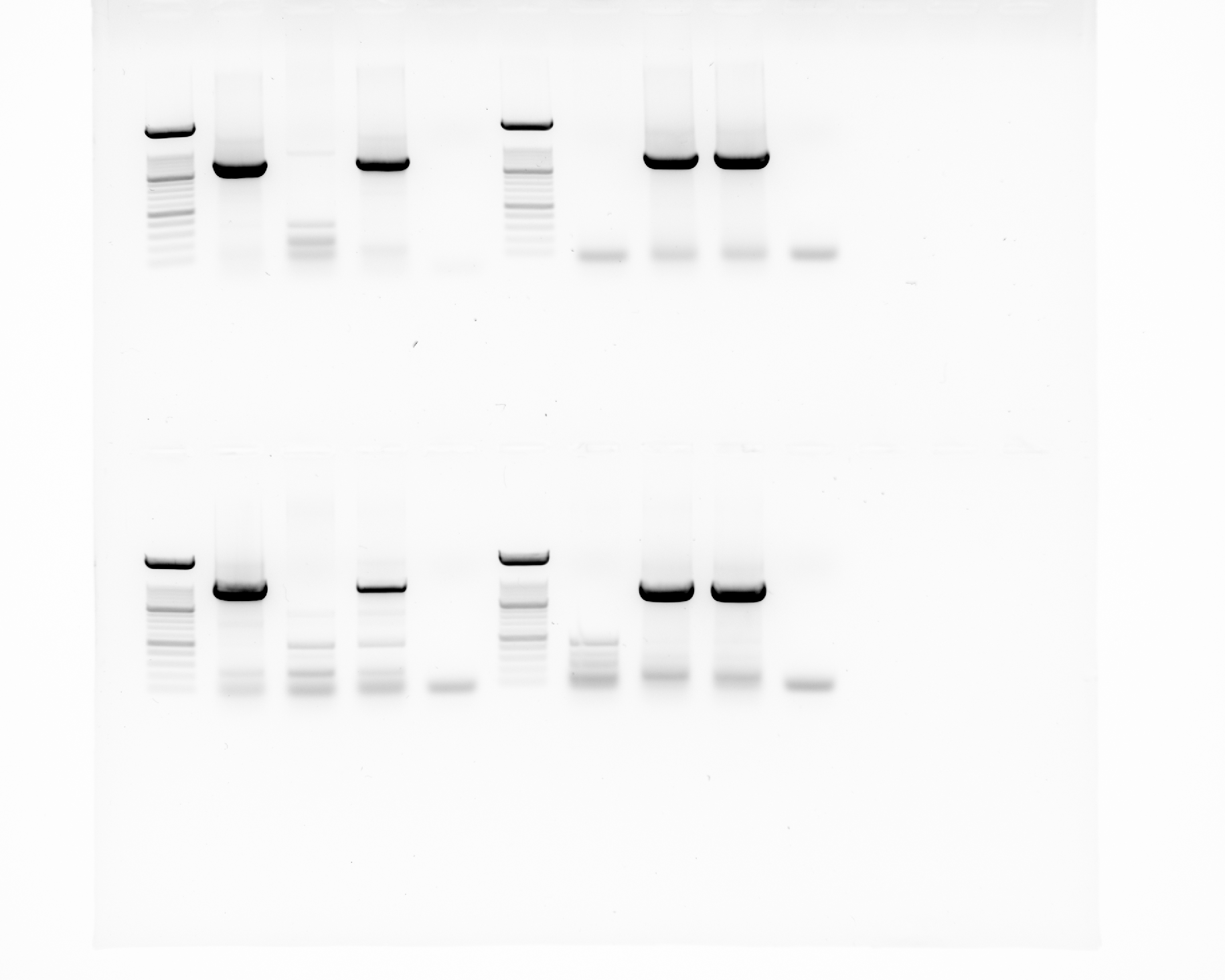

Supplement: Supplementary file 9 — Source data [file 41467_2022_28080_MOESM9_ESM.zip › Source Data/Figure 6a/Figure 6a - Inversion PCR on EC gDNA left part of the gel.tif]

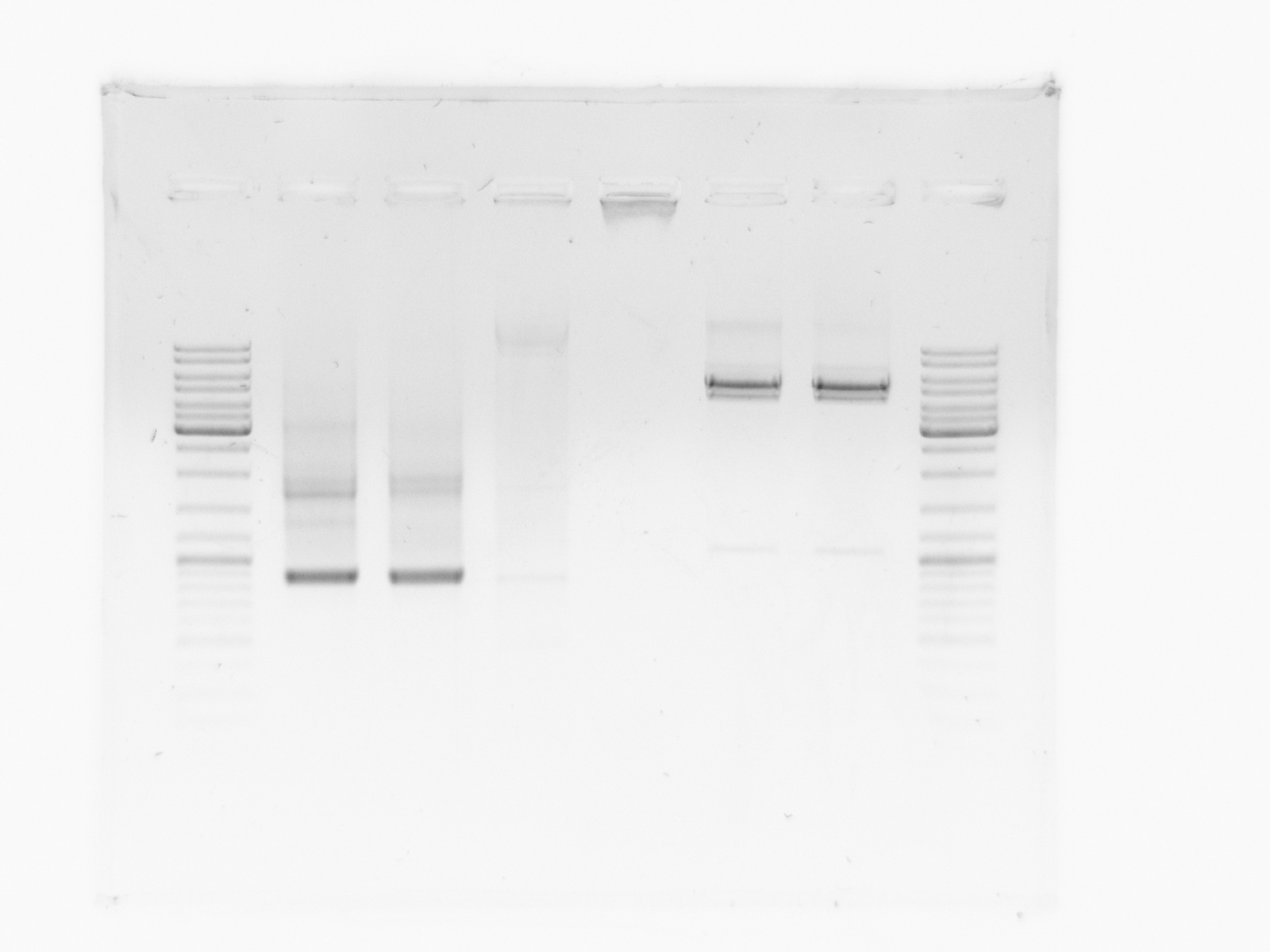

Supplement: Supplementary file 9 — Source data [file 41467_2022_28080_MOESM9_ESM.zip › Source Data/Supp. Figure 3b/20170512 - sym1 and sym2 cy1.Tif]

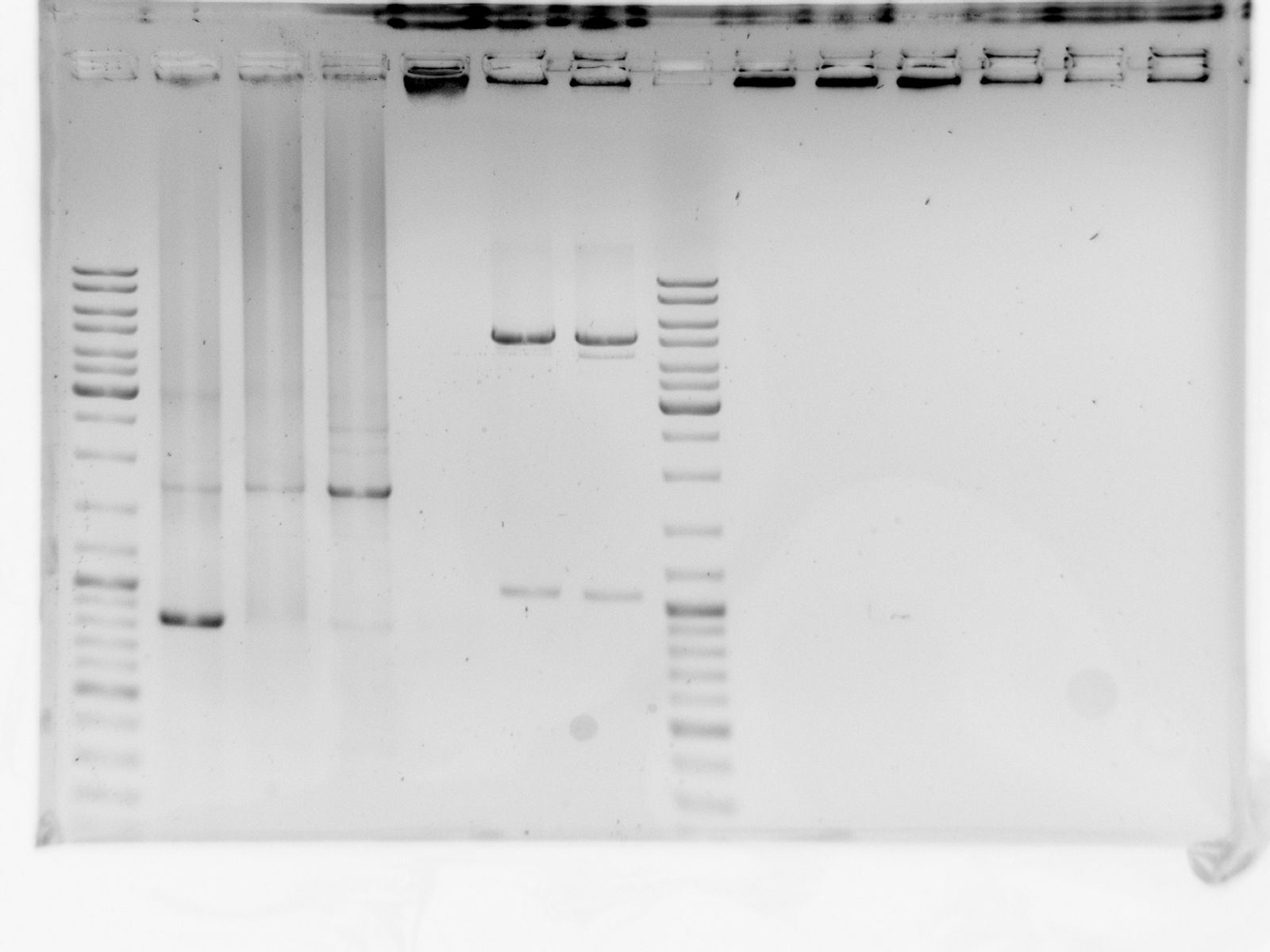

Supplement: Supplementary file 9 — Source data [file 41467_2022_28080_MOESM9_ESM.zip › Source Data/Supp. Figure 3b/20160711 - sym1b and sym2b cy1.Tif]

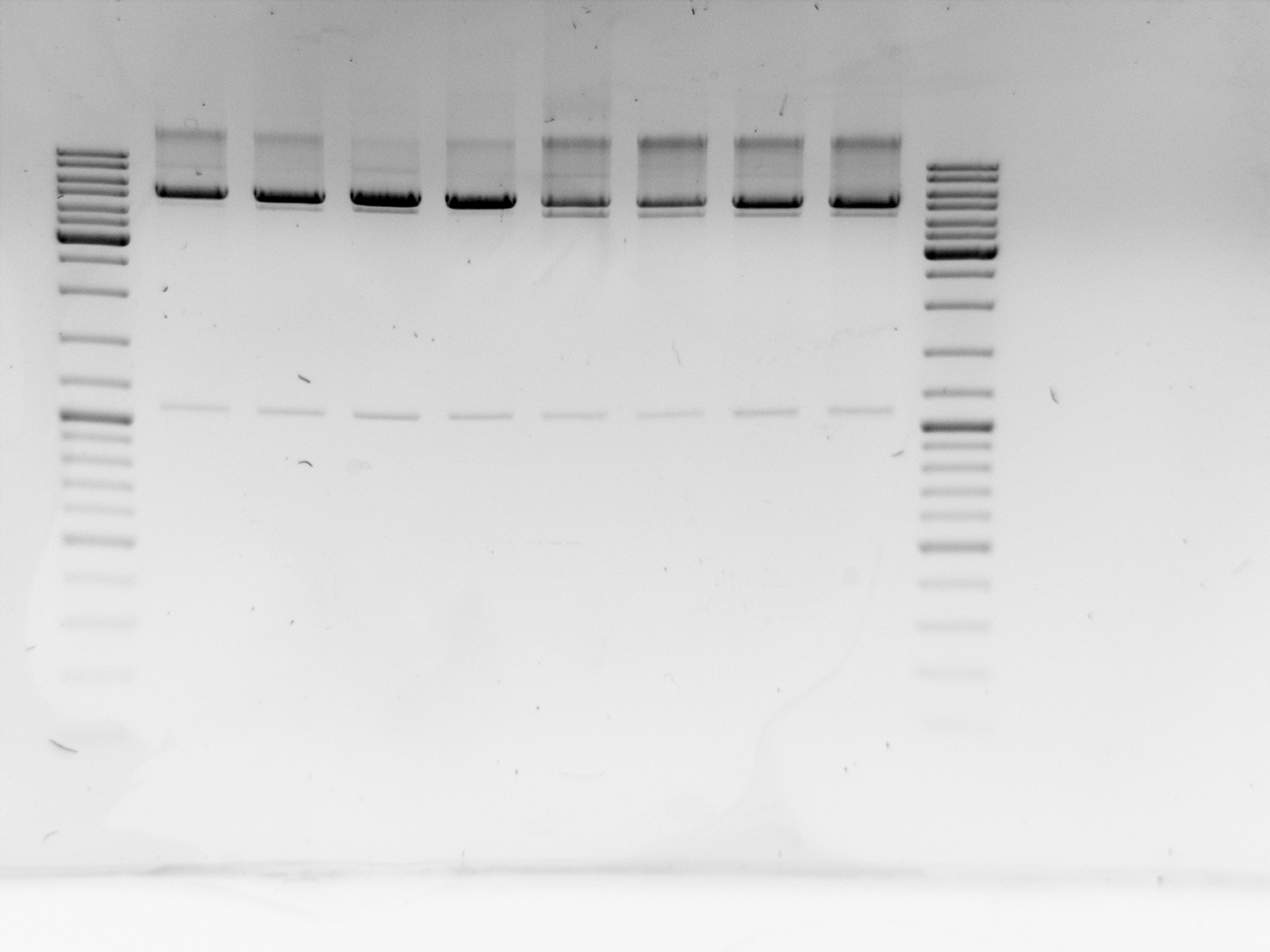

Supplement: Supplementary file 9 — Source data [file 41467_2022_28080_MOESM9_ESM.zip › Source Data/Supp. Figure 3b/20161211 - sym1c and sym2c cy13.Tif]

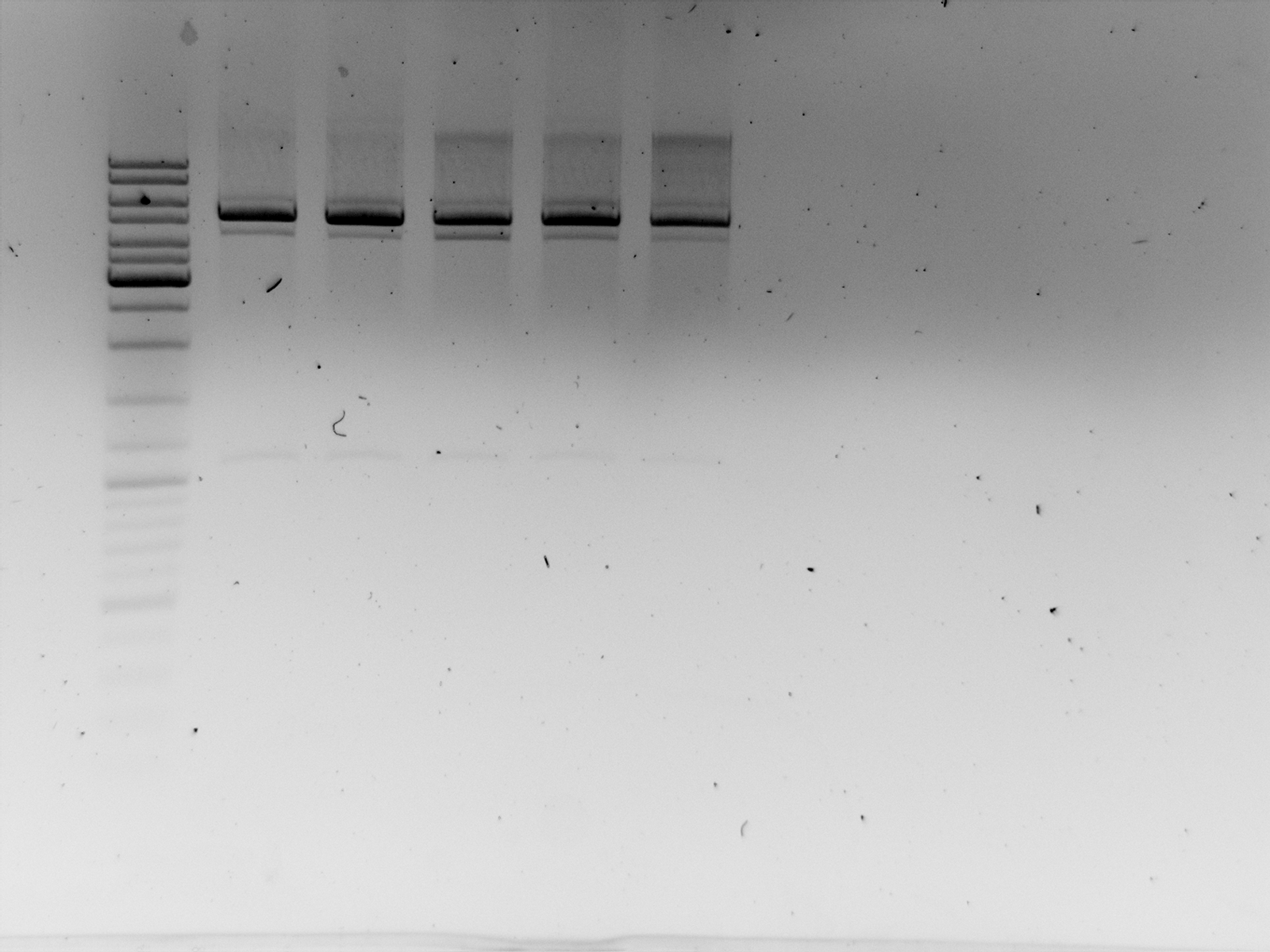

Supplement: Supplementary file 9 — Source data [file 41467_2022_28080_MOESM9_ESM.zip › Source Data/Supp. Figure 3b/20180315 - sym1 and sym2 cy30-31.Tif]

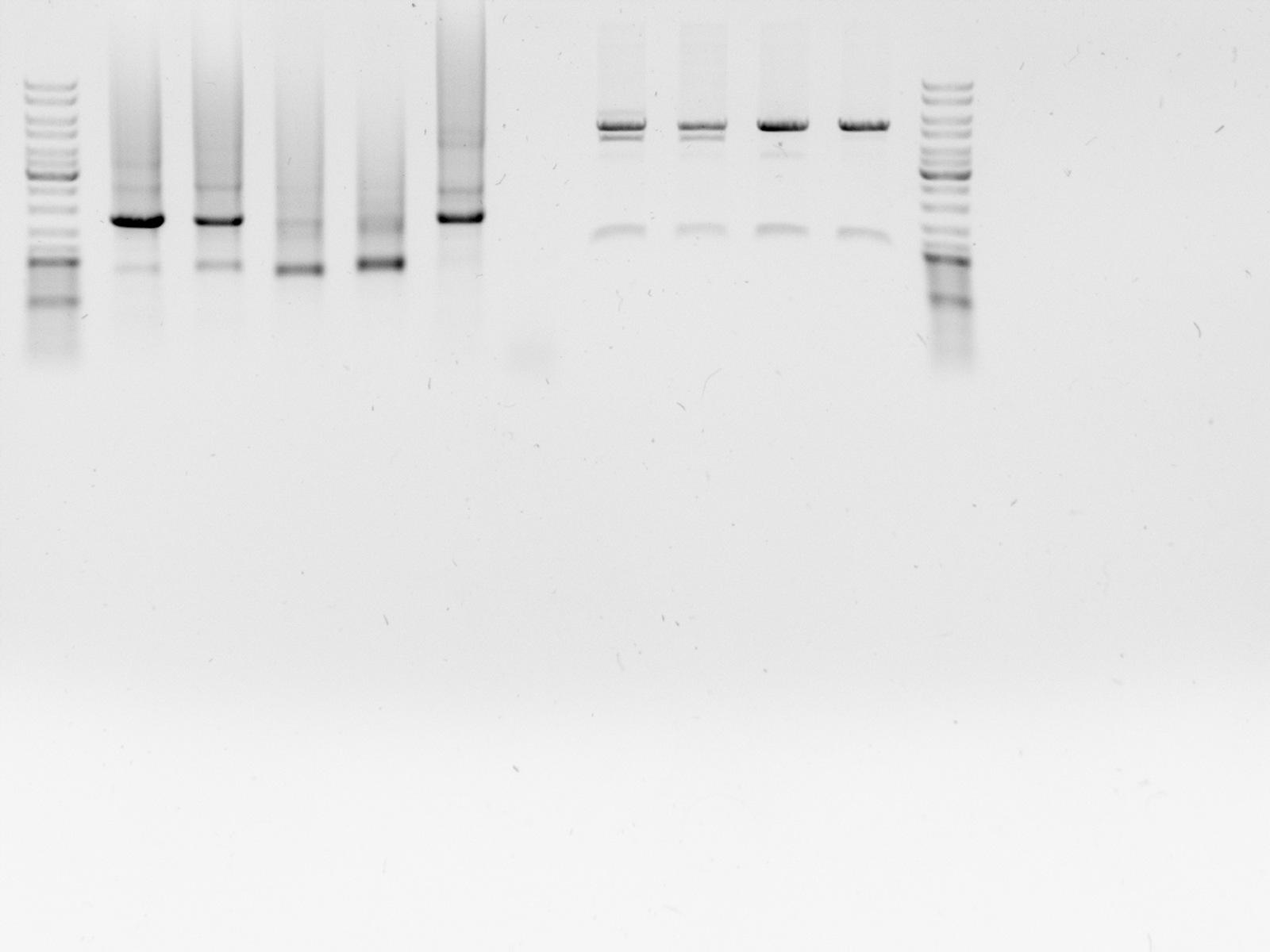

Supplement: Supplementary file 9 — Source data [file 41467_2022_28080_MOESM9_ESM.zip › Source Data/Supp. Figure 3b/20160923 - sym1b and sym2b cy14.Tif]

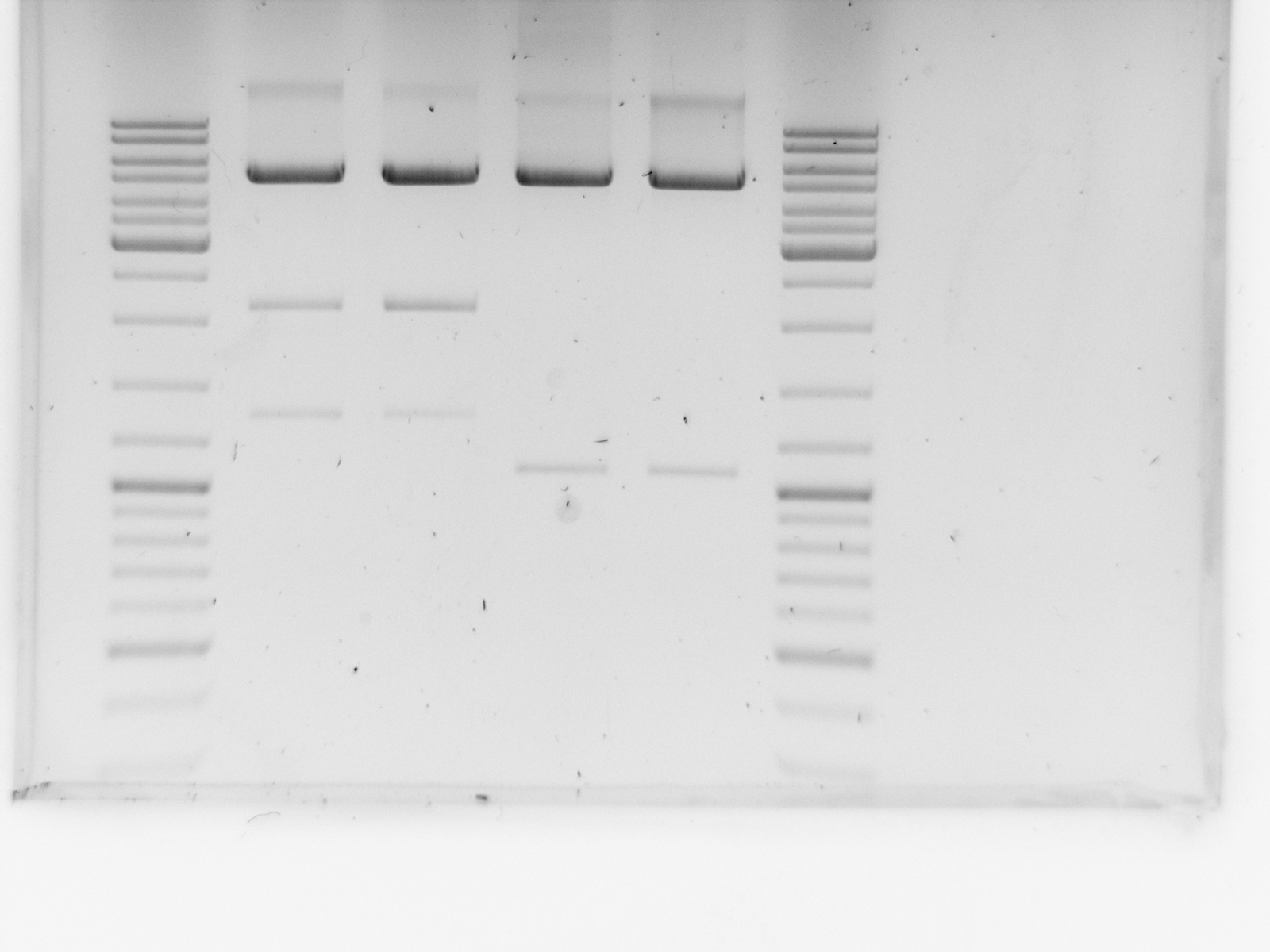

Supplement: Supplementary file 9 — Source data [file 41467_2022_28080_MOESM9_ESM.zip › Source Data/Supp. Figure 3b/20161024 - sym1c and sym2c cy1.Tif]

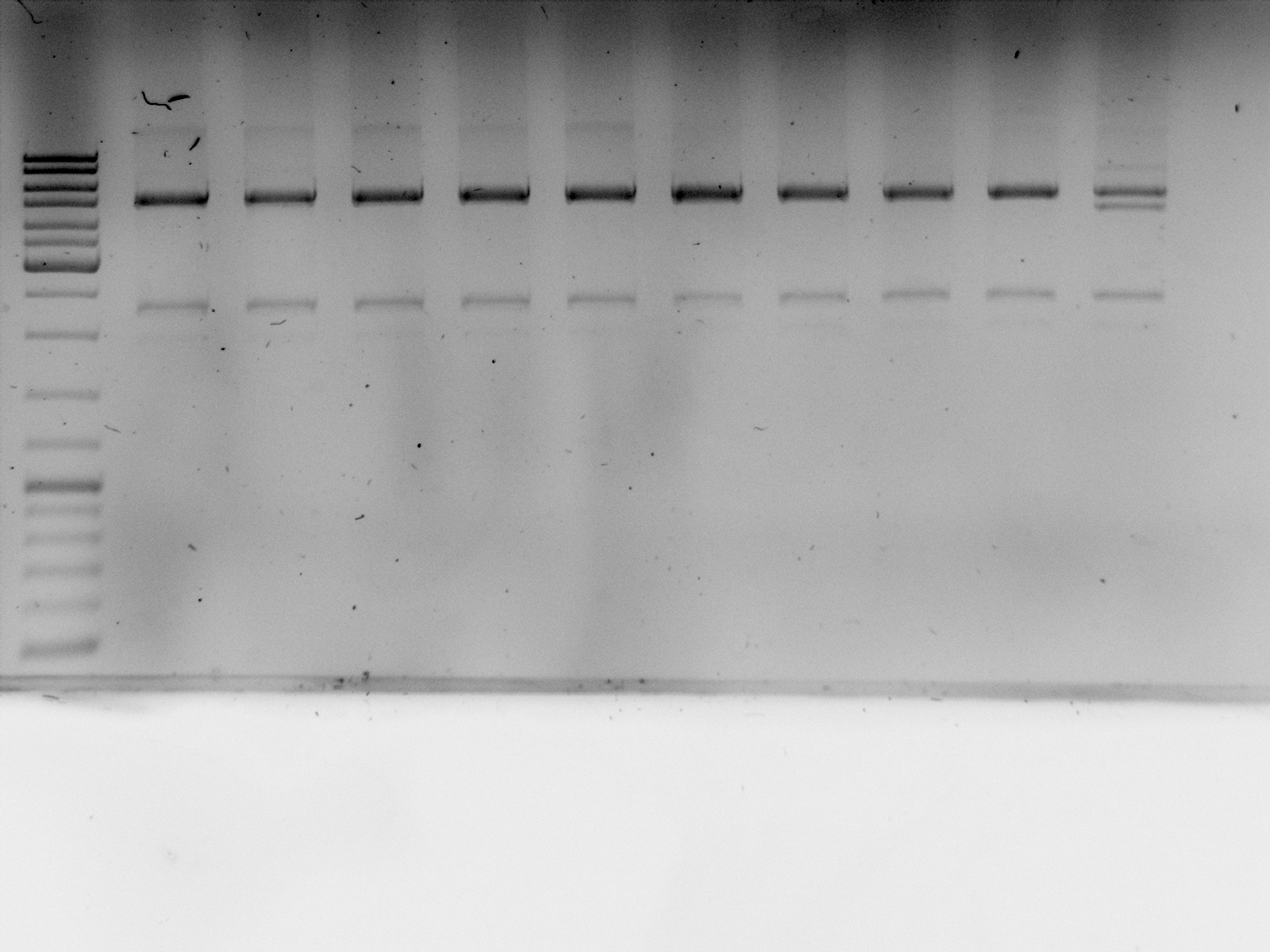

Supplement: Supplementary file 9 — Source data [file 41467_2022_28080_MOESM9_ESM.zip › Source Data/Supp. Figure 3b/20180406 - F8 on-target.Tif]

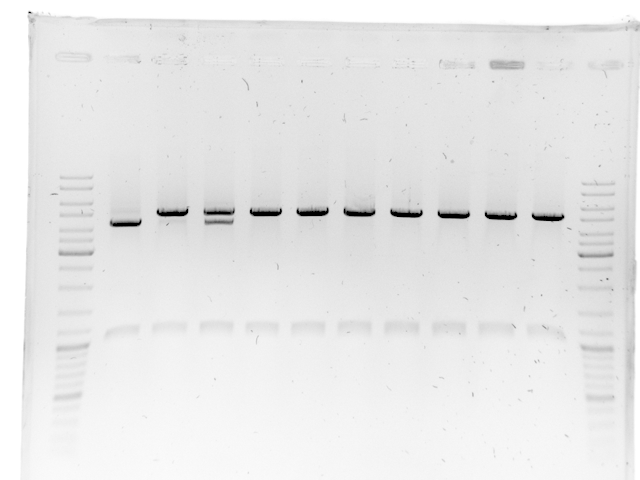

Supplement: Supplementary file 9 — Source data [file 41467_2022_28080_MOESM9_ESM.zip › Source Data/Figure 4a/Figure 4a - D7 asymmetric off-targets.tiff]

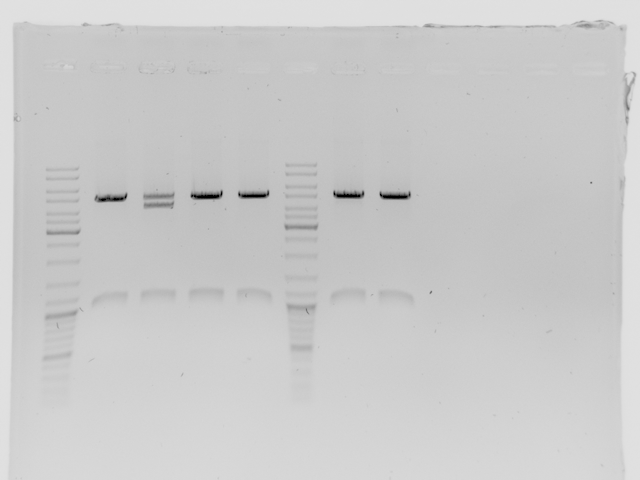

Supplement: Supplementary file 9 — Source data [file 41467_2022_28080_MOESM9_ESM.zip › Source Data/Figure 4a/Figure 4a - D7 symmetric off-targets.tiff]

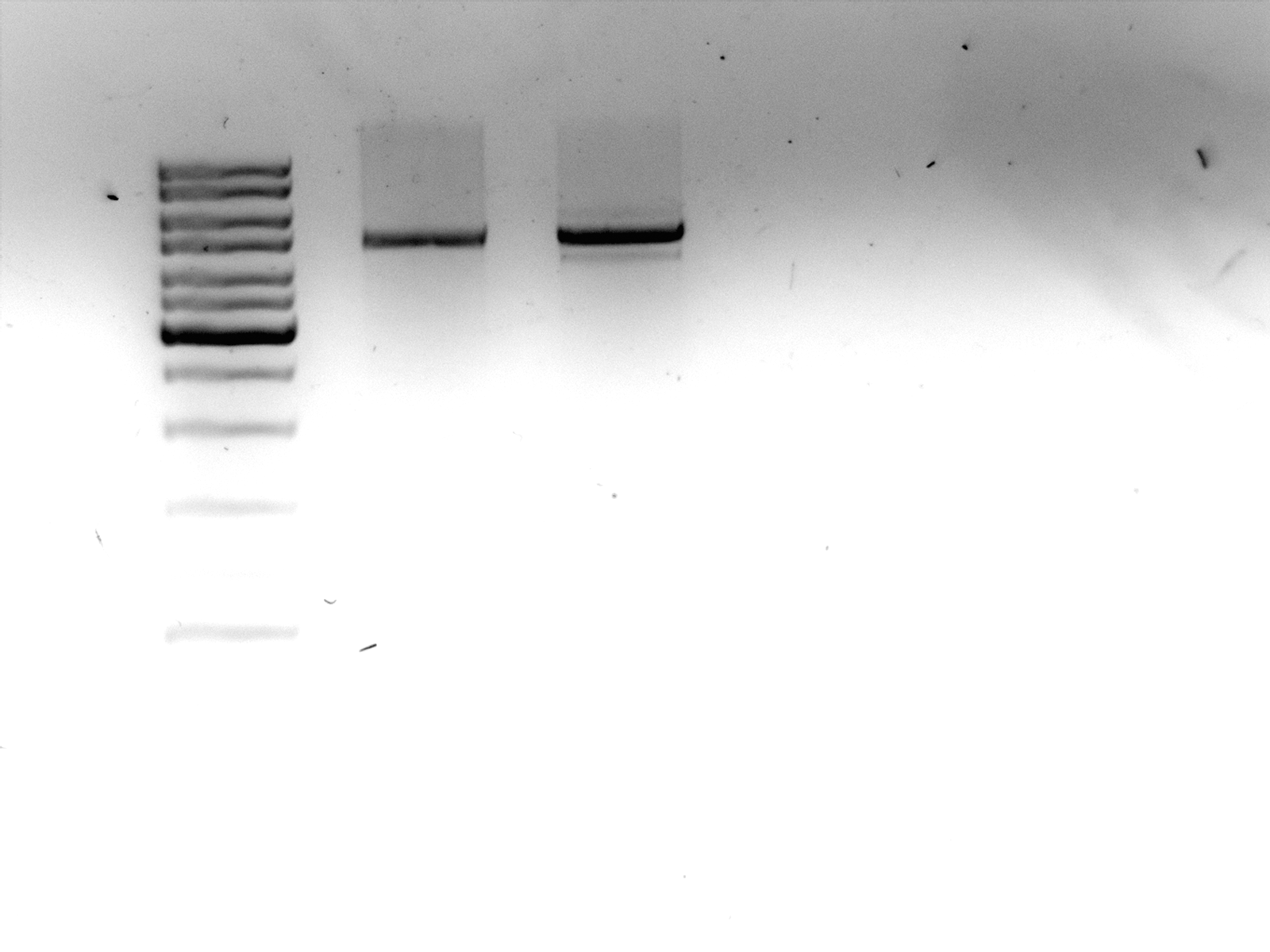

Supplement: Supplementary file 9 — Source data [file 41467_2022_28080_MOESM9_ESM.zip › Source Data/Supp. Figure 2d/Supp. Figure 2d - F8 lib on loxF8.tif]

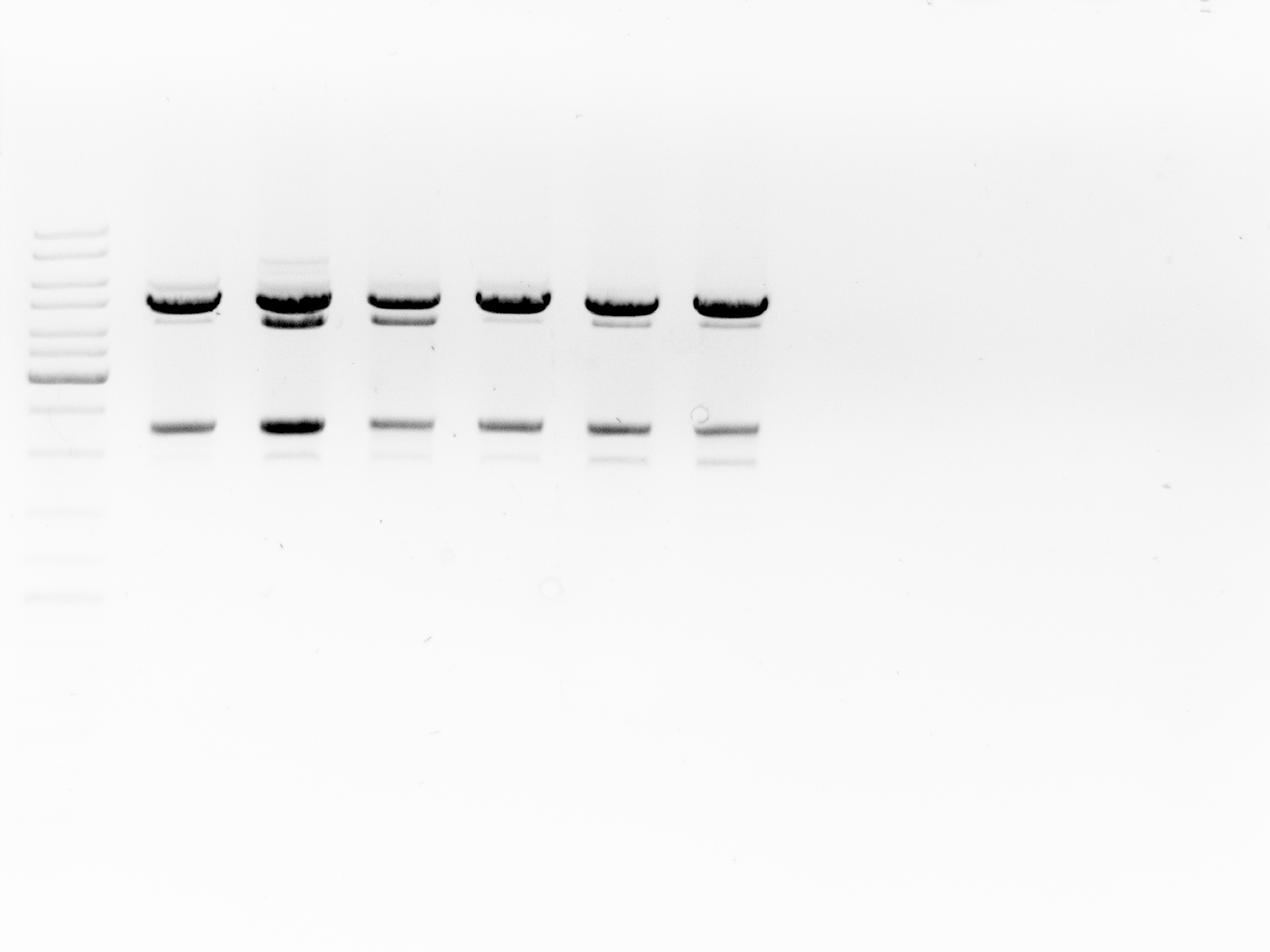

Supplement: Supplementary file 9 — Source data [file 41467_2022_28080_MOESM9_ESM.zip › Source Data/Figure 1d/Figure 1d F8 lib activity.Tif]

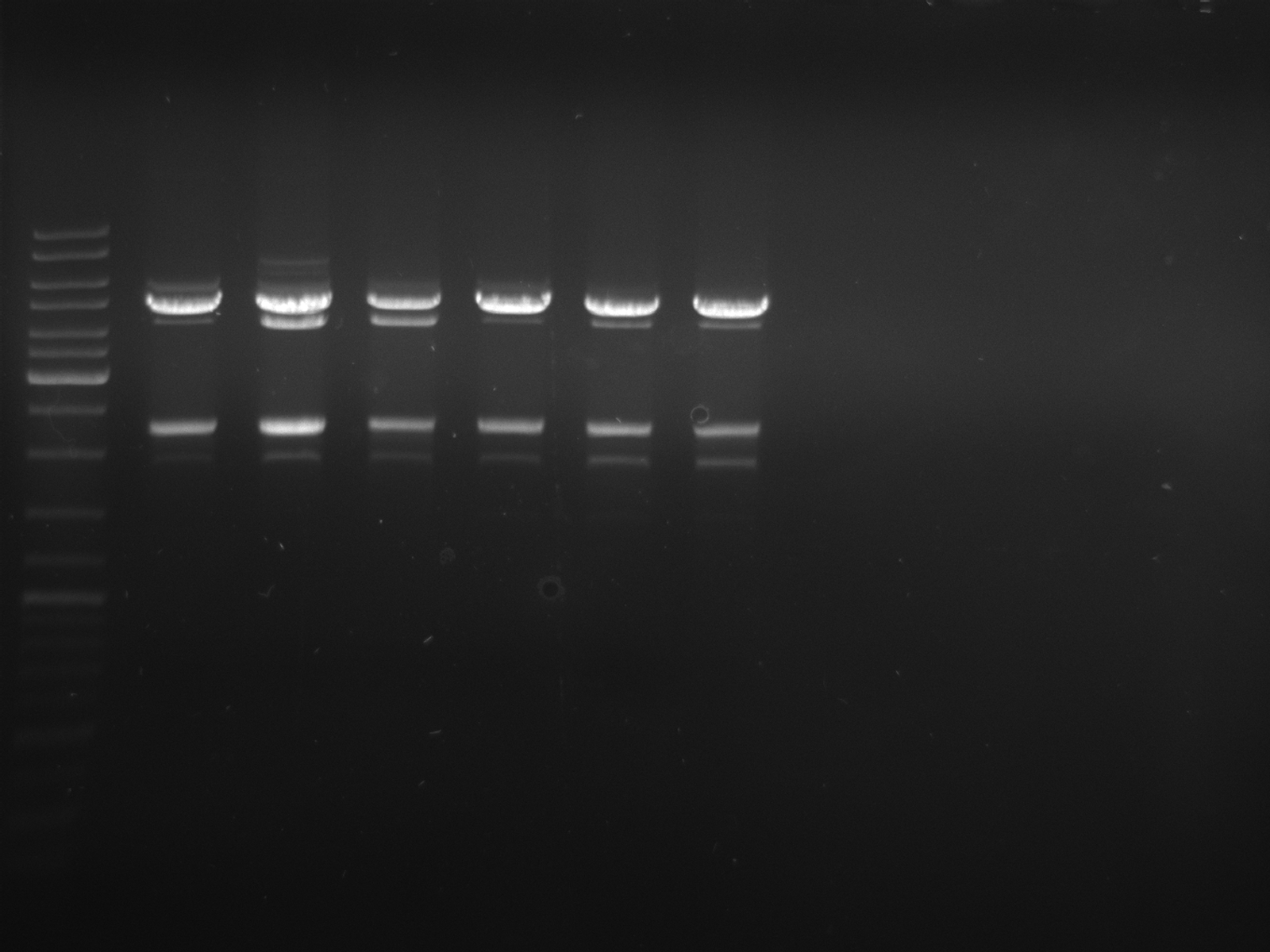

Supplement: Supplementary file 9 — Source data [file 41467_2022_28080_MOESM9_ESM.zip › Source Data/Figure 1d/Figure 1d F8 lib activity - black.Tif]

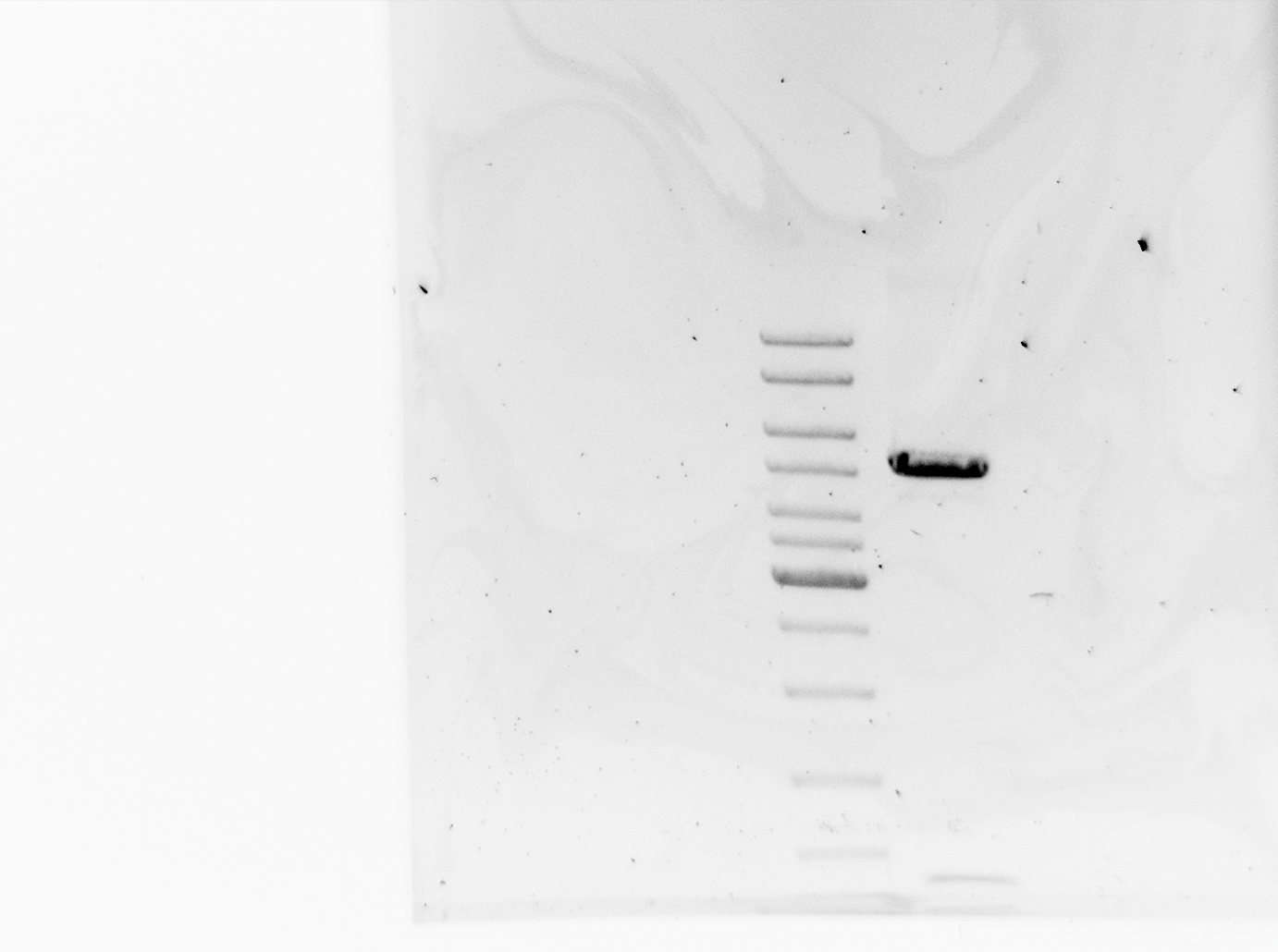

Supplement: Supplementary file 9 — Source data [file 41467_2022_28080_MOESM9_ESM.zip › Source Data/Supp. Figure 2c/cycle53.png]

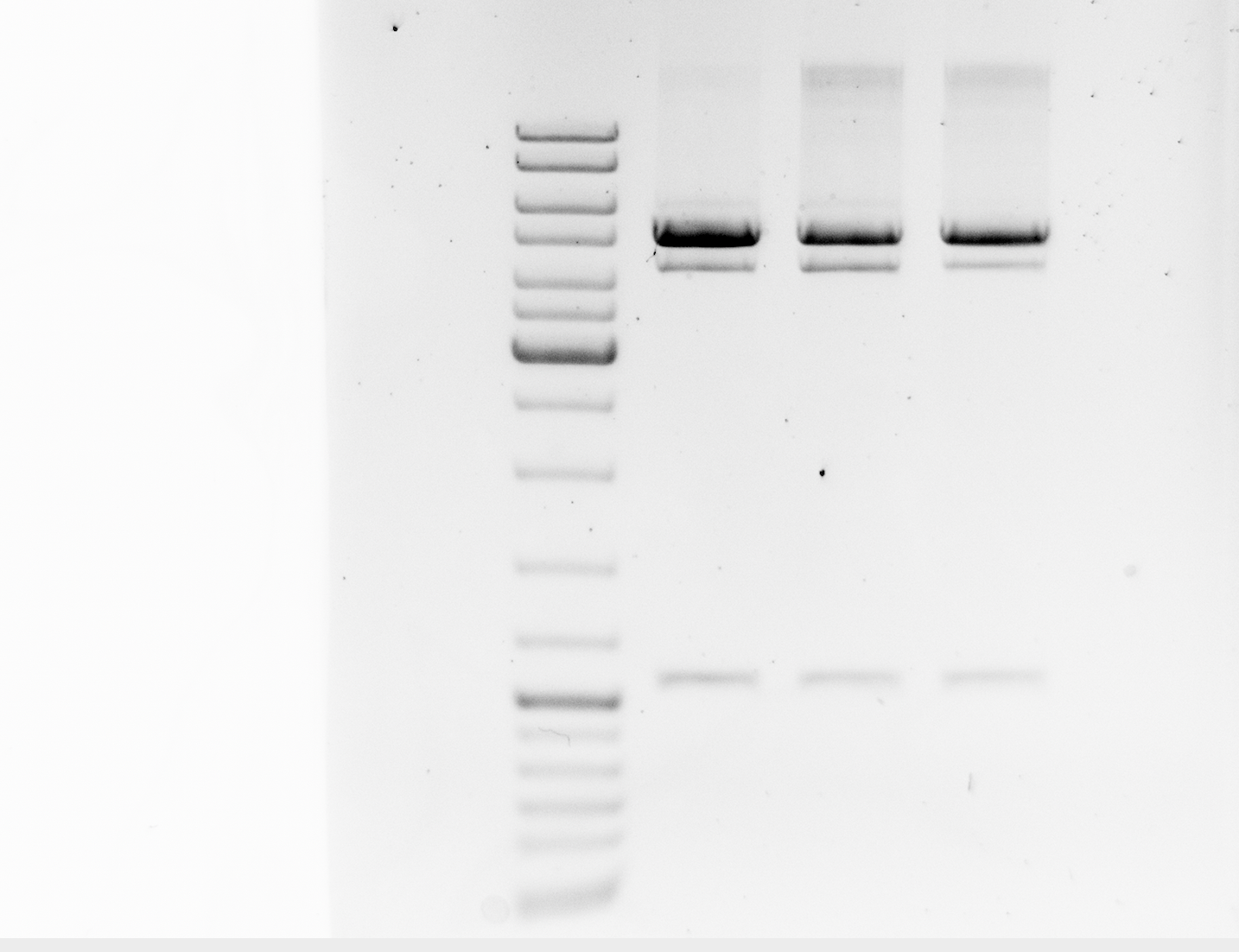

Supplement: Supplementary file 9 — Source data [file 41467_2022_28080_MOESM9_ESM.zip › Source Data/Supp. Figure 2c/cycle39.png]

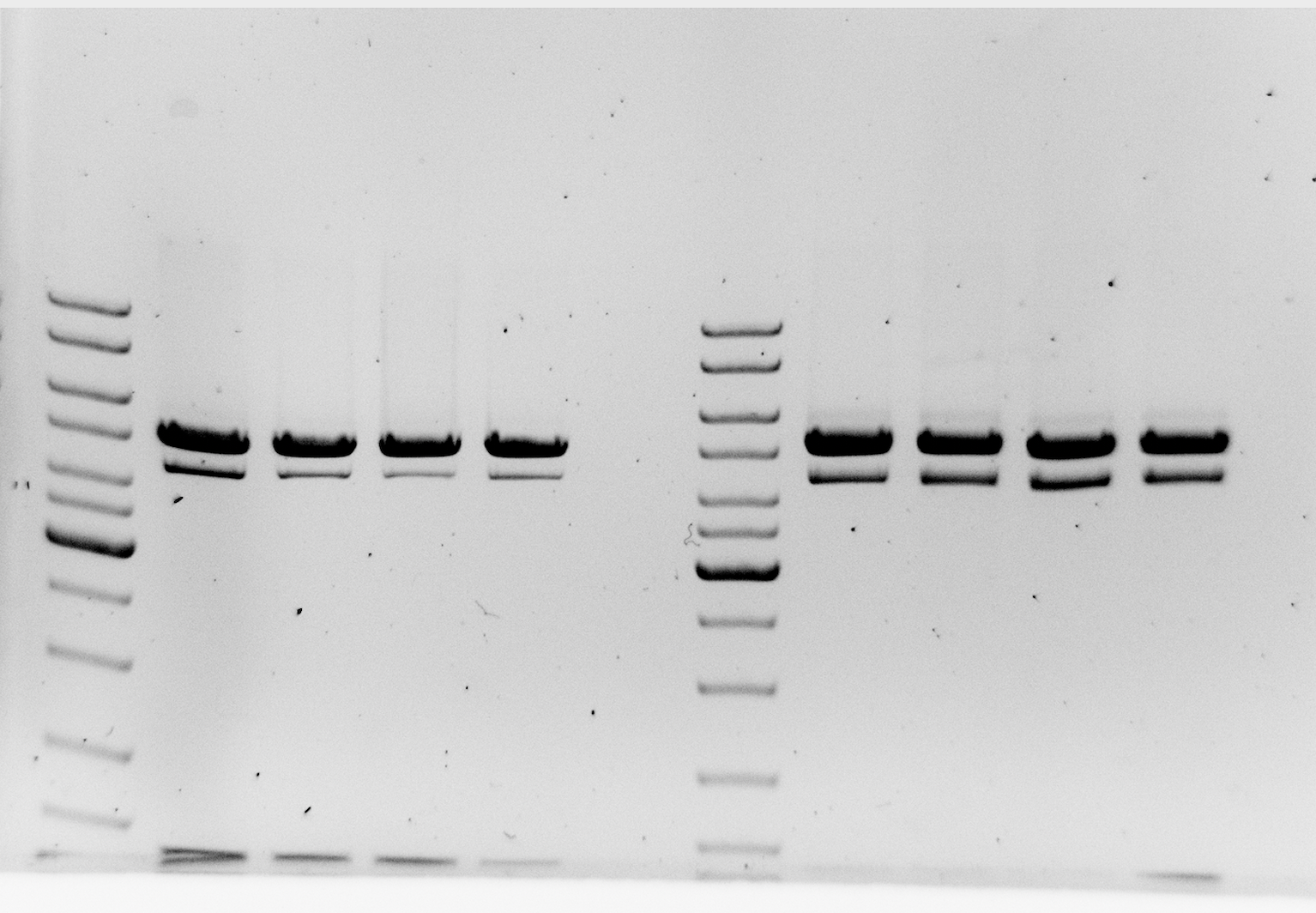

Supplement: Supplementary file 9 — Source data [file 41467_2022_28080_MOESM9_ESM.zip › Source Data/Supp. Figure 2c/cycle12.png]

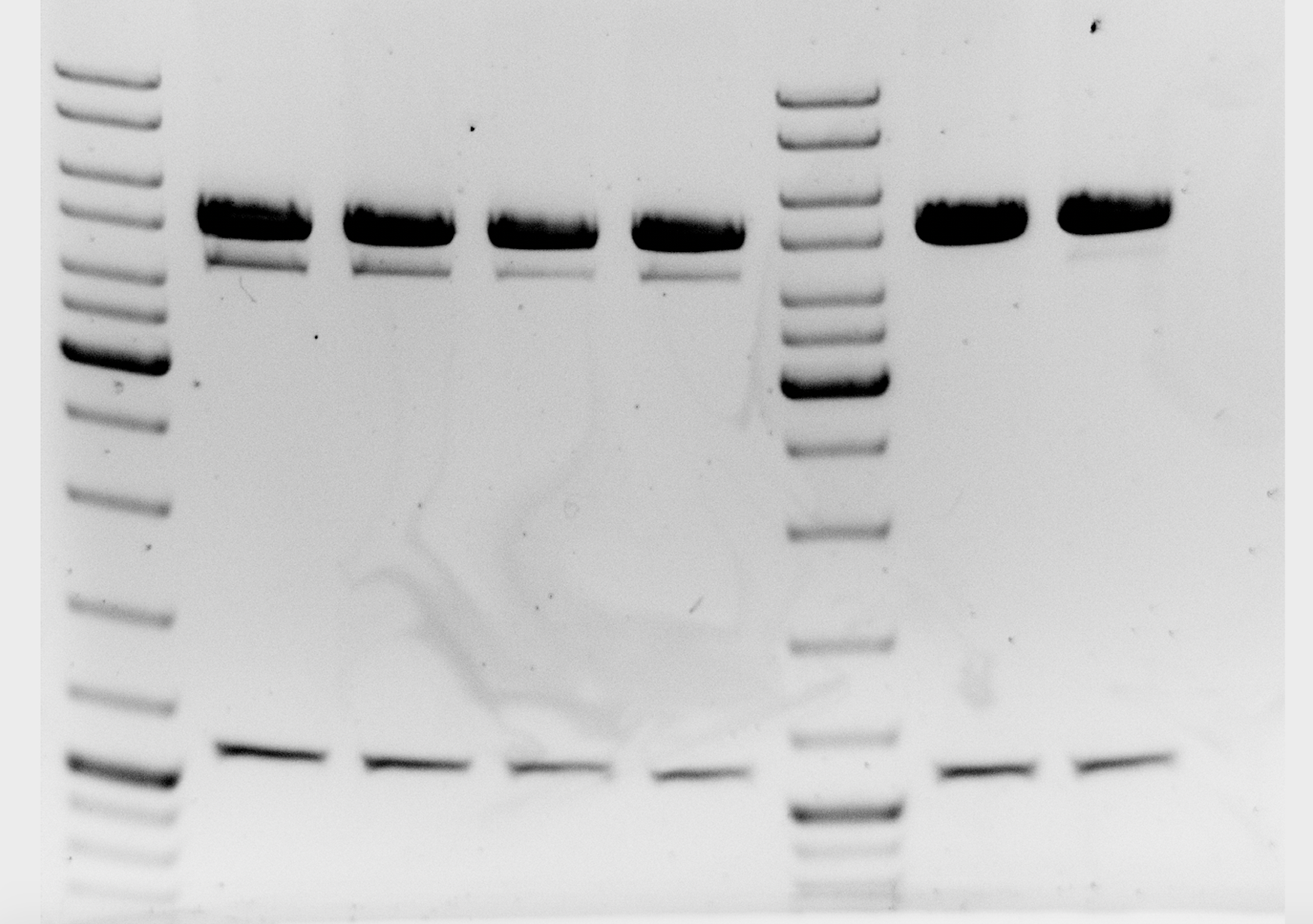

Supplement: Supplementary file 9 — Source data [file 41467_2022_28080_MOESM9_ESM.zip › Source Data/Supp. Figure 2c/cycle13.png]

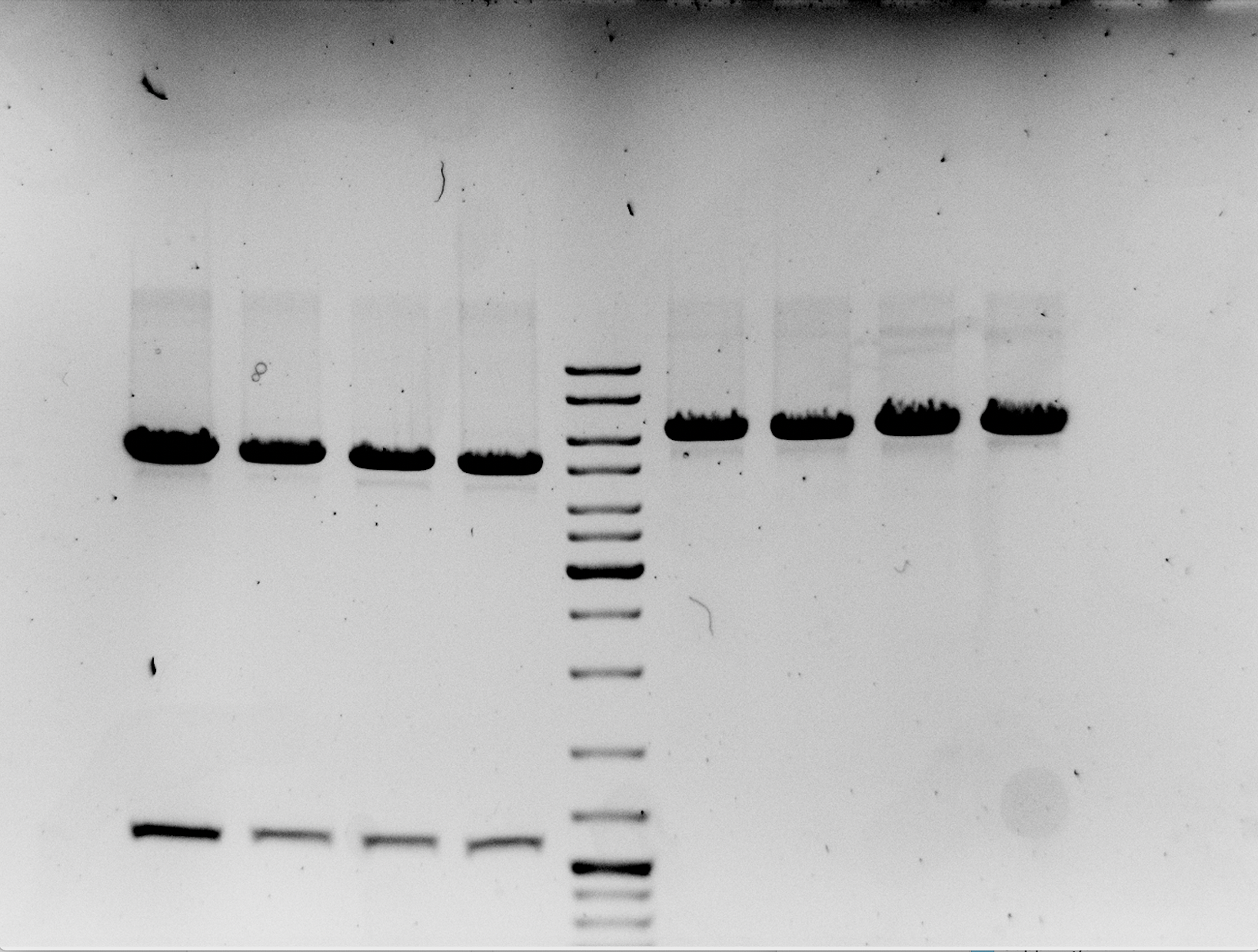

Supplement: Supplementary file 9 — Source data [file 41467_2022_28080_MOESM9_ESM.zip › Source Data/Supp. Figure 2c/cycle1 .png]

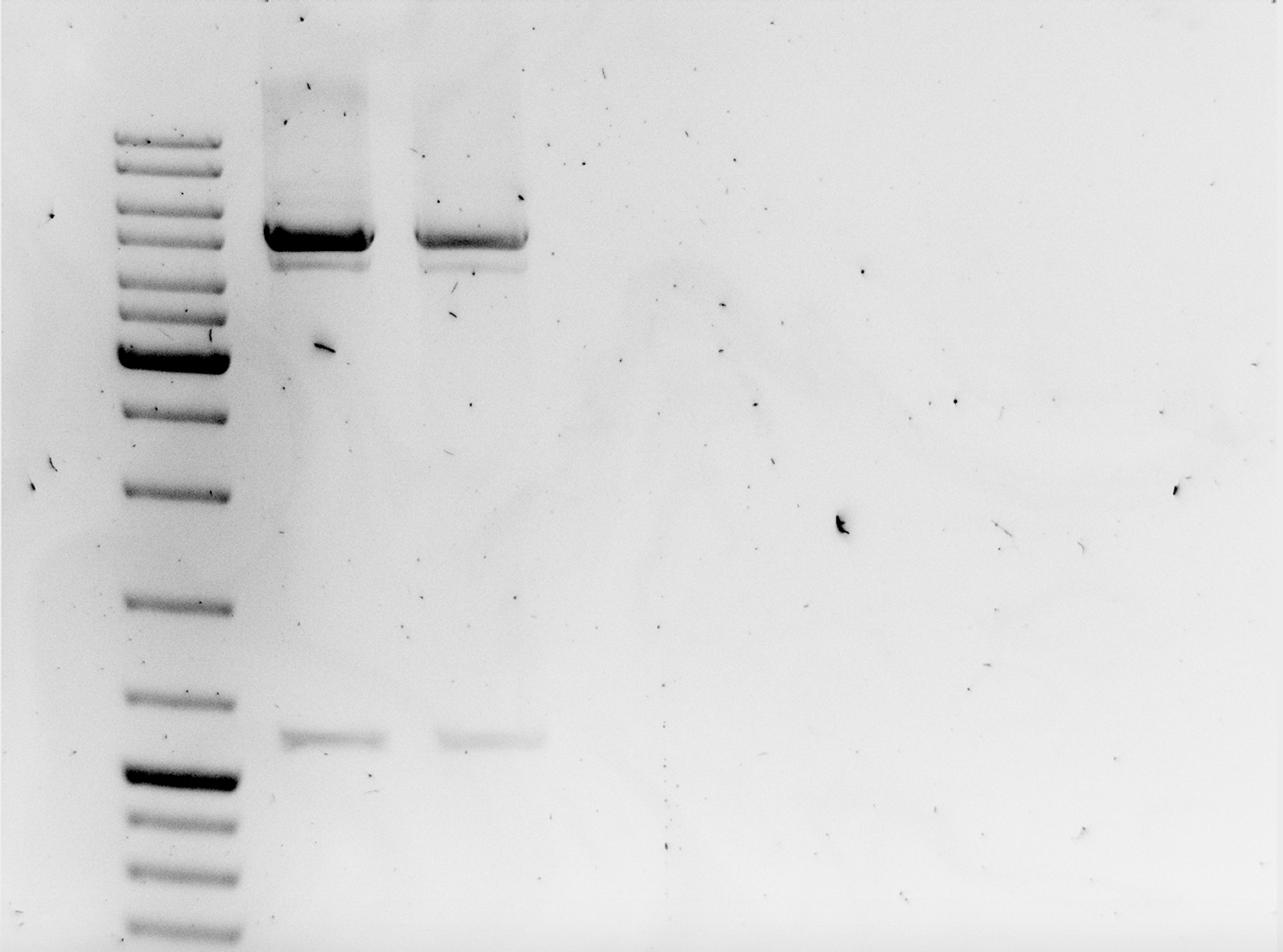

Supplement: Supplementary file 9 — Source data [file 41467_2022_28080_MOESM9_ESM.zip › Source Data/Supp. Figure 2c/cycle65.png]

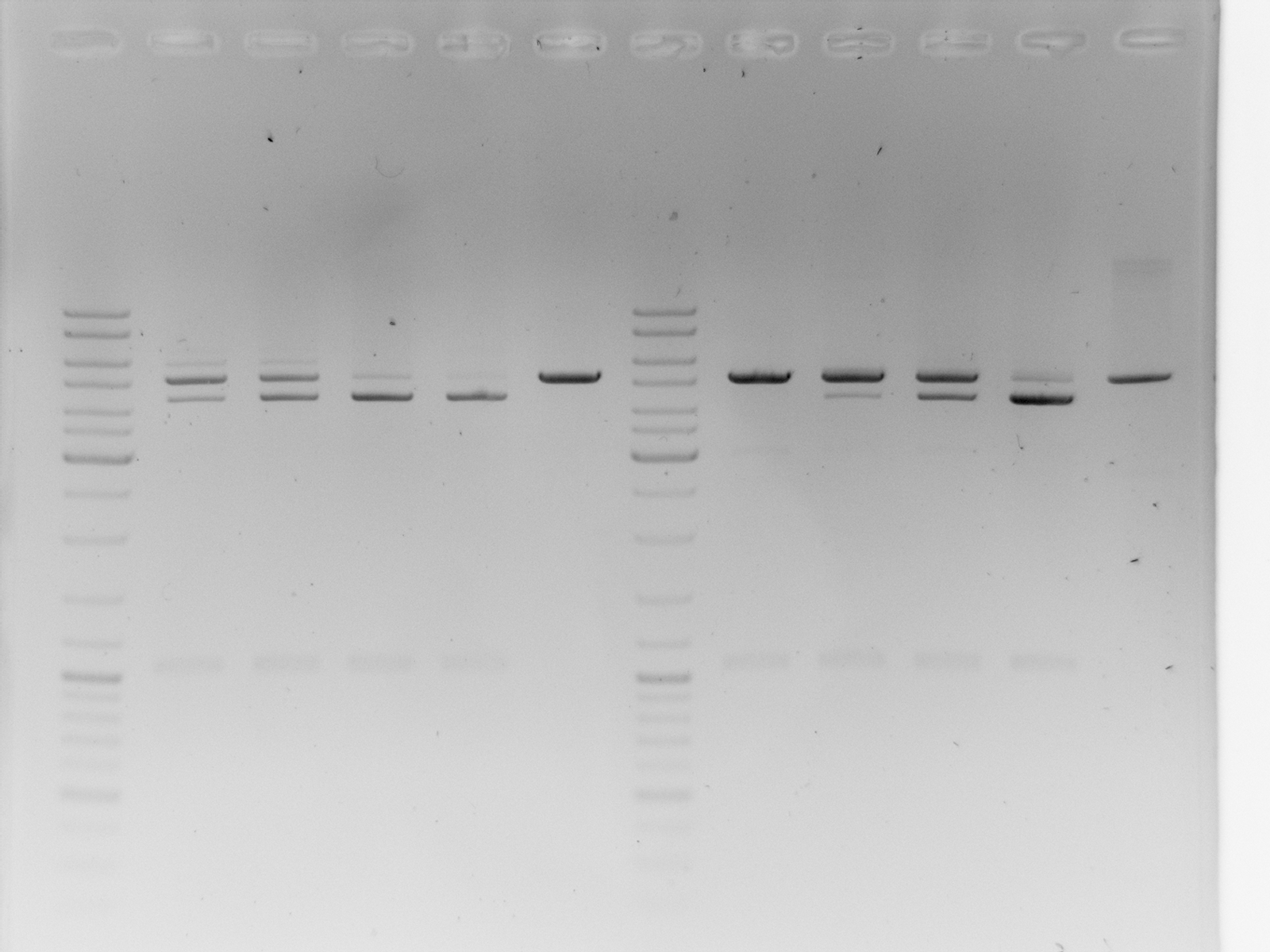

Supplement: Supplementary file 9 — Source data [file 41467_2022_28080_MOESM9_ESM.zip › Source Data/Figure 2b/Figure 2b - Cre on loxP and Brec1 on loxBTR.Tif]

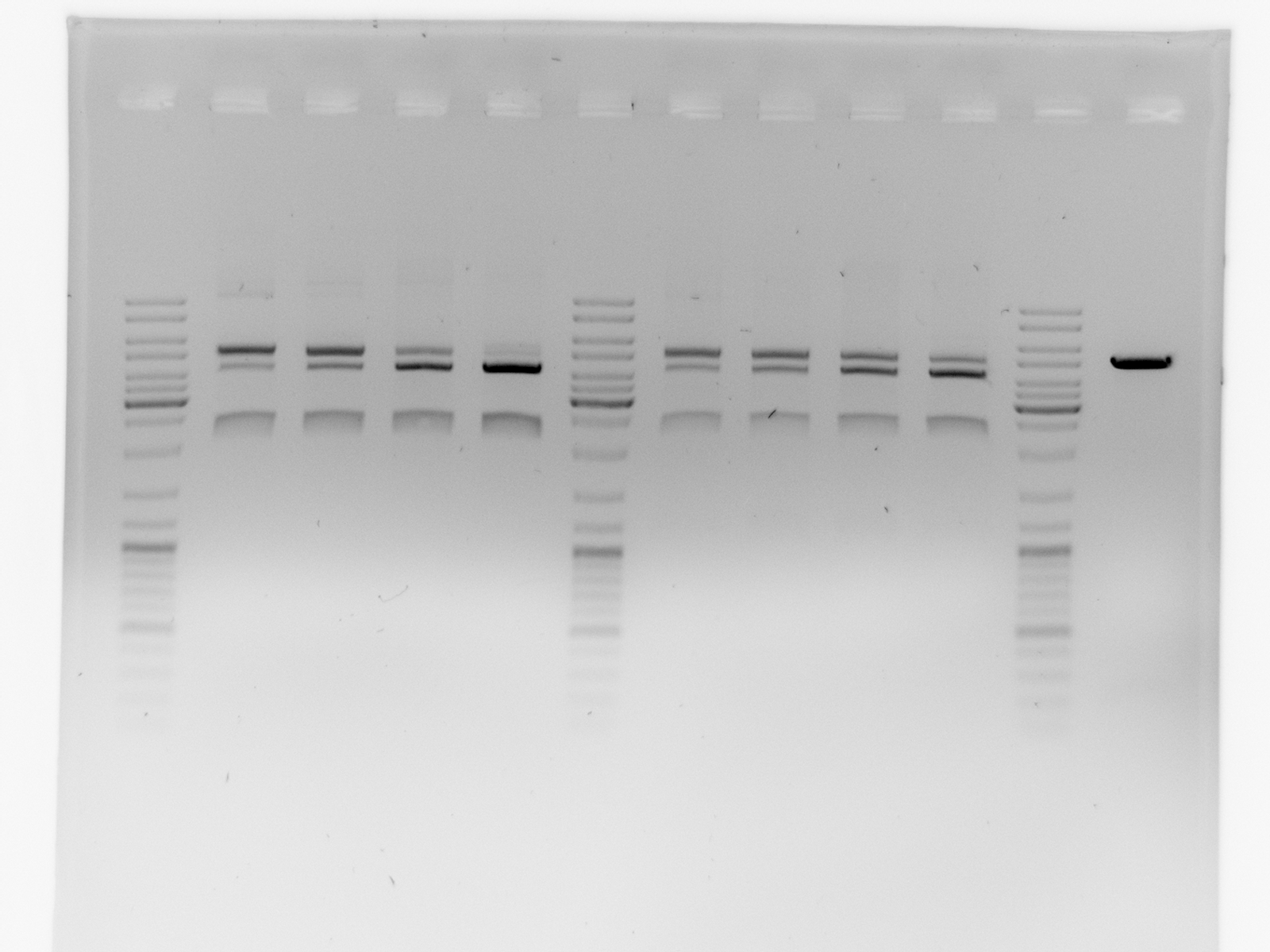

Supplement: Supplementary file 9 — Source data [file 41467_2022_28080_MOESM9_ESM.zip › Source Data/Figure 2b/Figure 2b - D7 on loxF8.Tif]

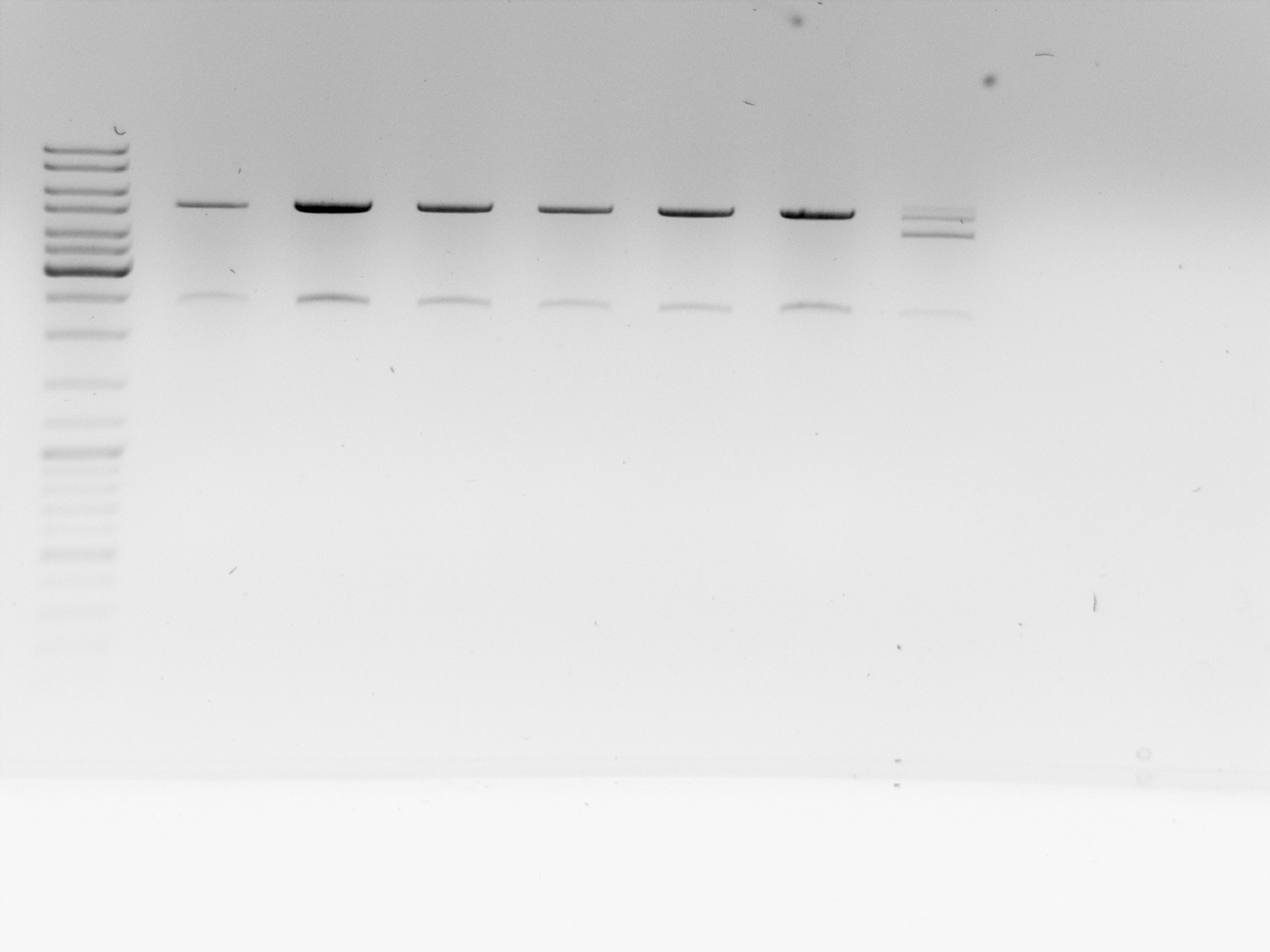

Supplement: Supplementary file 9 — Source data [file 41467_2022_28080_MOESM9_ESM.zip › Source Data/Figure 4g/Figure 4g - peak 7-12 and loxF8.Tif]

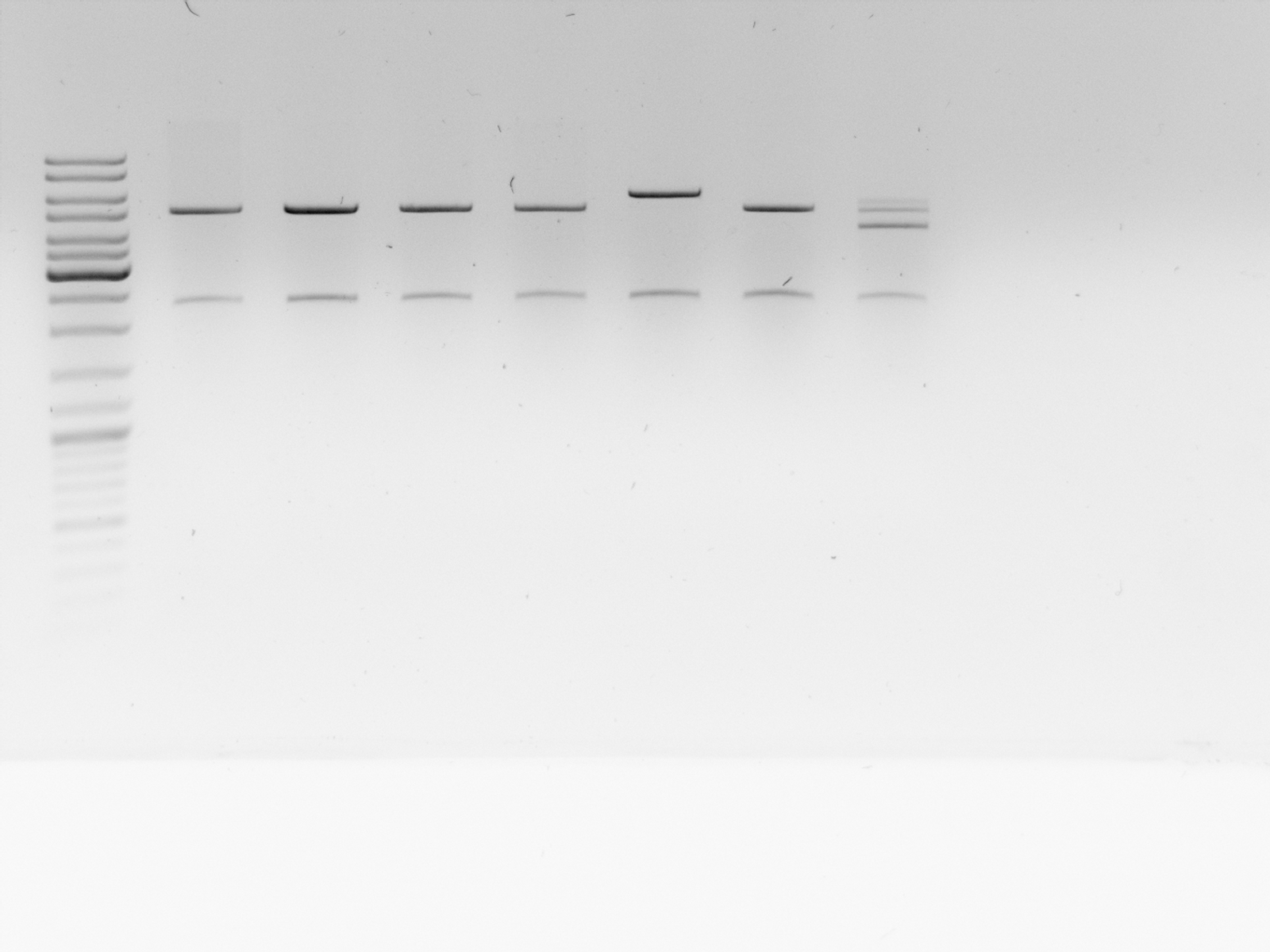

Supplement: Supplementary file 9 — Source data [file 41467_2022_28080_MOESM9_ESM.zip › Source Data/Figure 4g/Figure 4g - Peak 1-6 and loxF8.Tif]

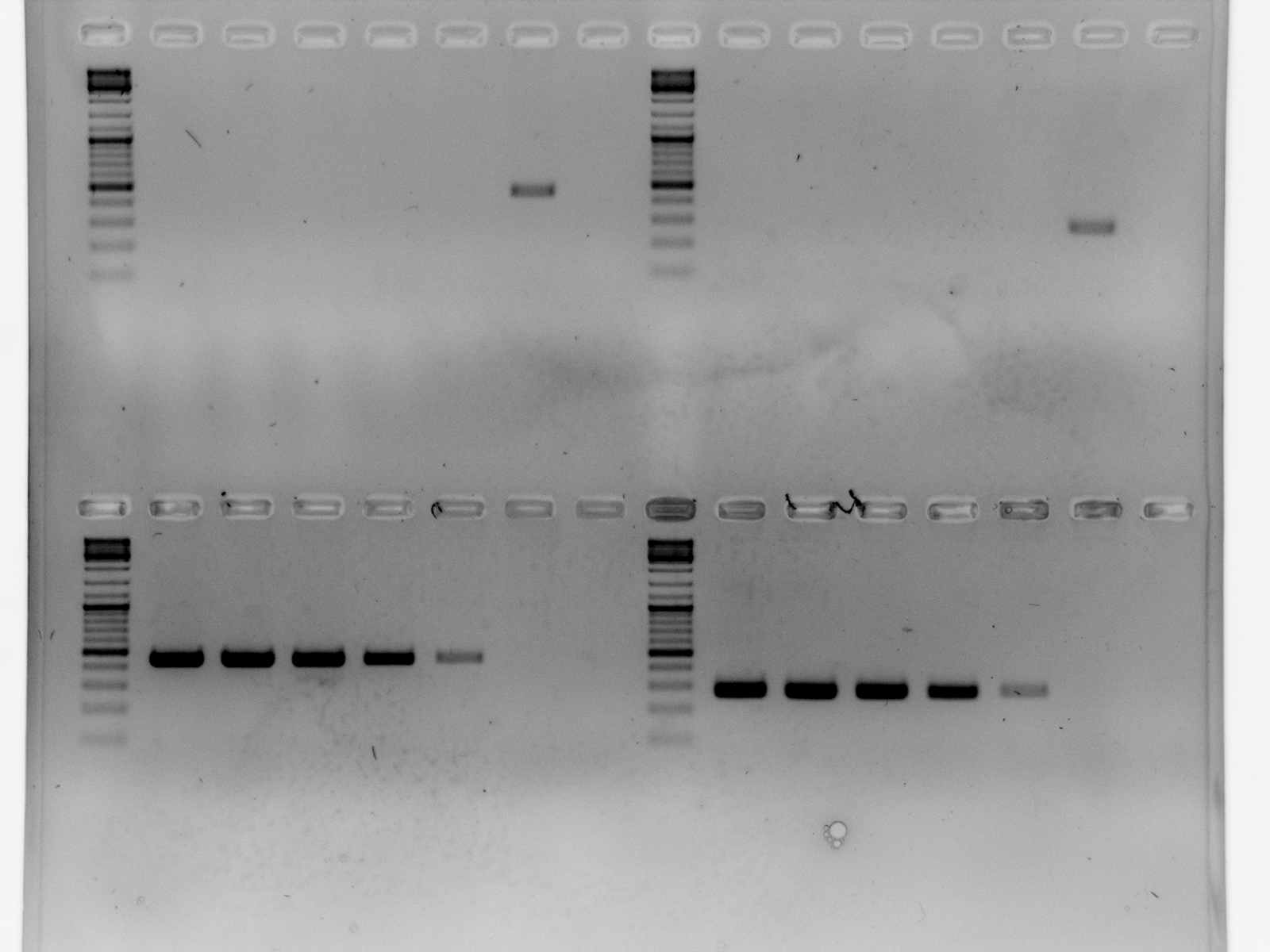

Supplement: Supplementary file 9 — Source data [file 41467_2022_28080_MOESM9_ESM.zip › Source Data/Supp. Figure 16d/Supp. Figure 16d - HG2off-target testing upper part of the gel.tif]

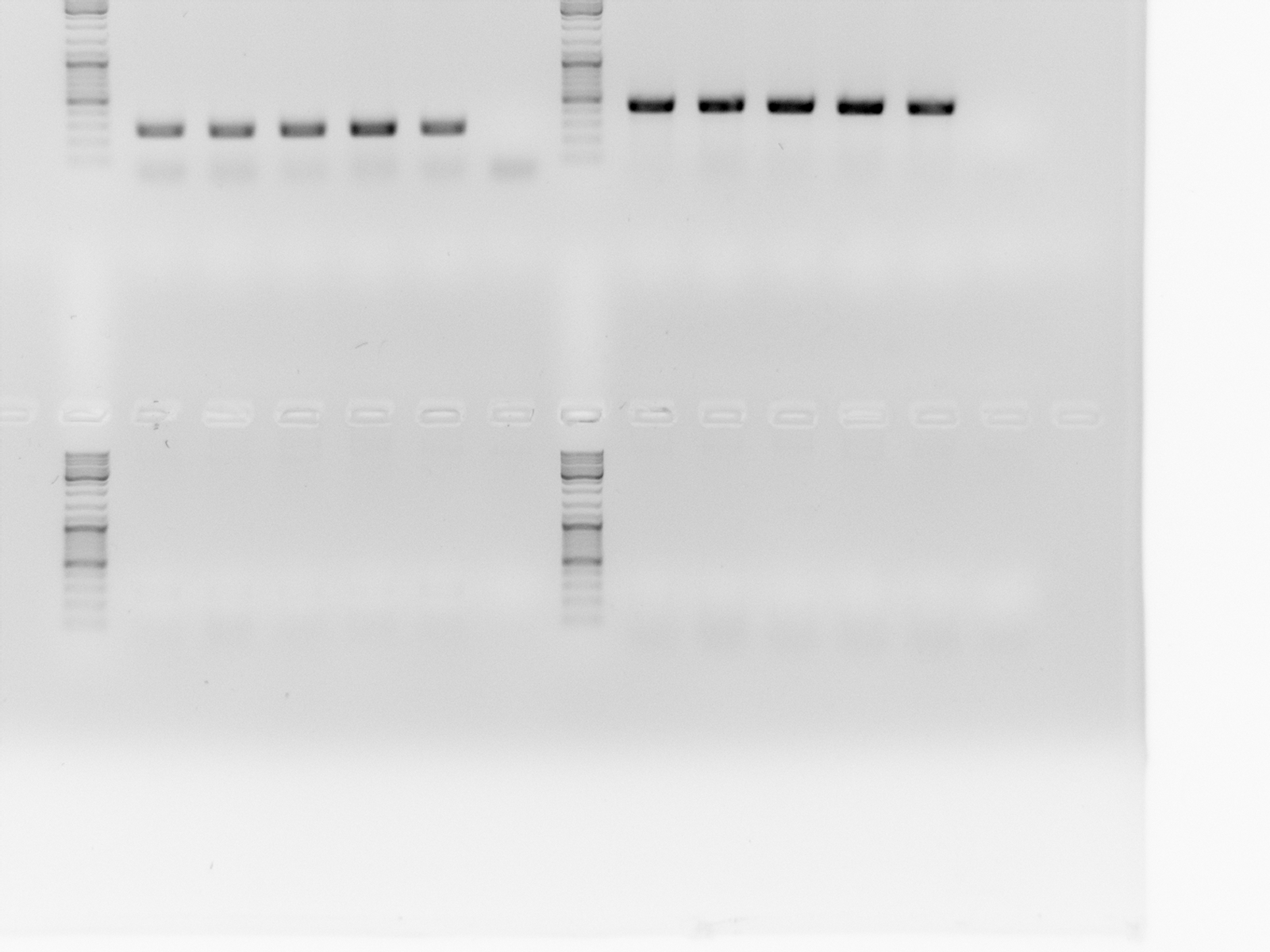

Supplement: Supplementary file 9 — Source data [file 41467_2022_28080_MOESM9_ESM.zip › Source Data/Supp. Figure 16c/Supp. Figure 16c - HG translocation positive .tif]

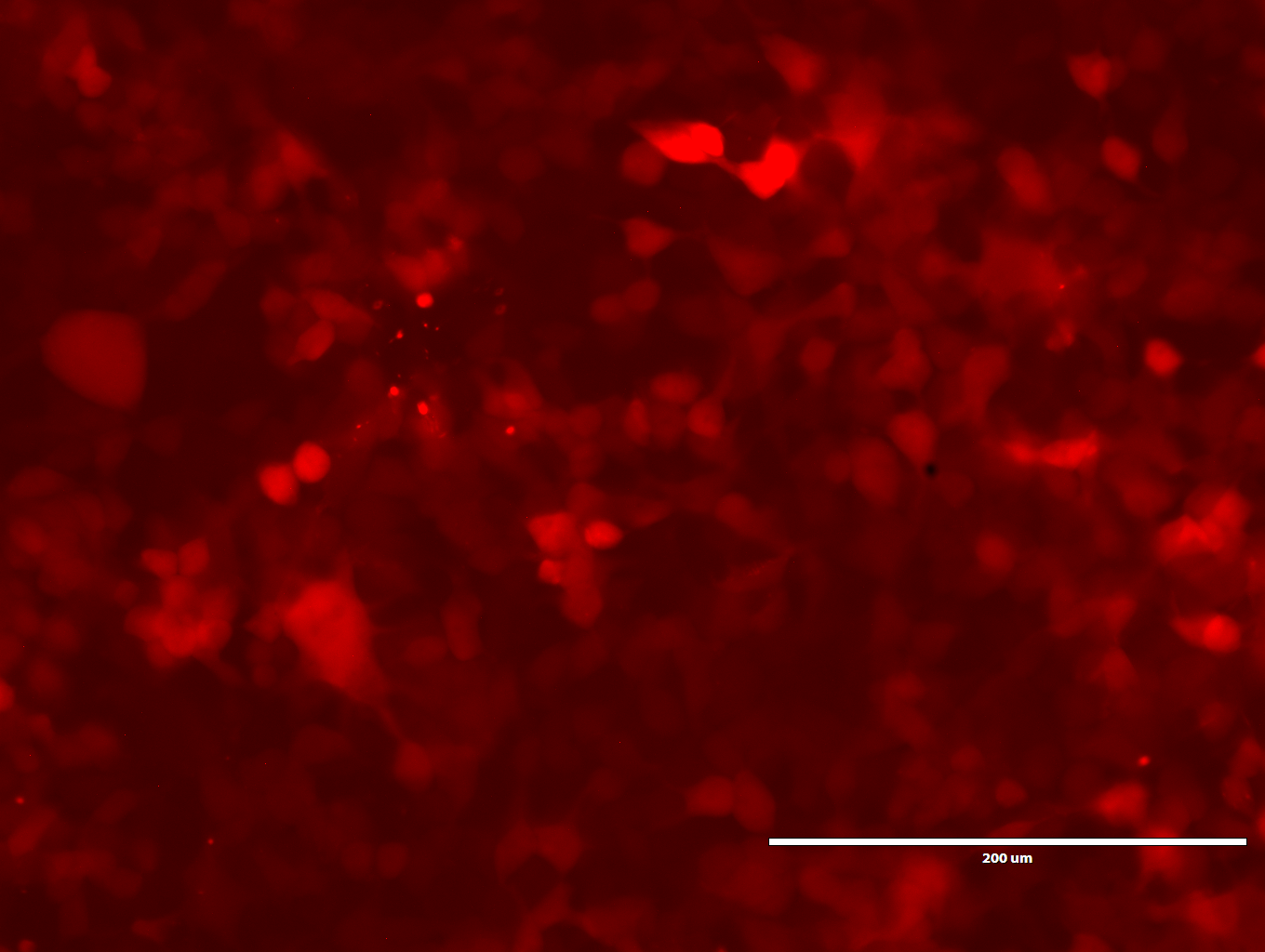

Supplement: Supplementary file 9 — Source data [file 41467_2022_28080_MOESM9_ESM.zip › Source Data/Supp. Figure 10a/Supp. Figure 10a - Cre+ mCherry.tif]

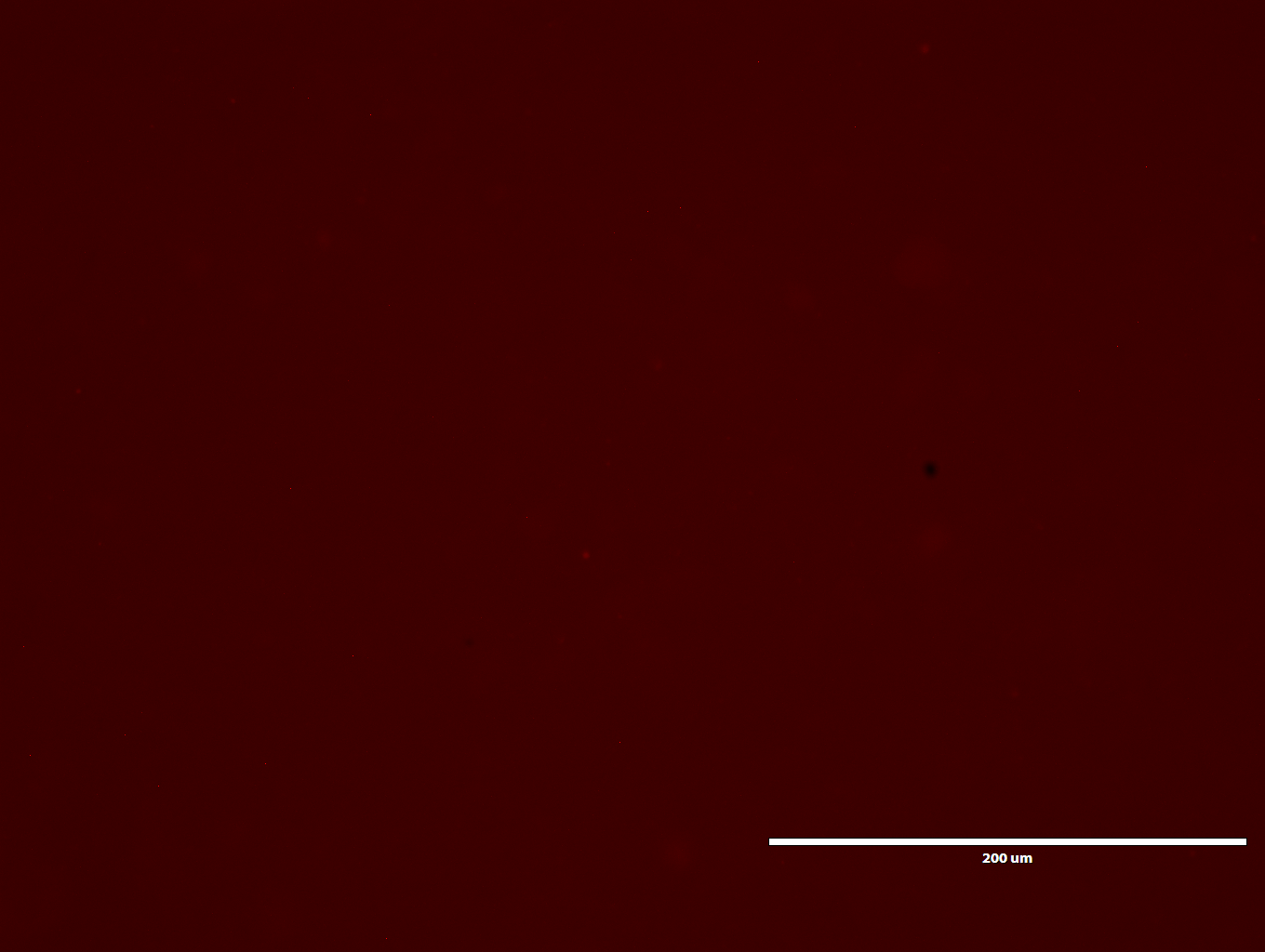

Supplement: Supplementary file 9 — Source data [file 41467_2022_28080_MOESM9_ESM.zip › Source Data/Supp. Figure 10a/Supp. Figure 10a - Cre- mCherry.tif]

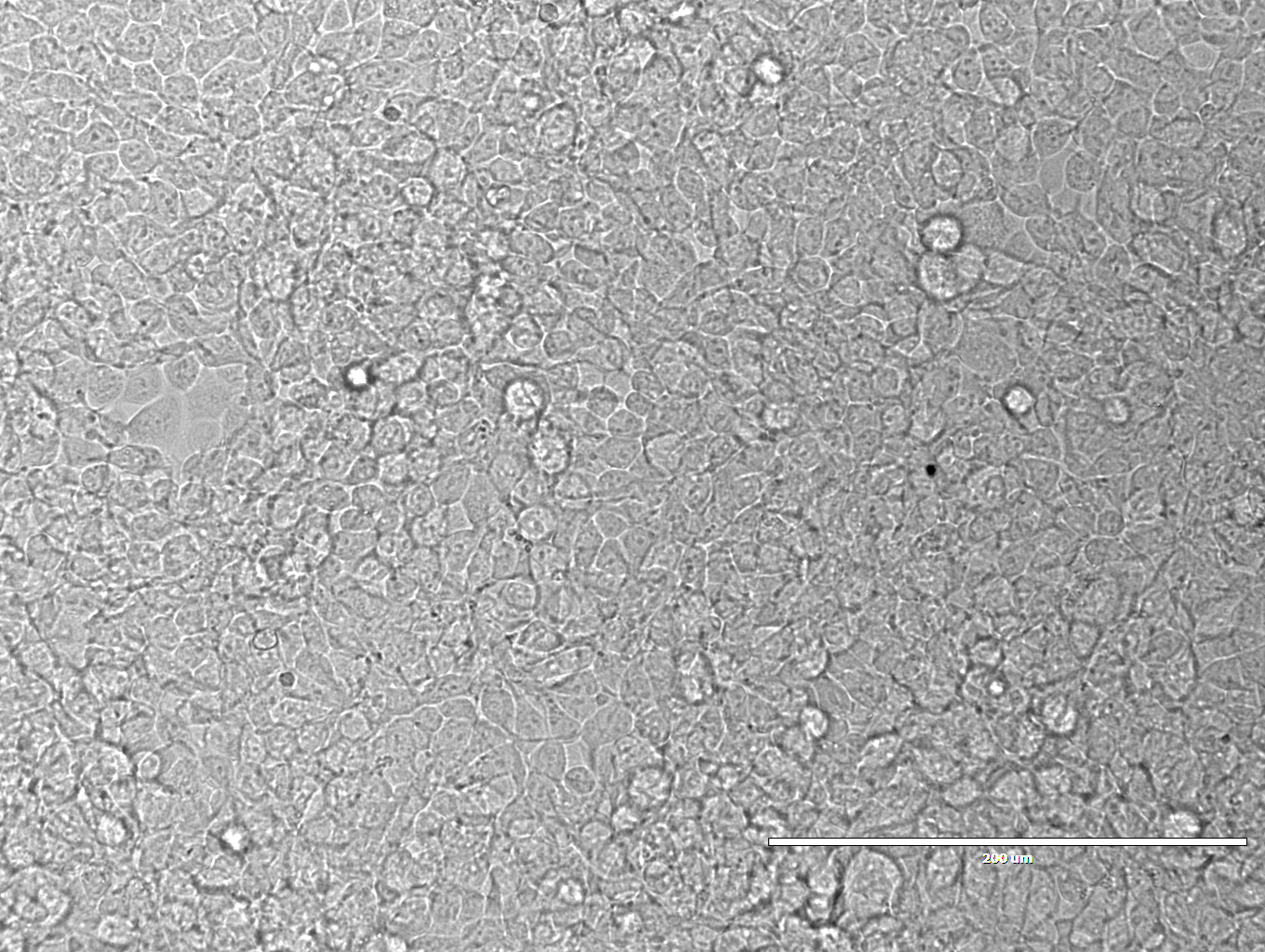

Supplement: Supplementary file 9 — Source data [file 41467_2022_28080_MOESM9_ESM.zip › Source Data/Supp. Figure 10a/Supp. Figure 10a - Cre- BF.tif]

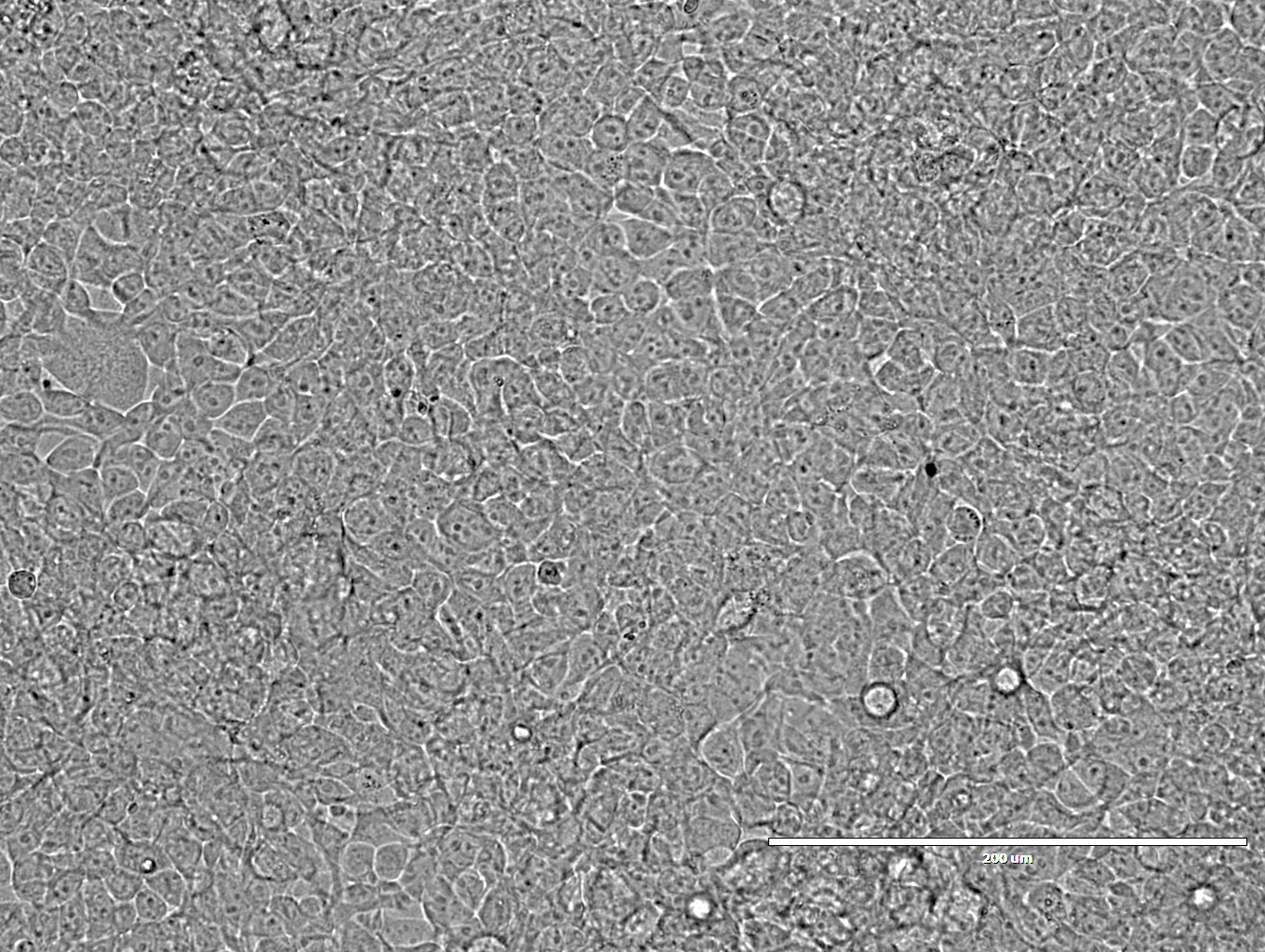

Supplement: Supplementary file 9 — Source data [file 41467_2022_28080_MOESM9_ESM.zip › Source Data/Supp. Figure 10a/Supp. Figure 10a - Cre+ BF.tif]

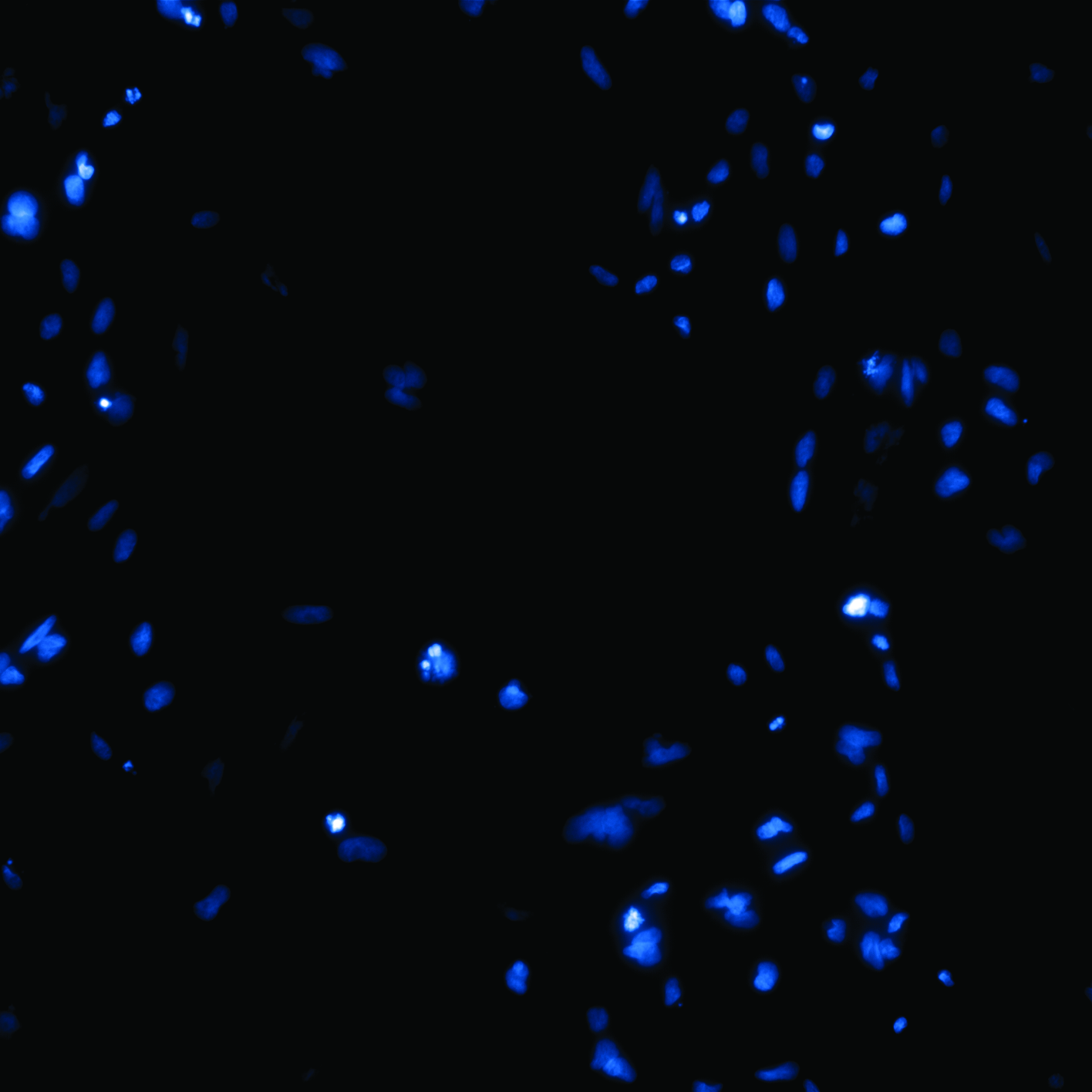

Supplement: Supplementary file 9 — Source data [file 41467_2022_28080_MOESM9_ESM.zip › Source Data/Supp. Figure 19f/Supp. Figure 19f - WT EC 2nd only merge.tif]

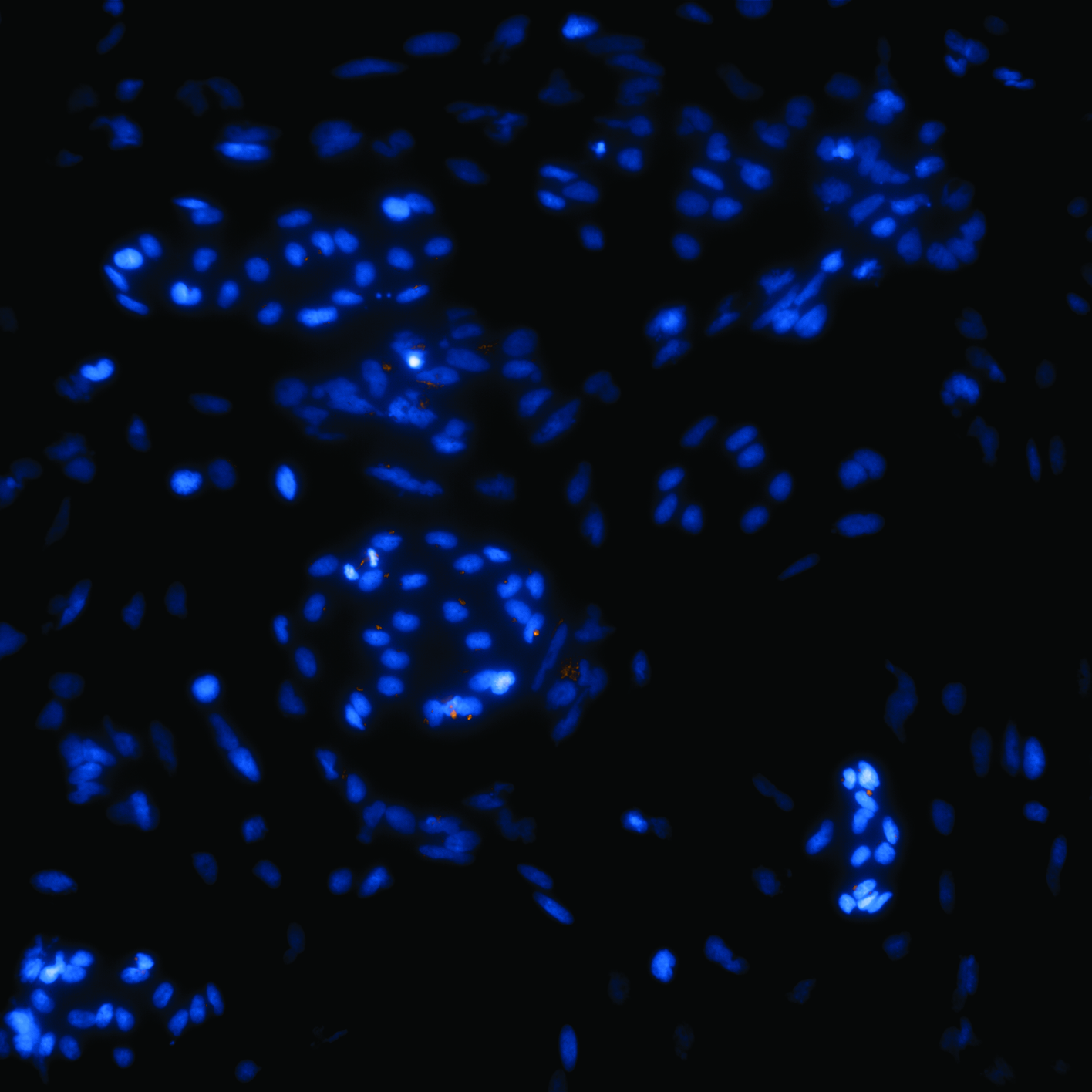

Supplement: Supplementary file 9 — Source data [file 41467_2022_28080_MOESM9_ESM.zip › Source Data/Supp. Figure 19f/Supp. Figure 19f - F8 EC merge.tif]

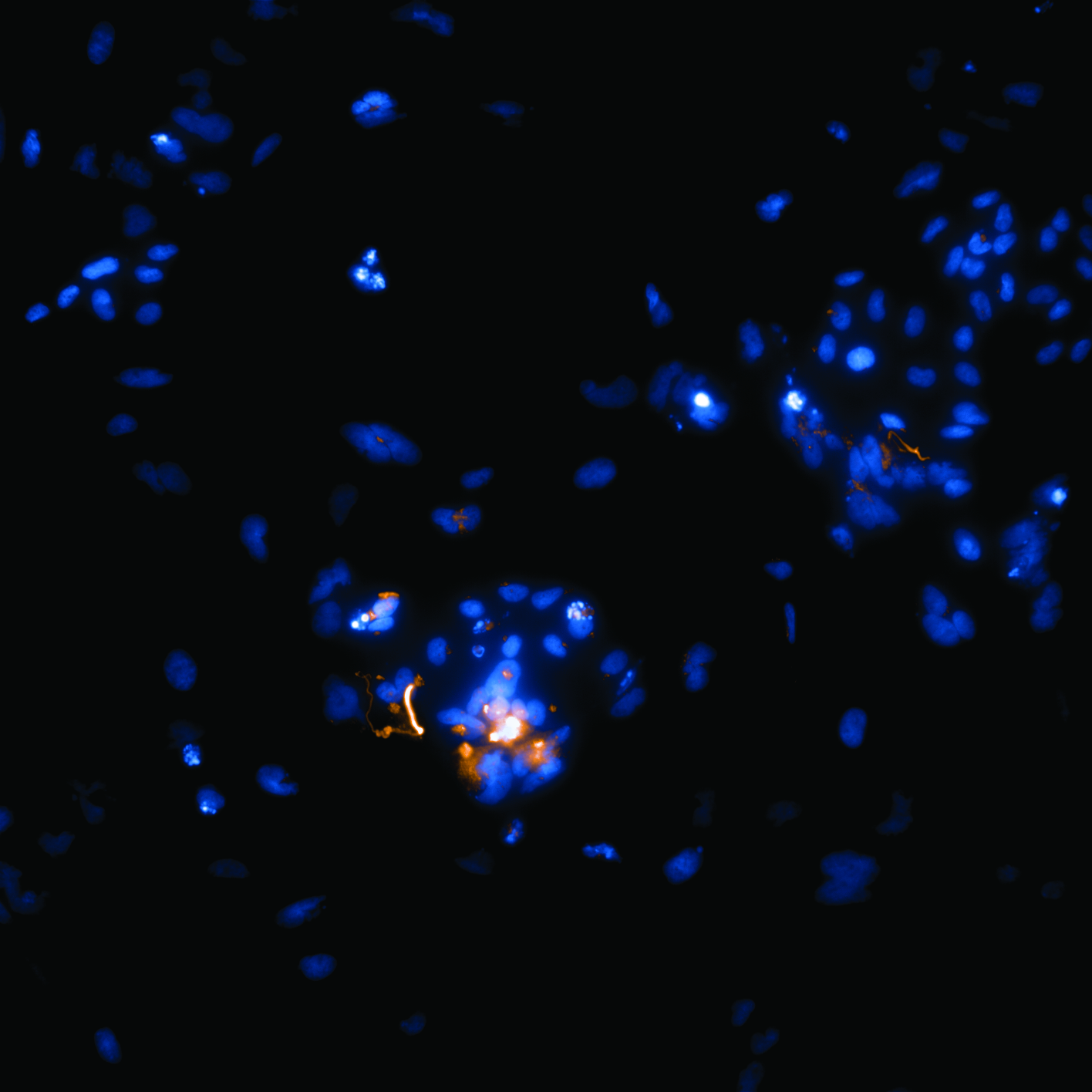

Supplement: Supplementary file 9 — Source data [file 41467_2022_28080_MOESM9_ESM.zip › Source Data/Supp. Figure 19f/Supp. Figure 19f - F8+RecF8 EC merge.tif]

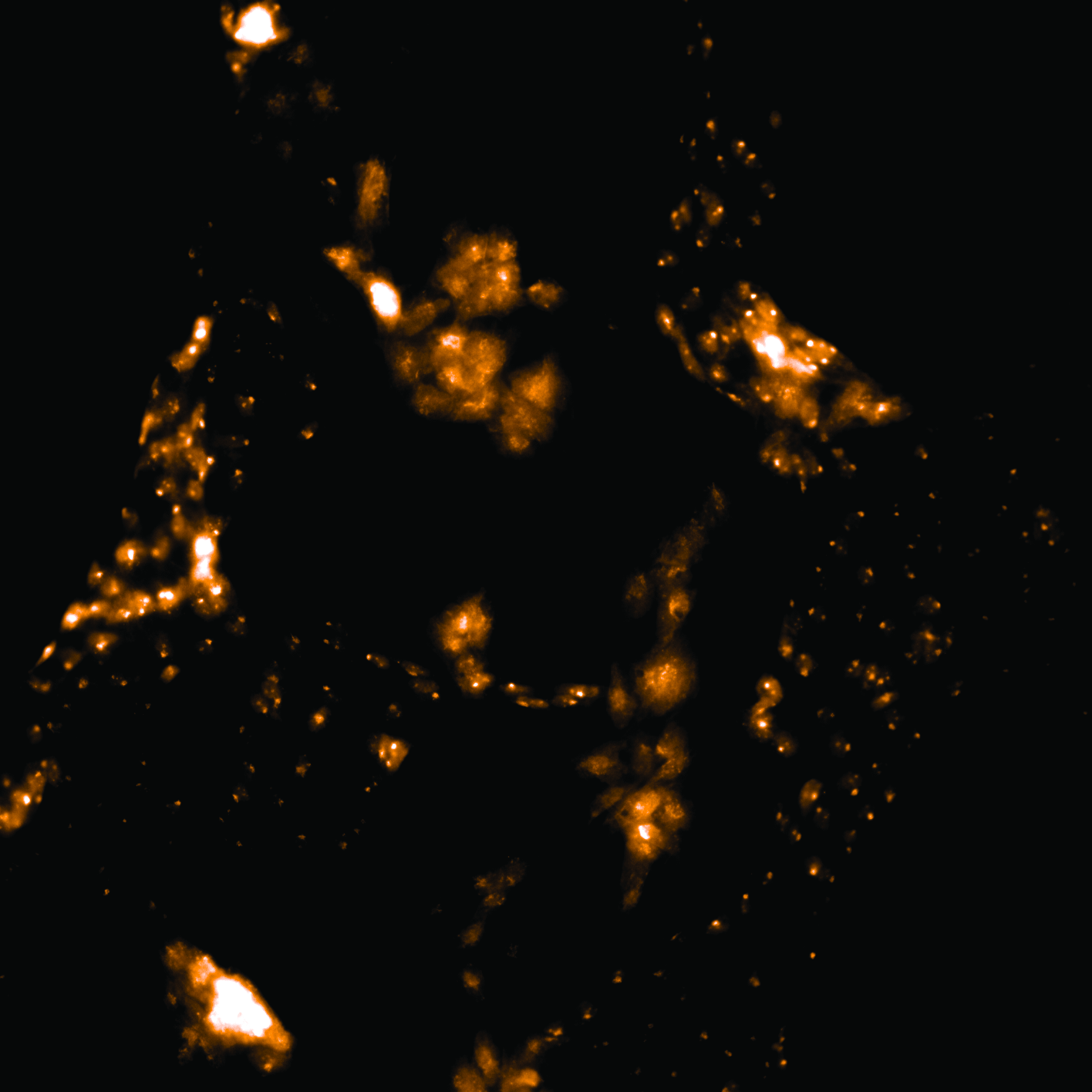

Supplement: Supplementary file 9 — Source data [file 41467_2022_28080_MOESM9_ESM.zip › Source Data/Supp. Figure 19f/Supp. Figure 19f - WT EC F8.tif]

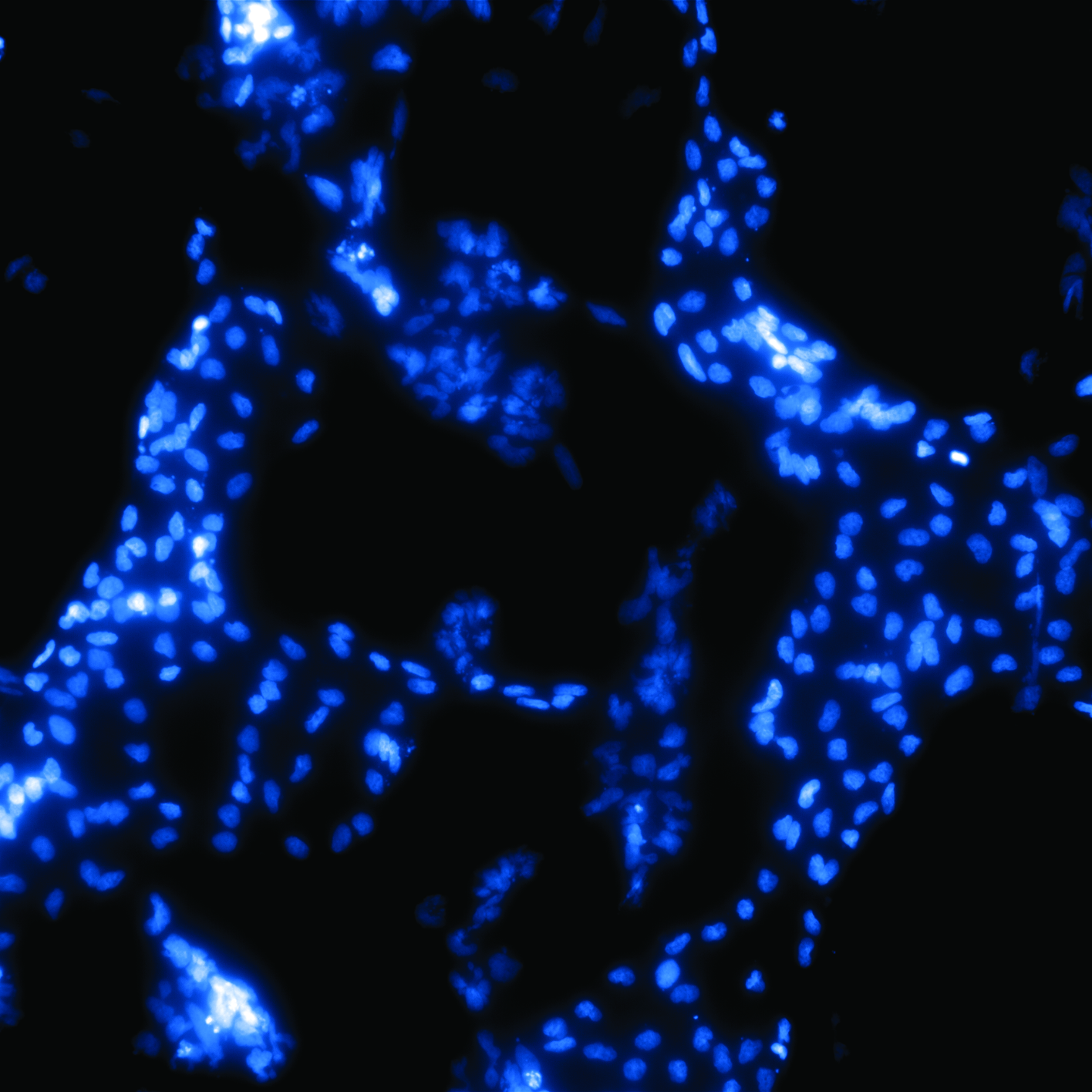

Supplement: Supplementary file 9 — Source data [file 41467_2022_28080_MOESM9_ESM.zip › Source Data/Supp. Figure 19f/Supp. Figure 19f - WT EC DAPI.tif]

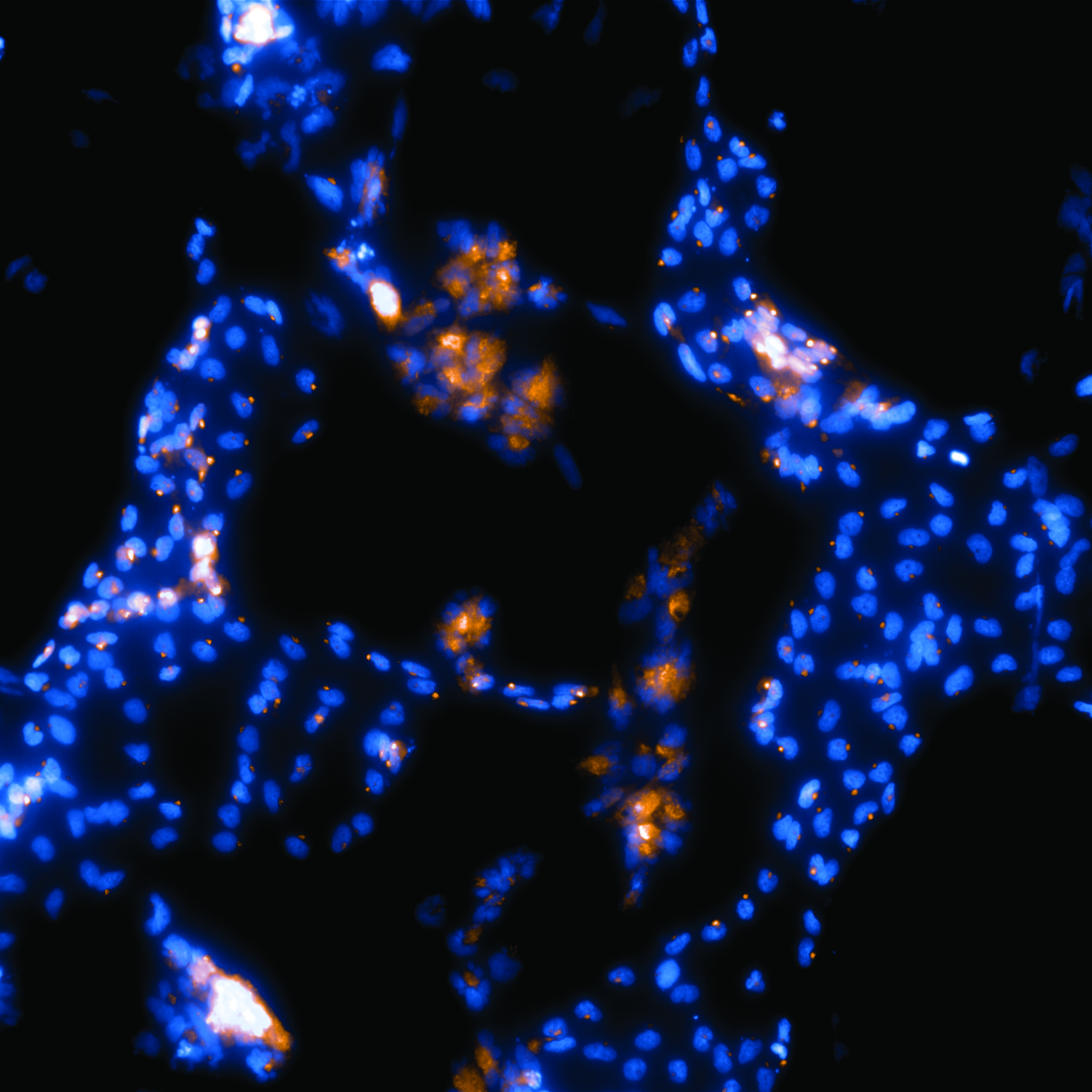

Supplement: Supplementary file 9 — Source data [file 41467_2022_28080_MOESM9_ESM.zip › Source Data/Supp. Figure 19f/Supp. Figure 19f - WT EC merge.tif]

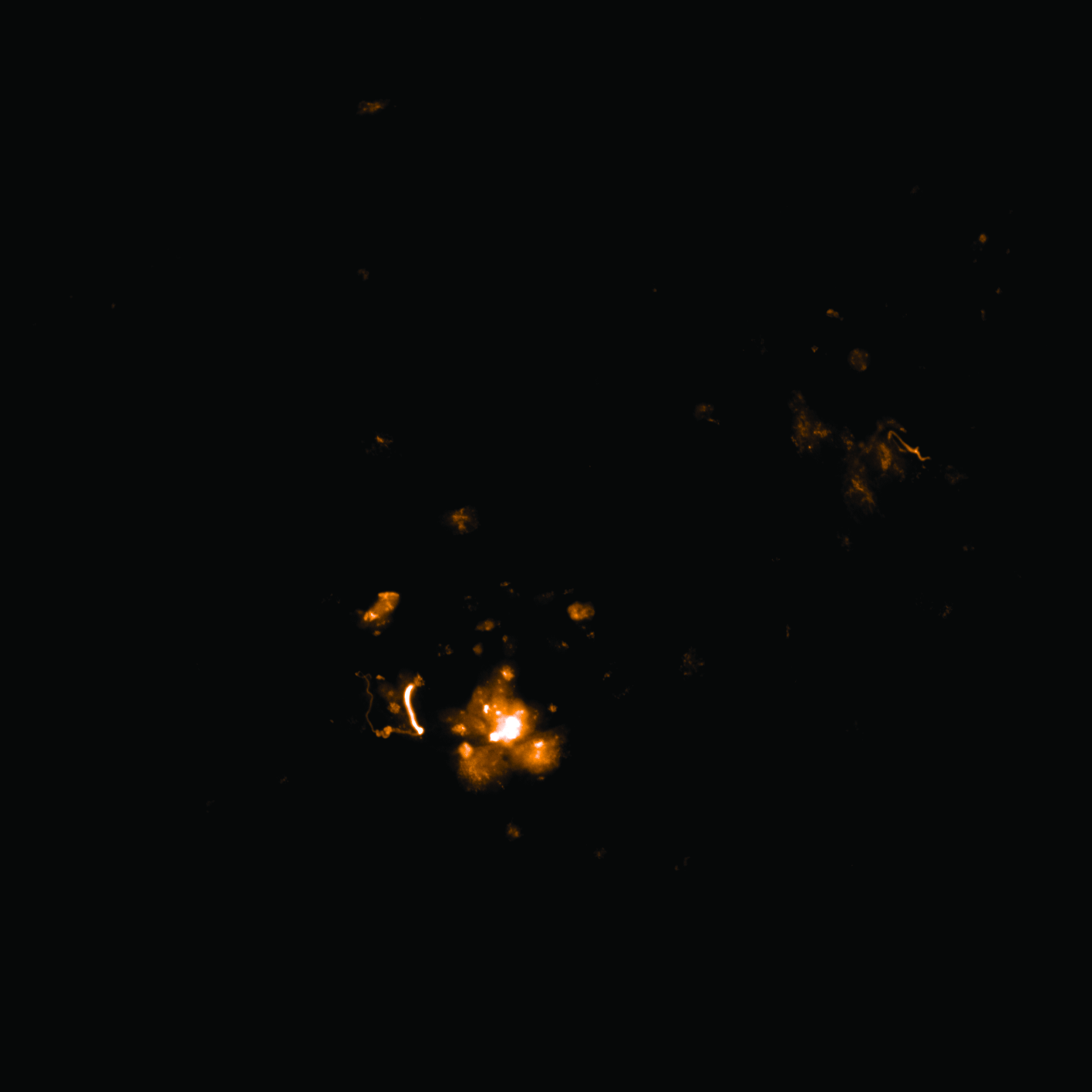

Supplement: Supplementary file 9 — Source data [file 41467_2022_28080_MOESM9_ESM.zip › Source Data/Supp. Figure 19f/Supp. Figure 19f - F8+RecF8 EC F8.tif]

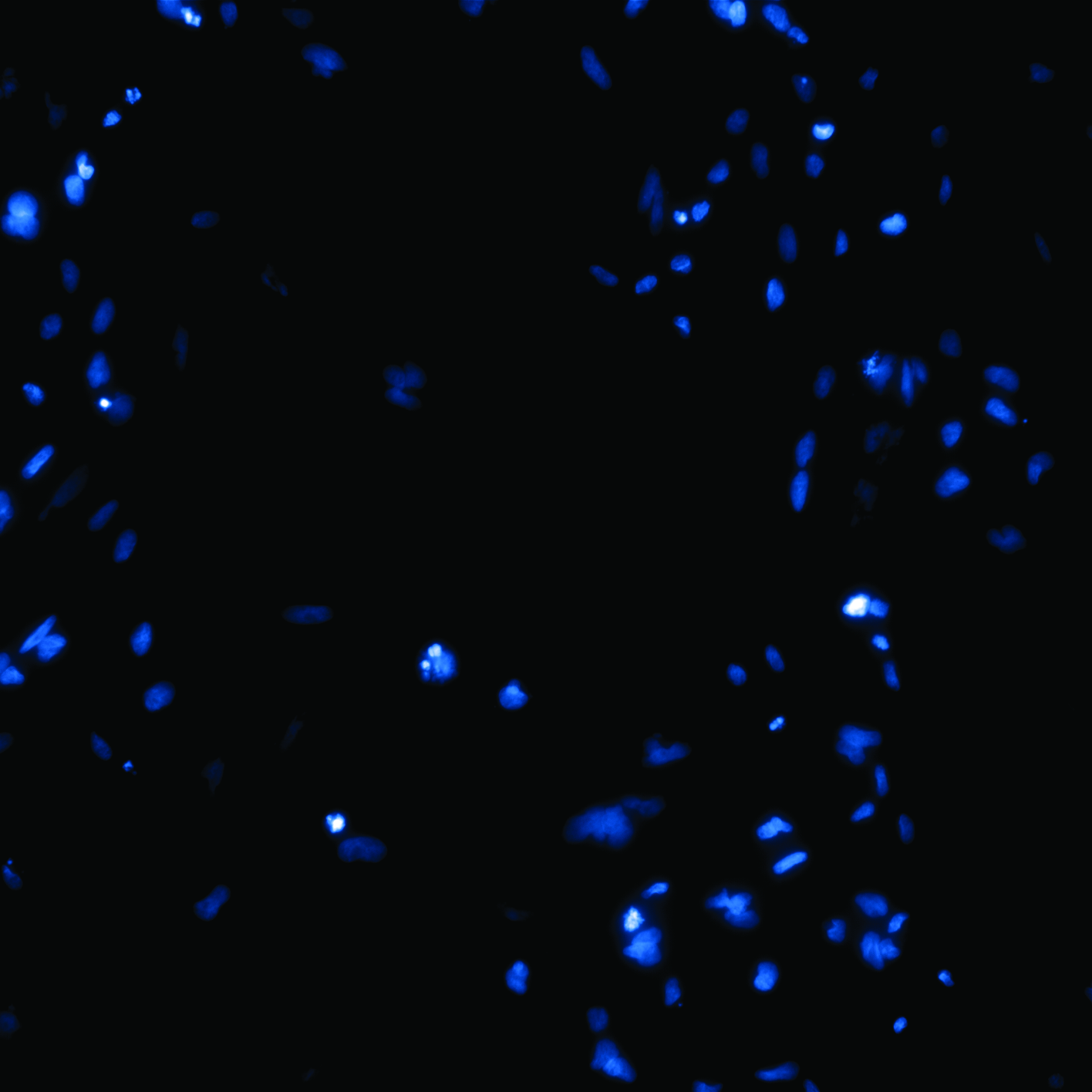

Supplement: Supplementary file 9 — Source data [file 41467_2022_28080_MOESM9_ESM.zip › Source Data/Supp. Figure 19f/Supp. Figure 19f - WT EC 2nd only DAPI.tif]

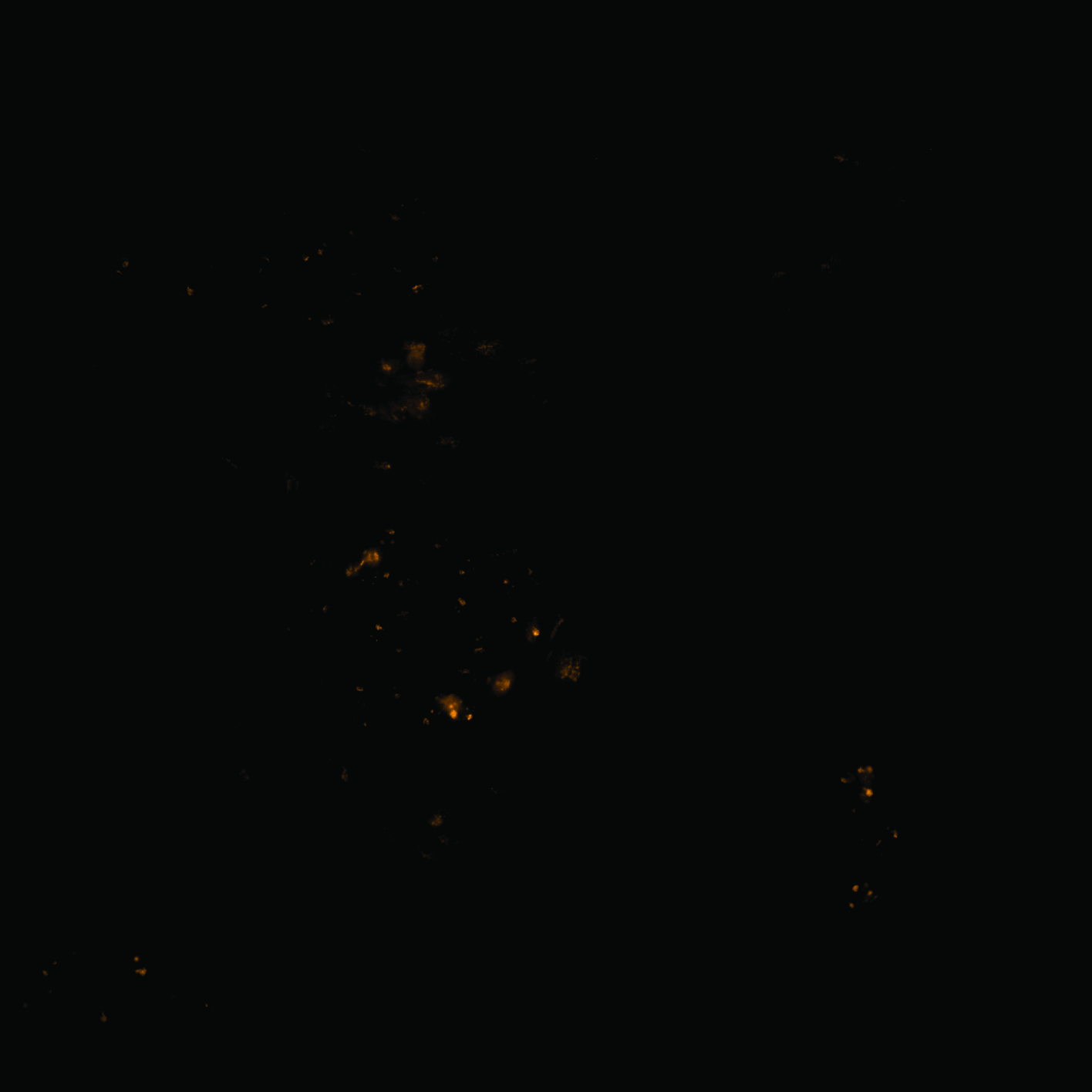

Supplement: Supplementary file 9 — Source data [file 41467_2022_28080_MOESM9_ESM.zip › Source Data/Supp. Figure 19f/Supp. Figure 19f - F8 EC F8.tif]

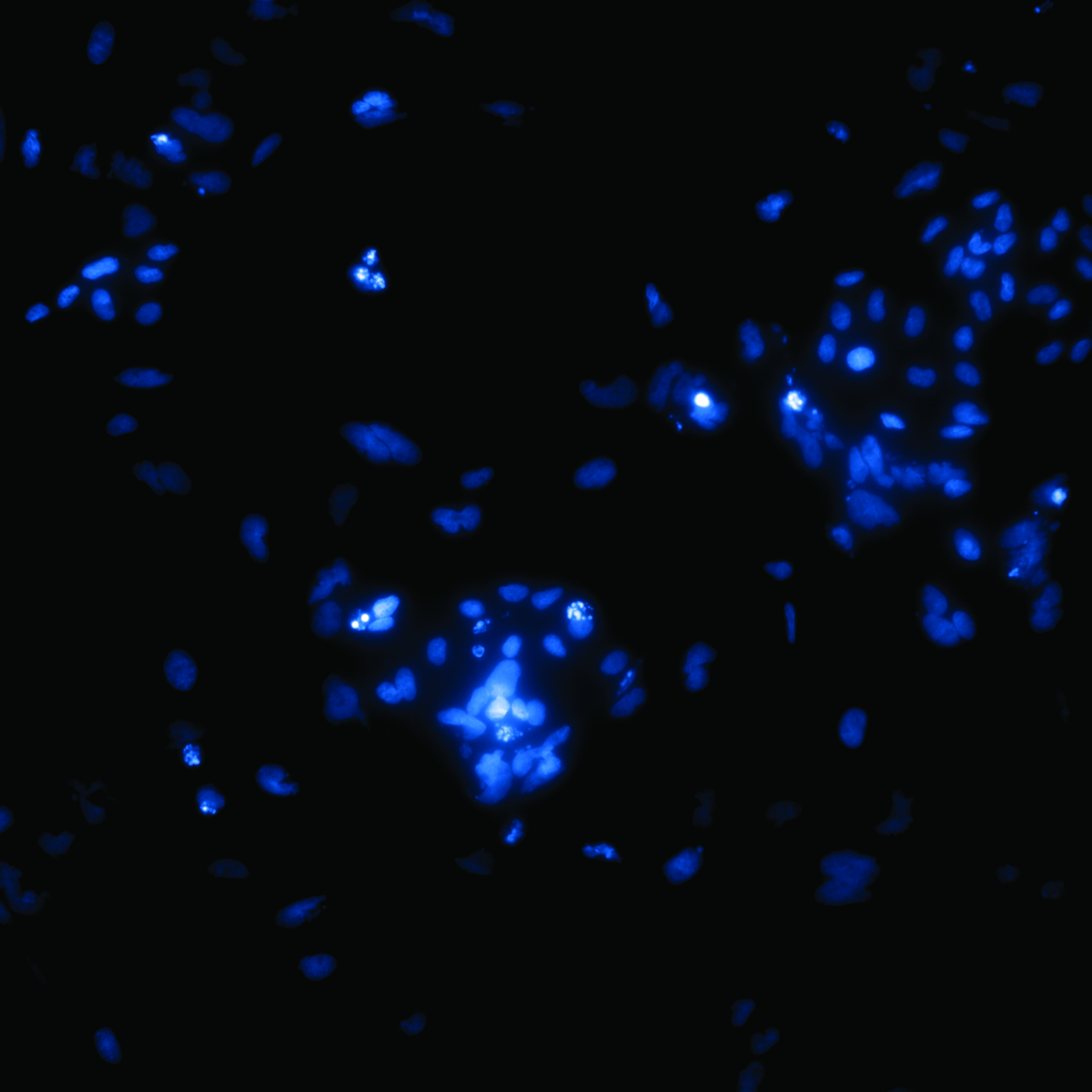

Supplement: Supplementary file 9 — Source data [file 41467_2022_28080_MOESM9_ESM.zip › Source Data/Supp. Figure 19f/Supp. Figure 19f - F8+RecF8 EC DAPI.tif]

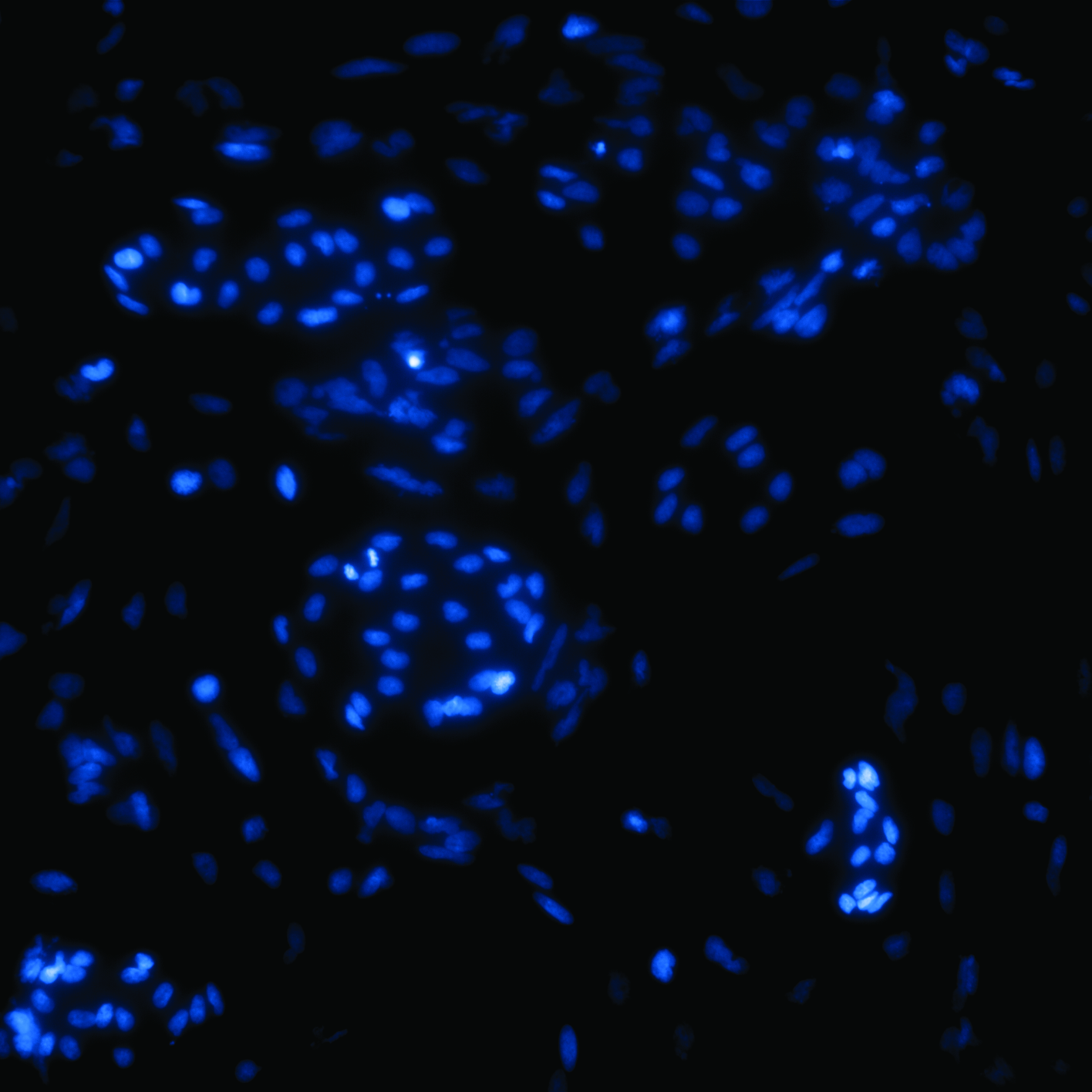

Supplement: Supplementary file 9 — Source data [file 41467_2022_28080_MOESM9_ESM.zip › Source Data/Supp. Figure 19f/Supp. Figure 19f - F8 EC DAPI.tif]

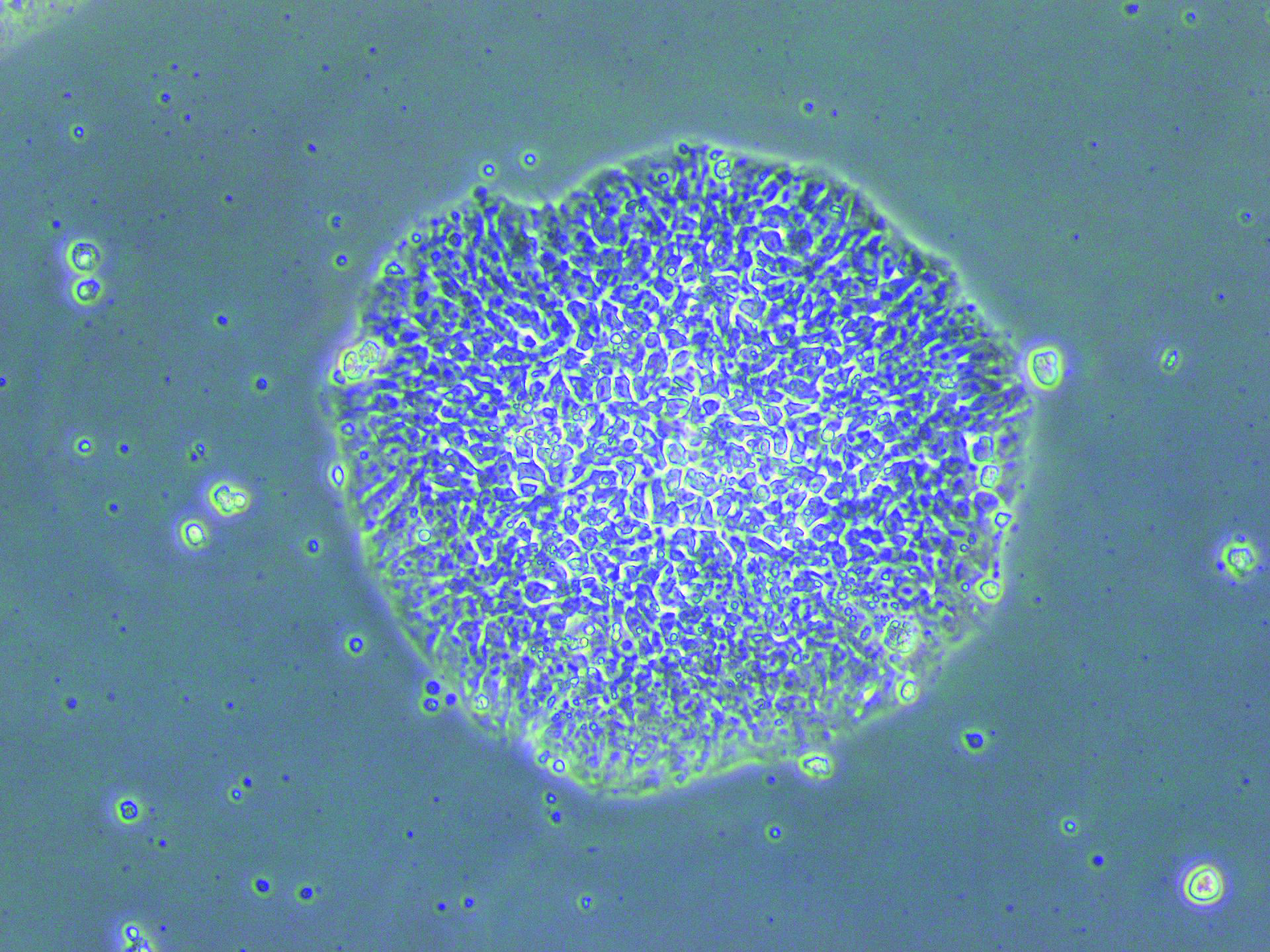

Supplement: Supplementary file 9 — Source data [file 41467_2022_28080_MOESM9_ESM.zip › Source Data/Supp. Figure 18b/Supp. Figure 18b - F8 iPSCs_10x.tif]

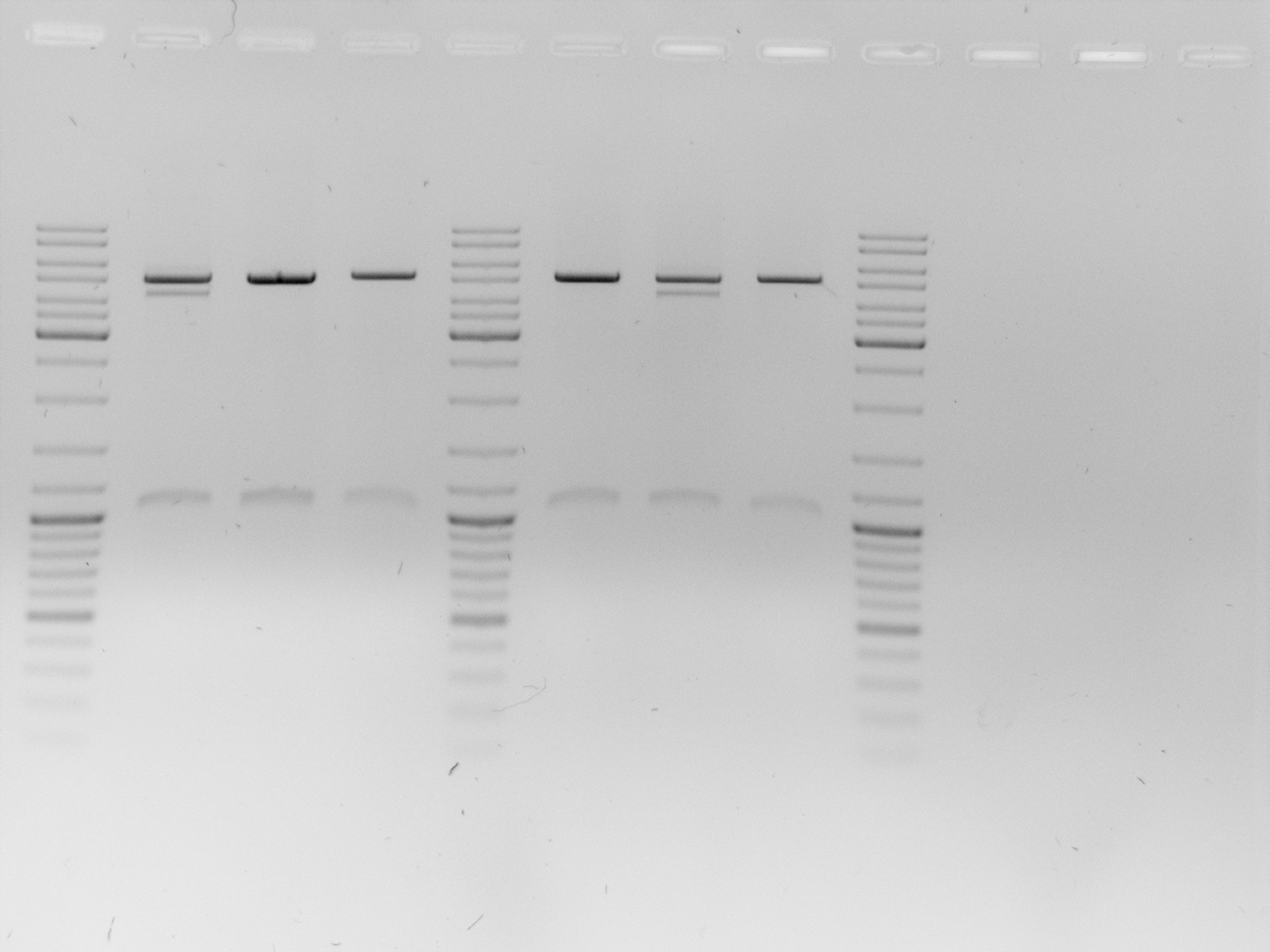

Supplement: Supplementary file 9 — Source data [file 41467_2022_28080_MOESM9_ESM.zip › Source Data/Supp. Figure 4/Supp. Figure 4 - cross-reactivity of the final recombinase libraries.Tif]

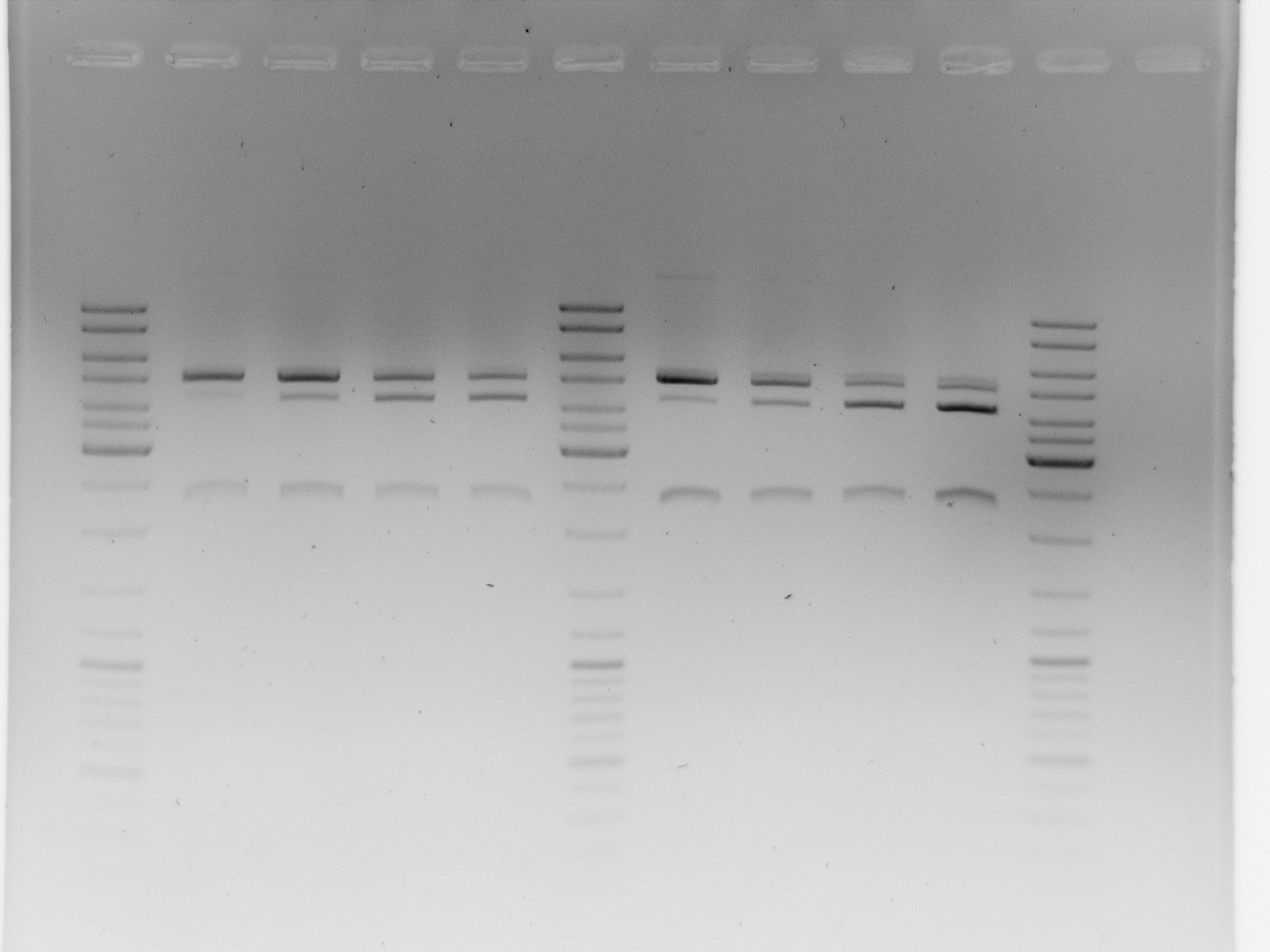

Supplement: Supplementary file 9 — Source data [file 41467_2022_28080_MOESM9_ESM.zip › Source Data/Supp. Figure 12c/Supp. Figure 12c - S2-link lib2-S1 START-END on loxF8 at ara 1,10,100,200. S2-link lib2-S1 END on loxF8 at ara 1,10,100,200 wh.tif]

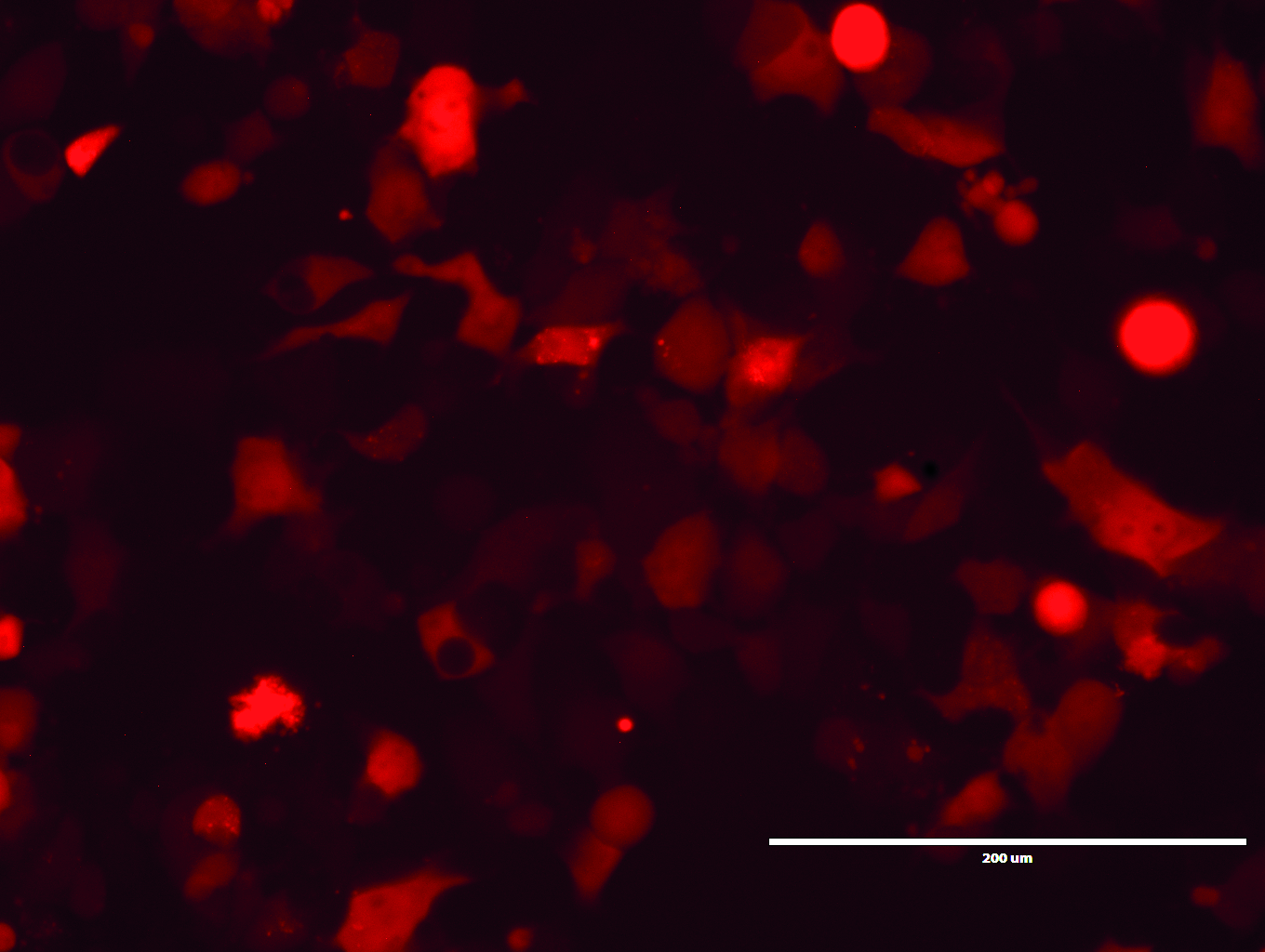

Supplement: Supplementary file 9 — Source data [file 41467_2022_28080_MOESM9_ESM.zip › Source Data/Supp. Figure 14a/Supp. Figure 14a - L8-loxF8rep-mCh.tif]

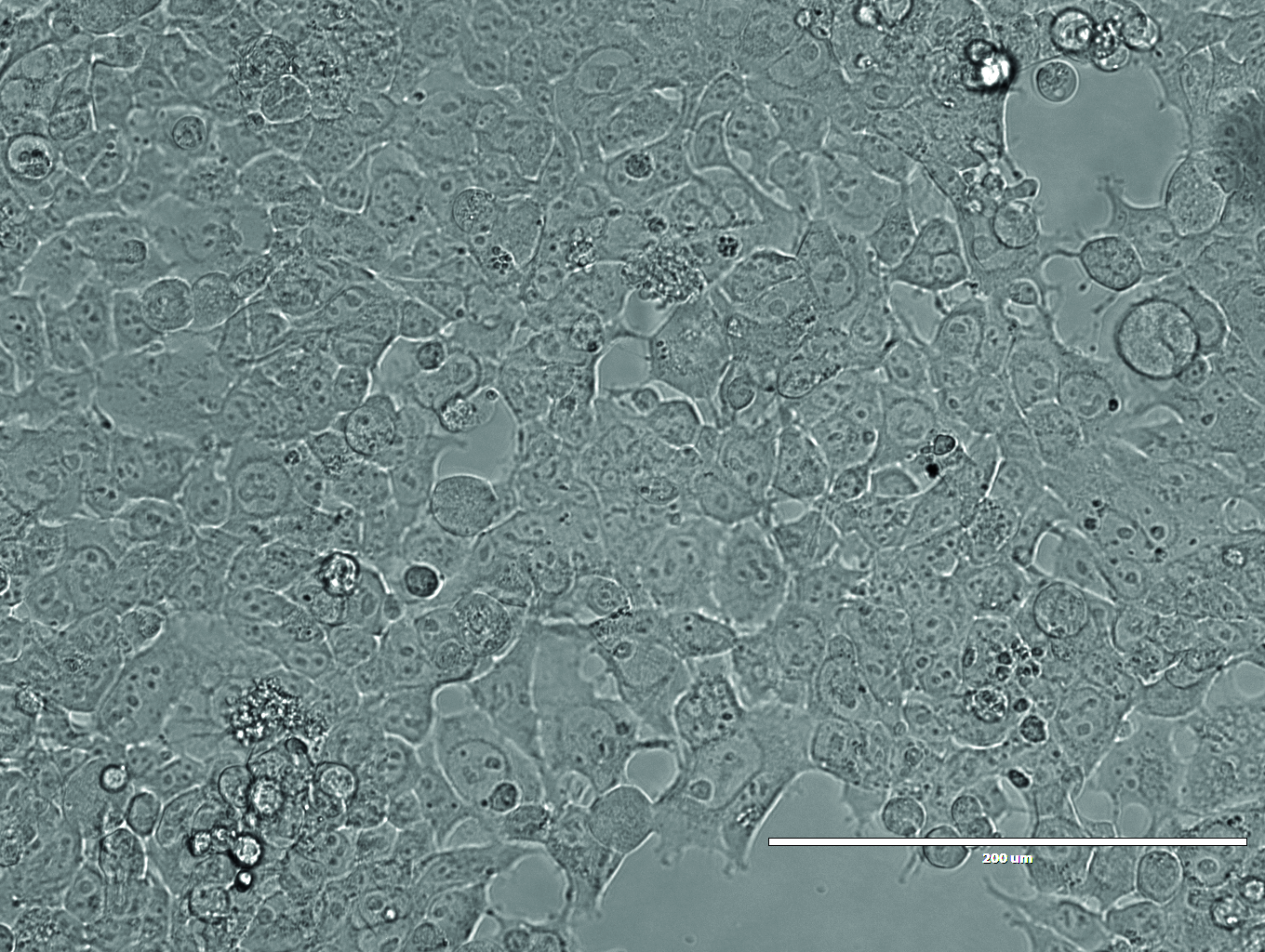

Supplement: Supplementary file 9 — Source data [file 41467_2022_28080_MOESM9_ESM.zip › Source Data/Supp. Figure 14a/Supp. Figure 14a - L8-loxF8rep-BF.tif]

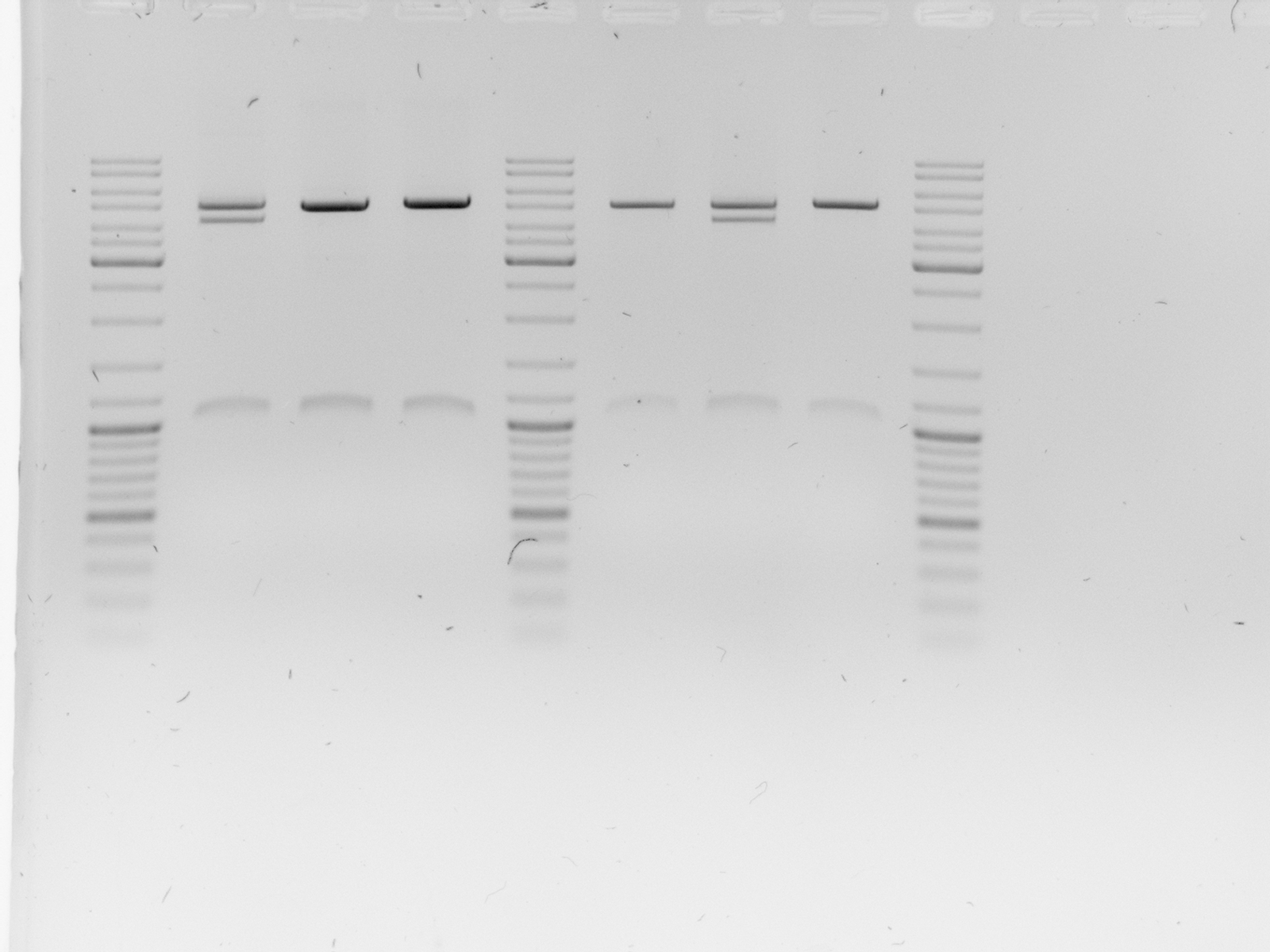

Supplement: Supplementary file 9 — Source data [file 41467_2022_28080_MOESM9_ESM.zip › Source Data/Supp. Figure 5/Supp. Figure 5 - cross-reactivity of the final recombinase libraries post tox.Tif]

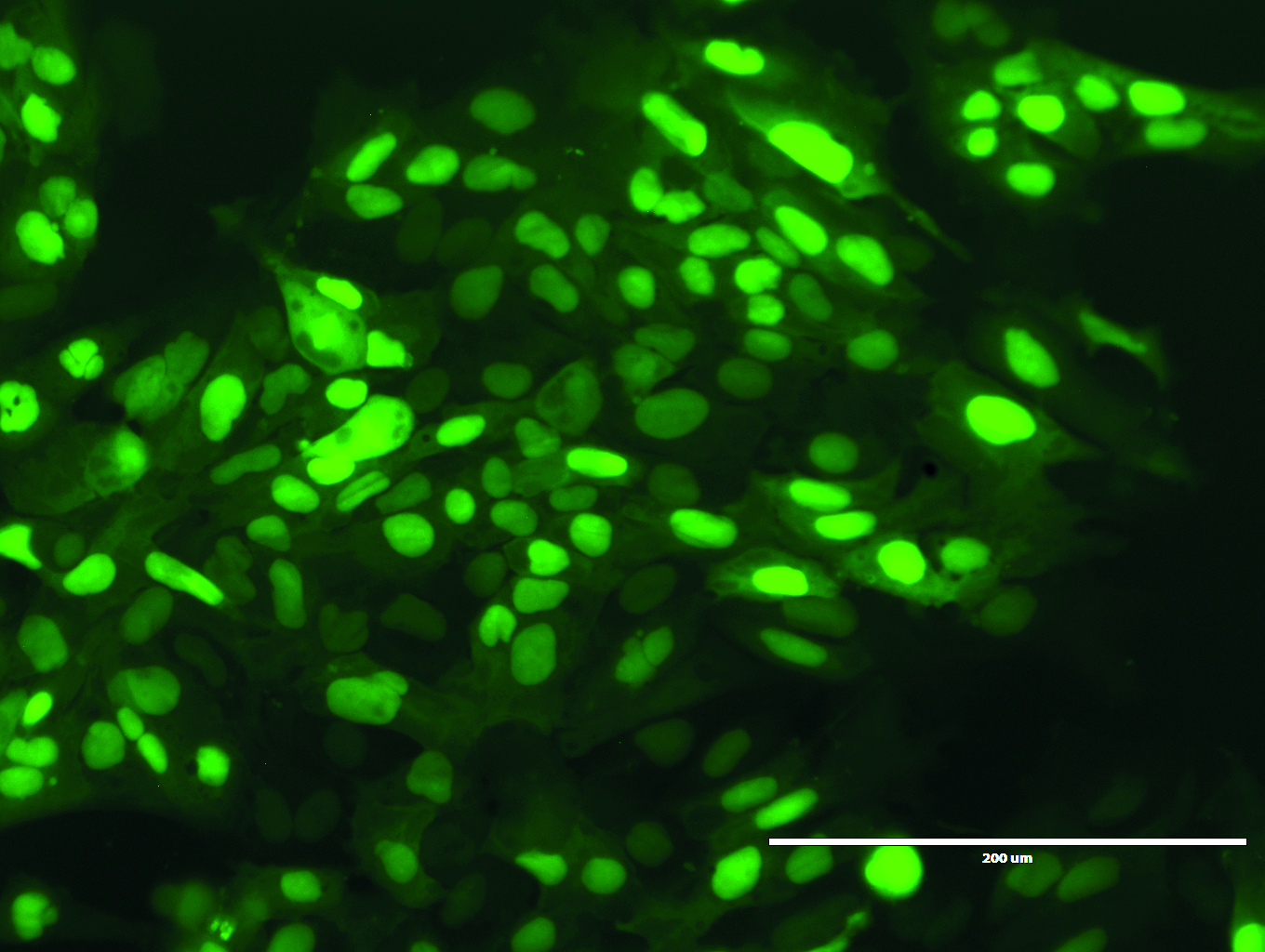

Supplement: Supplementary file 9 — Source data [file 41467_2022_28080_MOESM9_ESM.zip › Source Data/Supp. Figure 18d/Supp. Figure 18d - F8-iPSCs-GFPnls-20x.tif]

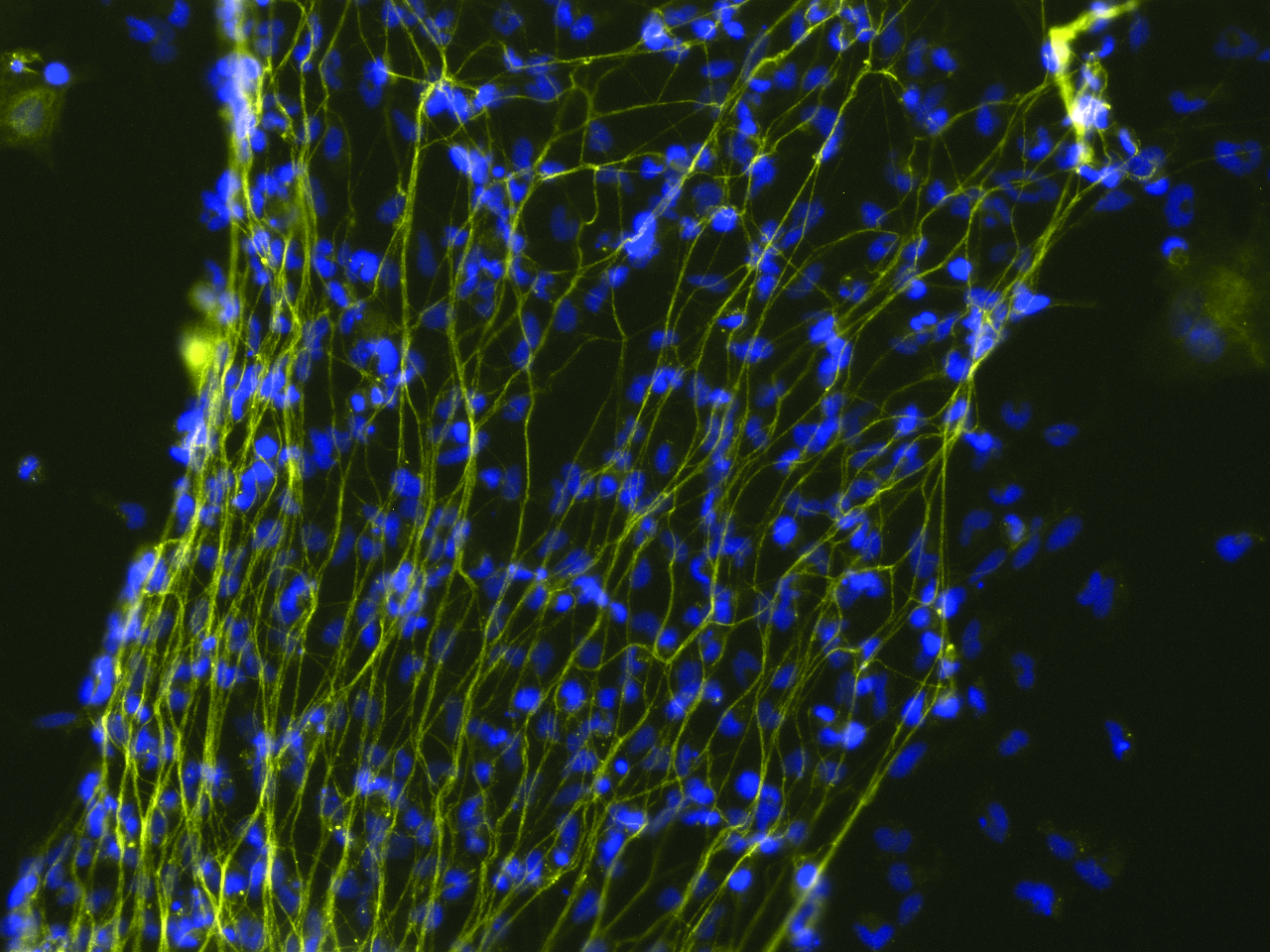

Supplement: Supplementary file 9 — Source data [file 41467_2022_28080_MOESM9_ESM.zip › Source Data/Supp. Figure 18c/Supp. Figure 18c - Ect merge.tif]

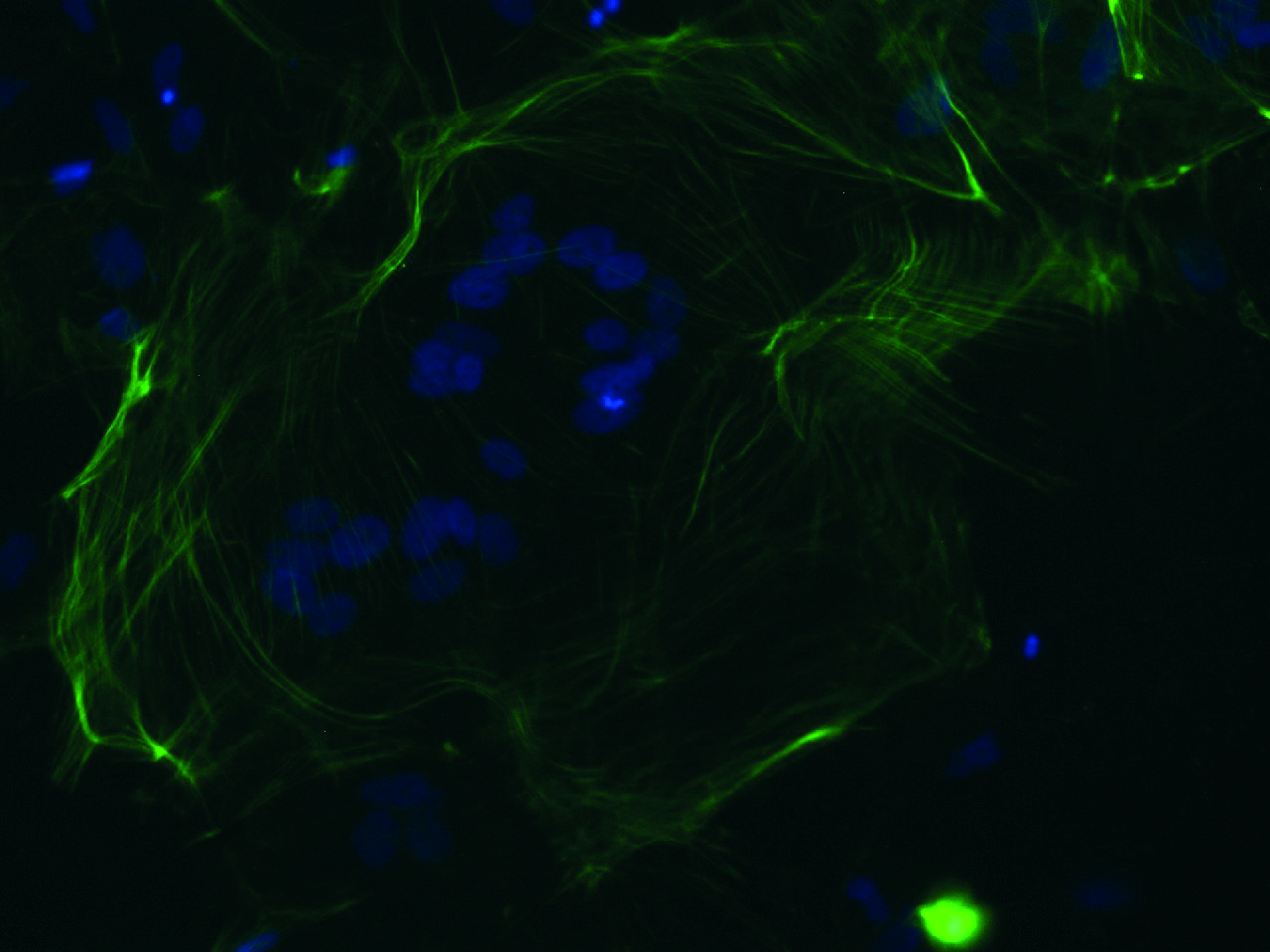

Supplement: Supplementary file 9 — Source data [file 41467_2022_28080_MOESM9_ESM.zip › Source Data/Supp. Figure 18c/Supp. Figure 18c - Mes merge.tif]

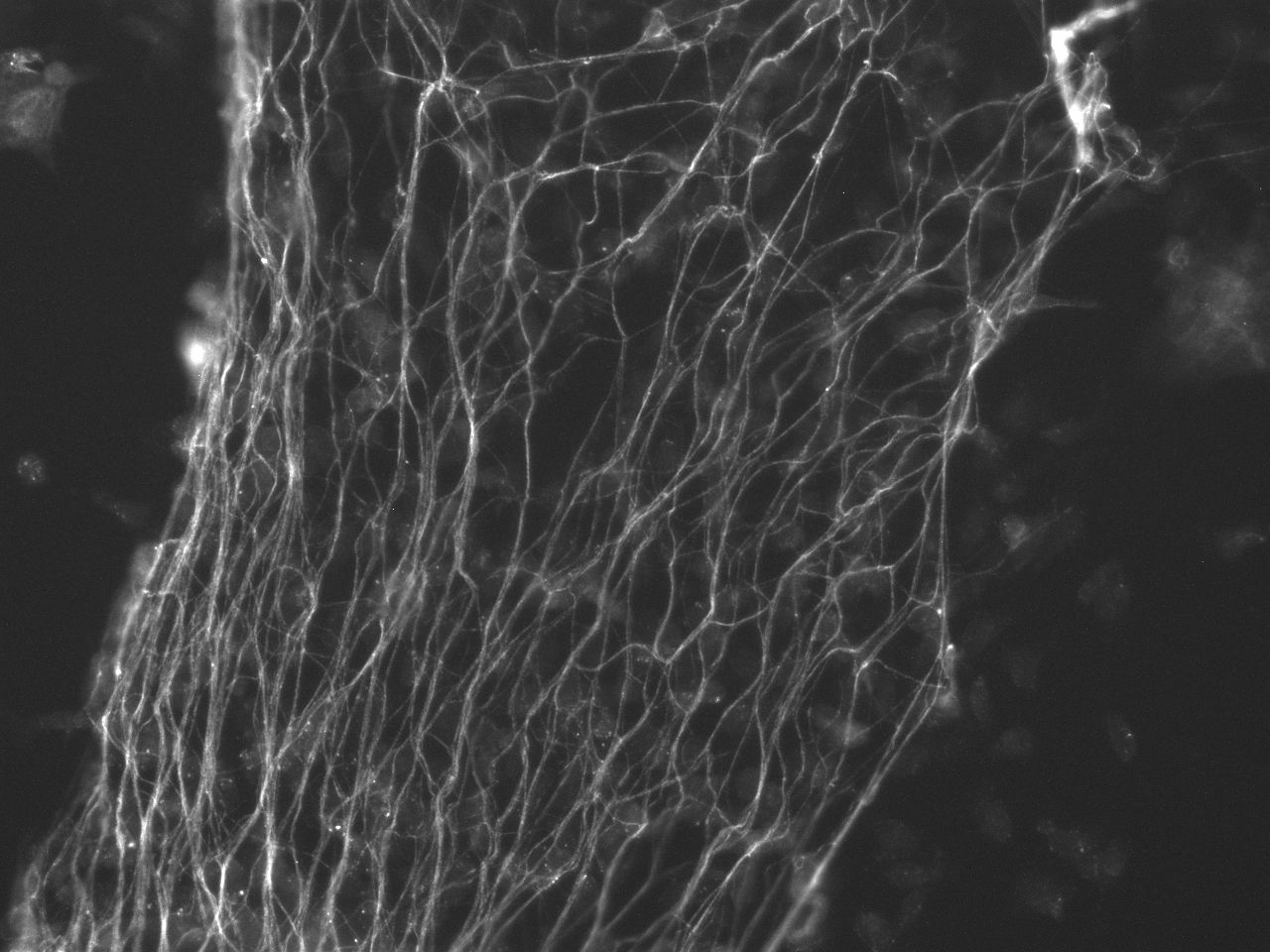

Supplement: Supplementary file 9 — Source data [file 41467_2022_28080_MOESM9_ESM.zip › Source Data/Supp. Figure 18c/Supp. Figure 18c - Ect TUJ-20X.tif]

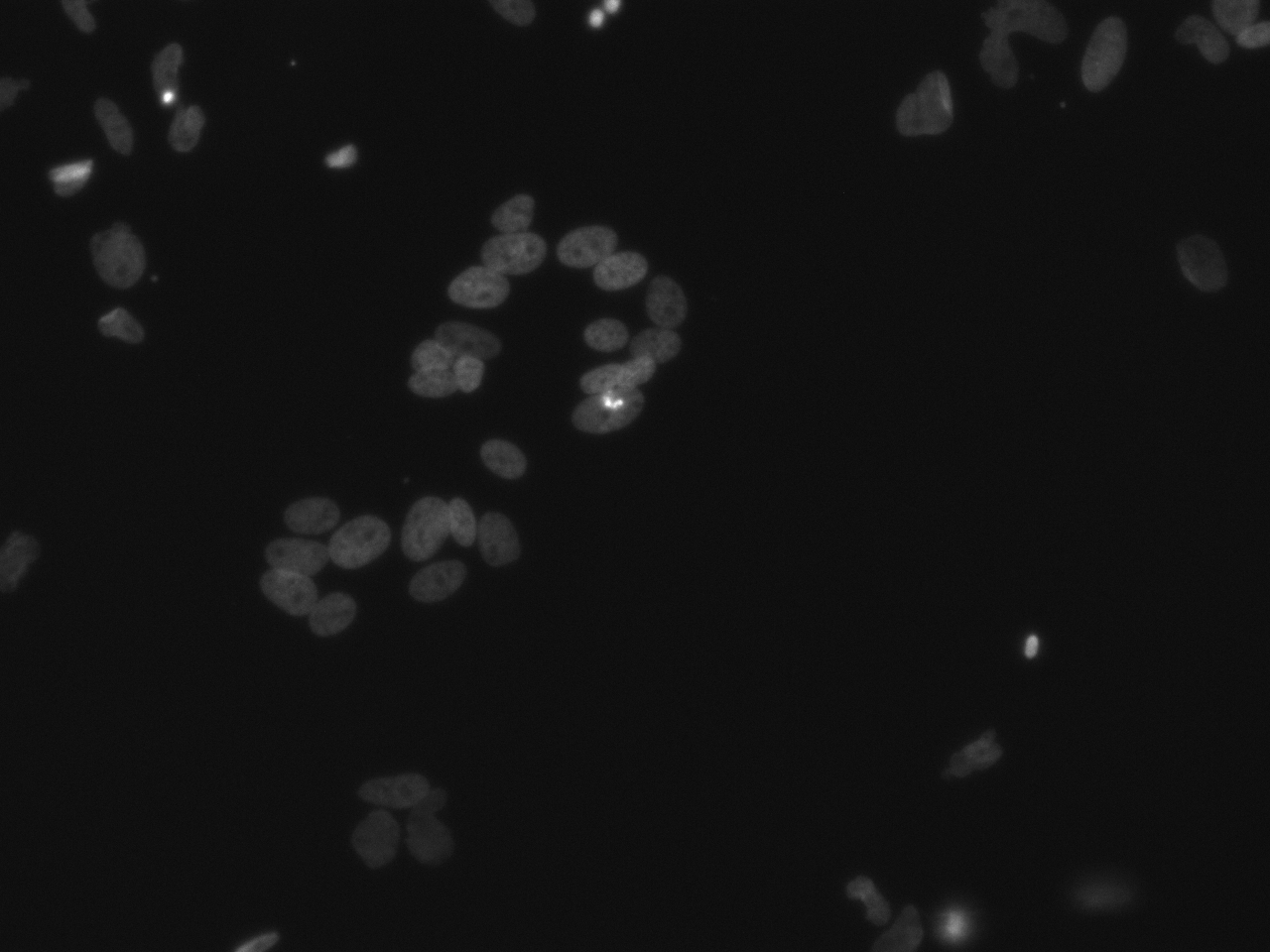

Supplement: Supplementary file 9 — Source data [file 41467_2022_28080_MOESM9_ESM.zip › Source Data/Supp. Figure 18c/Supp. Figure 18c - Mes DAPI.tif]

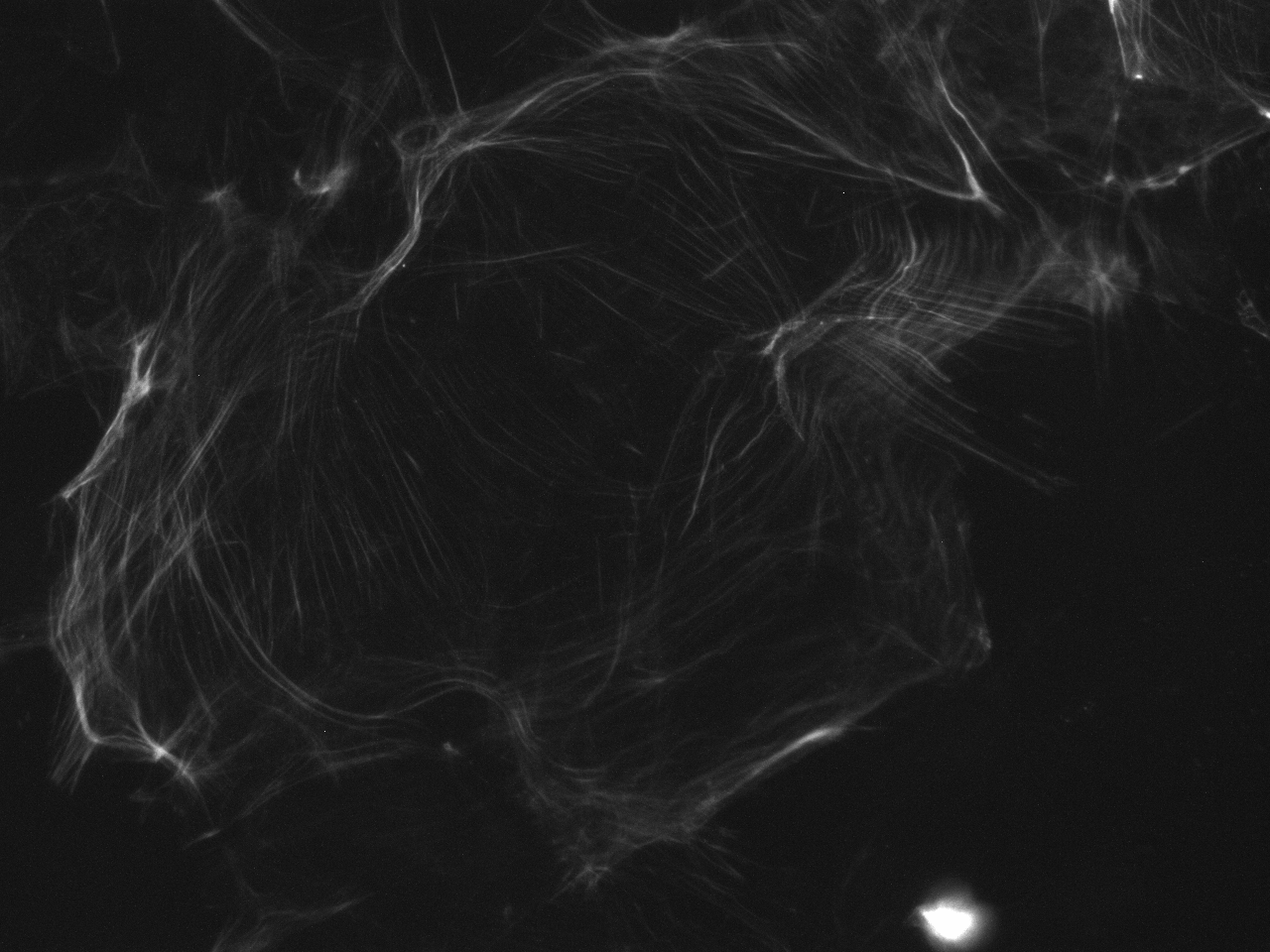

Supplement: Supplementary file 9 — Source data [file 41467_2022_28080_MOESM9_ESM.zip › Source Data/Supp. Figure 18c/Supp. Figure 18c - Mes SMA .tif]

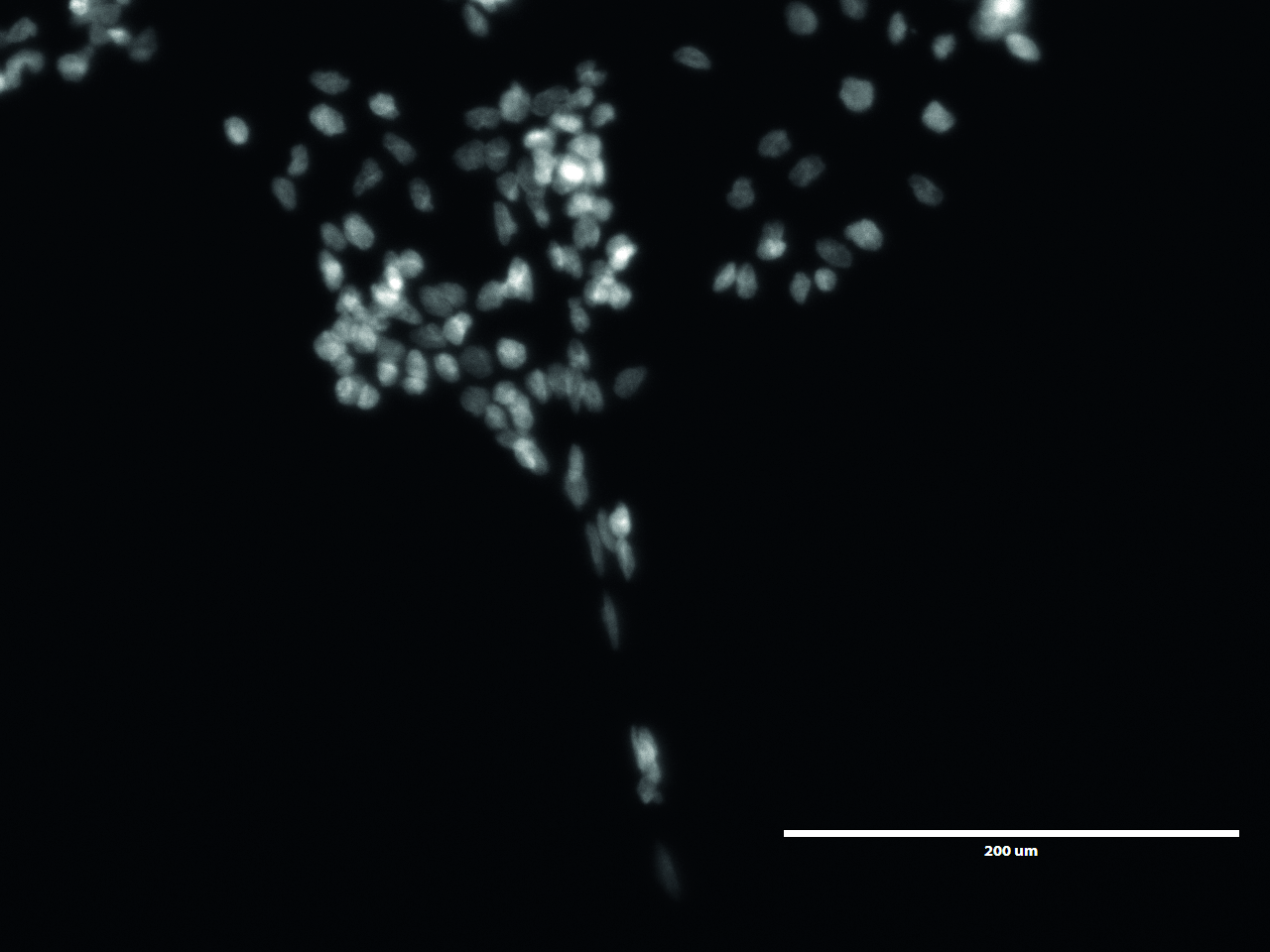

Supplement: Supplementary file 9 — Source data [file 41467_2022_28080_MOESM9_ESM.zip › Source Data/Supp. Figure 18c/Supp. Figure 18c - End DAPI.tif]

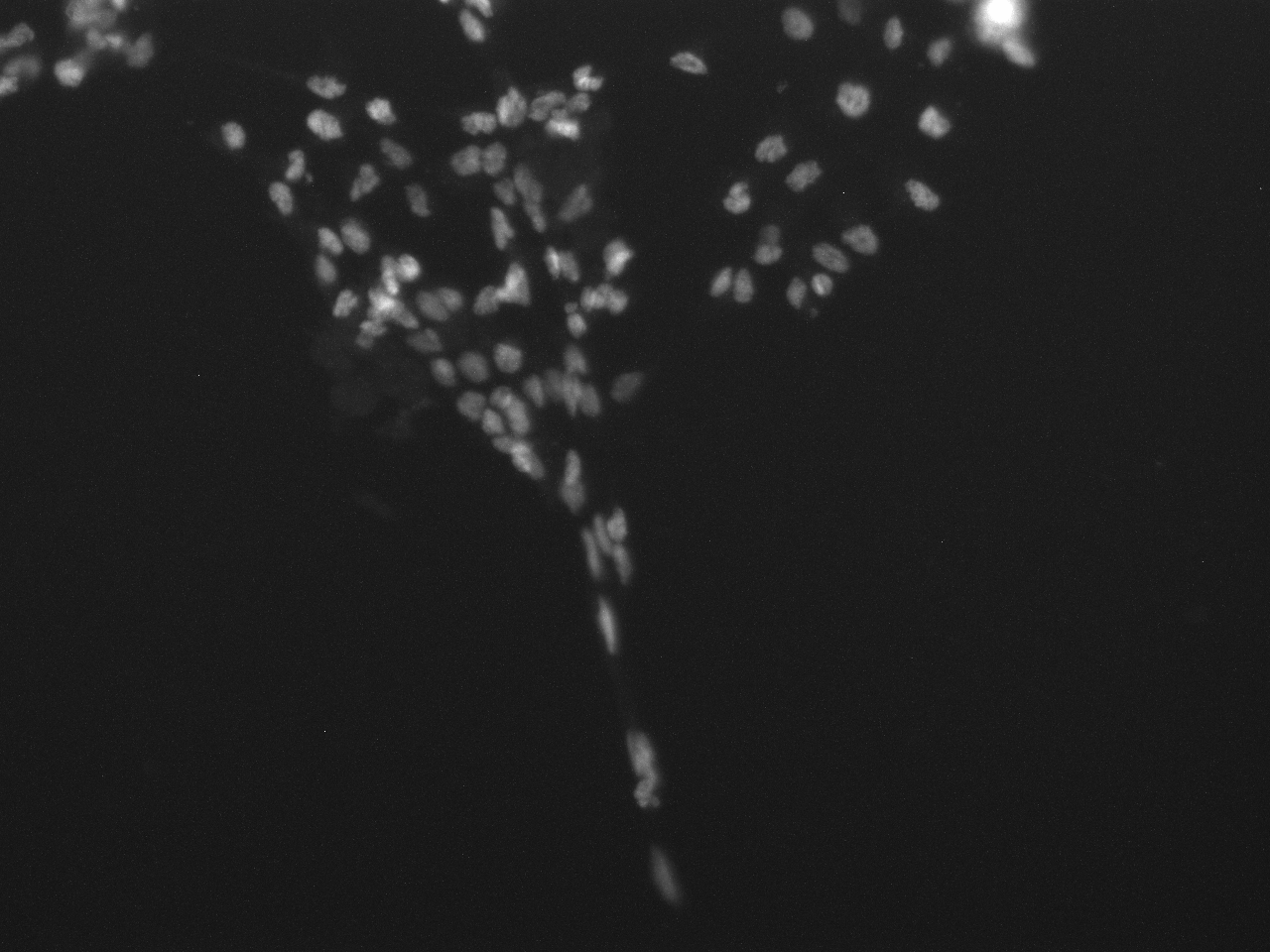

Supplement: Supplementary file 9 — Source data [file 41467_2022_28080_MOESM9_ESM.zip › Source Data/Supp. Figure 18c/Supp. Figure 18c - End SOX17 20X.tif]

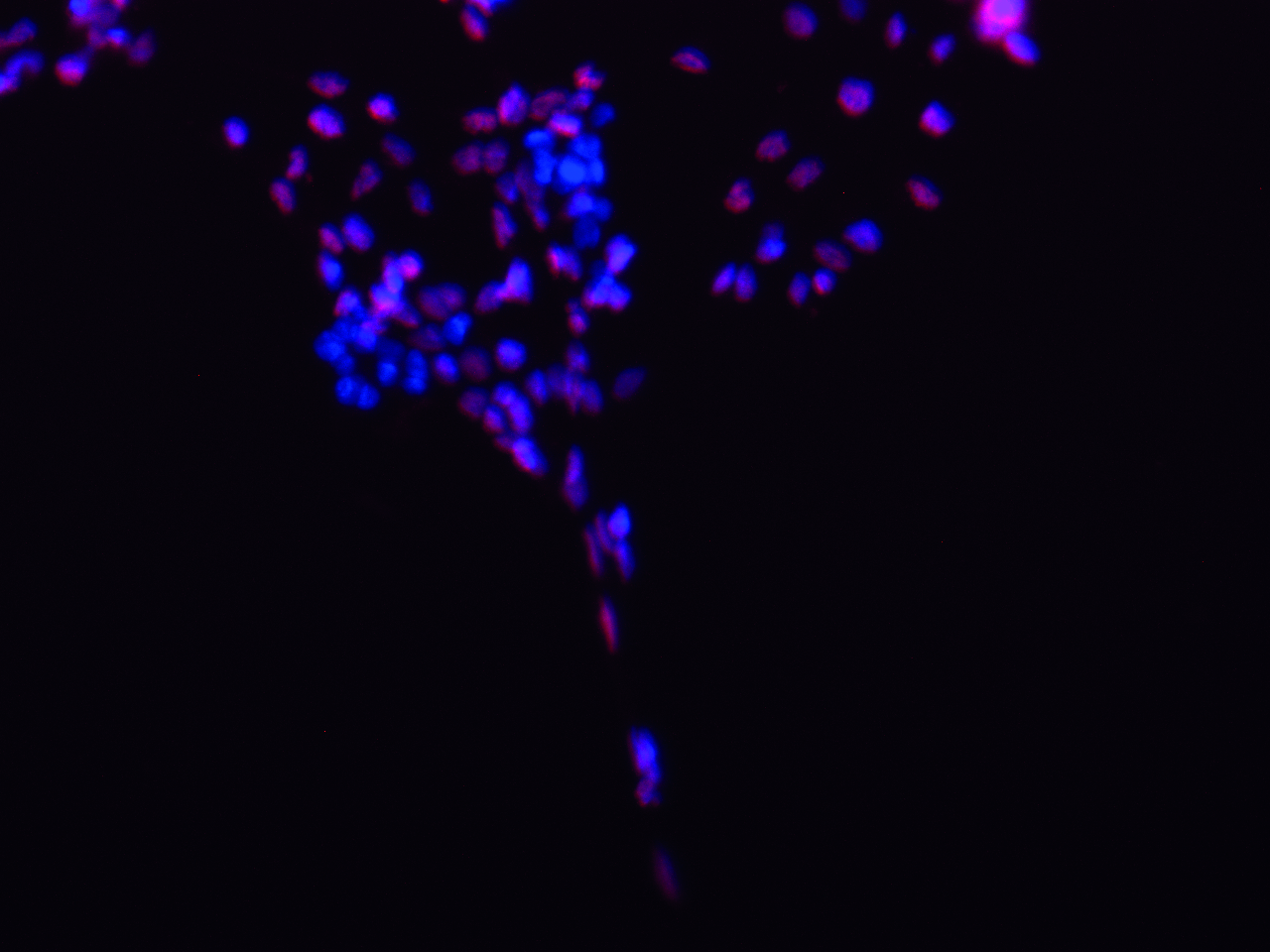

Supplement: Supplementary file 9 — Source data [file 41467_2022_28080_MOESM9_ESM.zip › Source Data/Supp. Figure 18c/Supp. Figure 18c - End merge.tif]

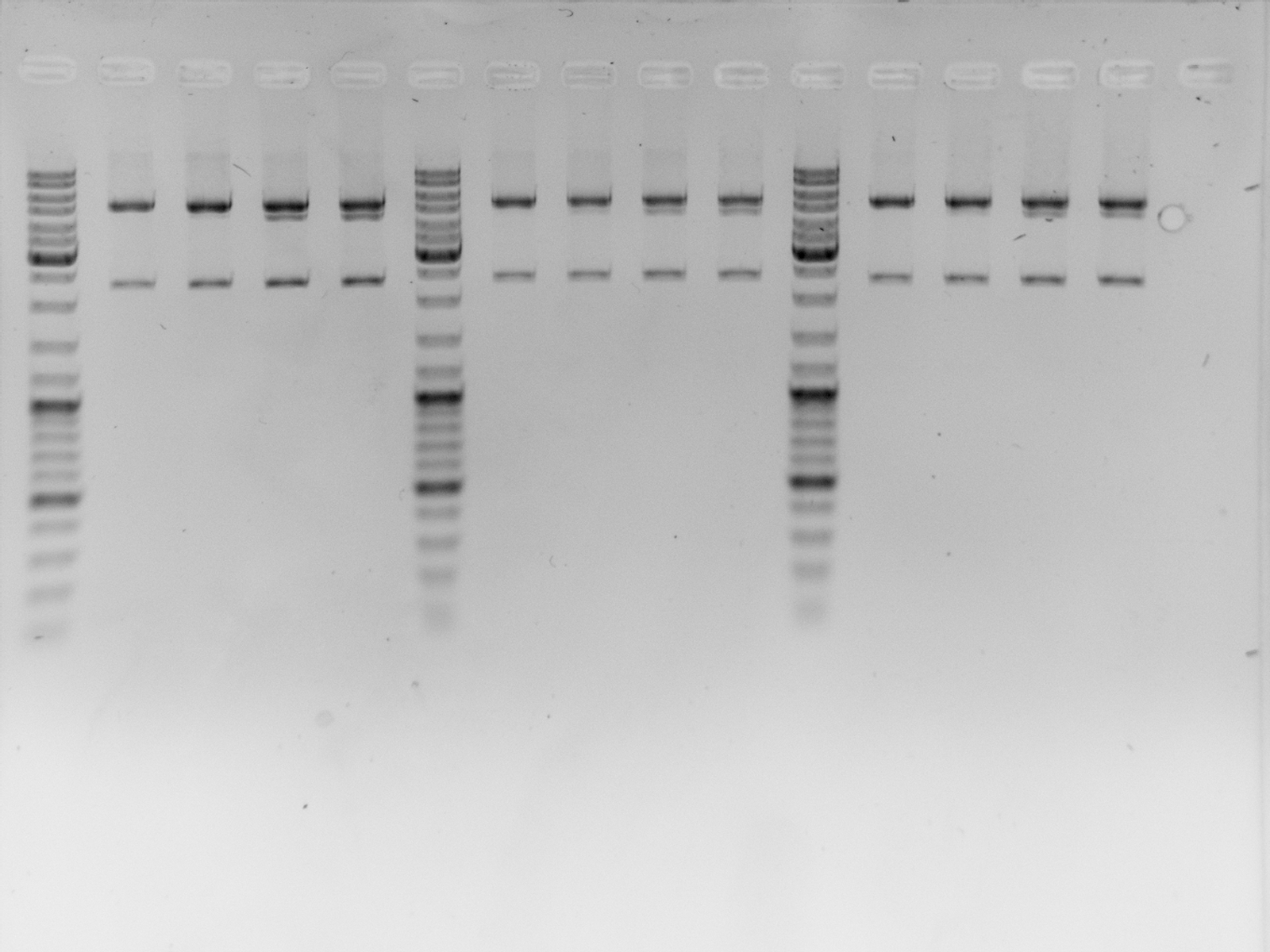

Supplement: Supplementary file 9 — Source data [file 41467_2022_28080_MOESM9_ESM.zip › Source Data/Supp. Figure 6c/Supp. Figure 6c - D4, D9, D10 - 0, 1,10, 100 ara on loxF8.Tif]

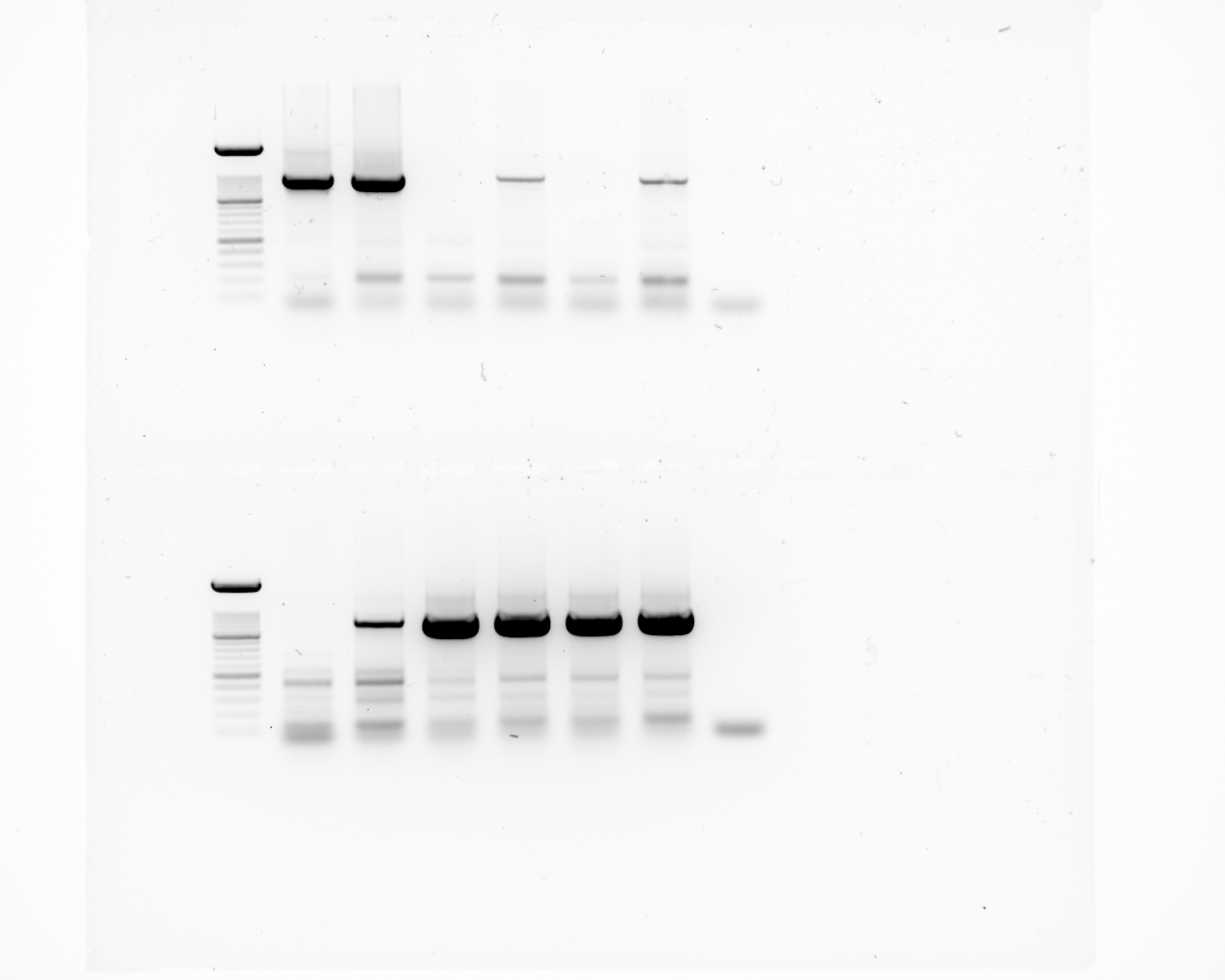

Supplement: Supplementary file 9 — Source data [file 41467_2022_28080_MOESM9_ESM.zip › Source Data/Figure 5c/Figure 5b - inversion orientation lower part of the gel.tif]

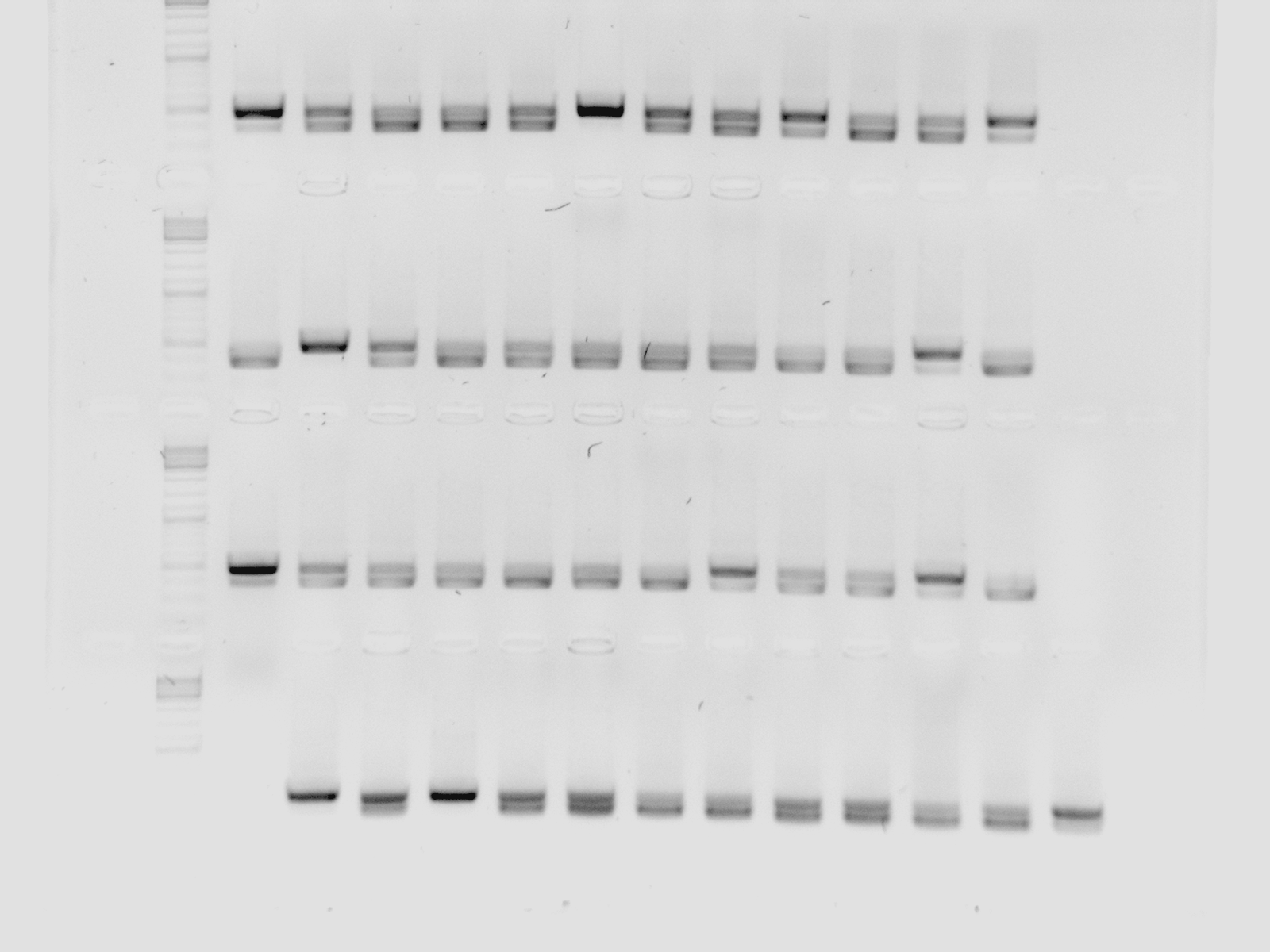

Supplement: Supplementary file 9 — Source data [file 41467_2022_28080_MOESM9_ESM.zip › Source Data/Supp. Figure 6b/Supp. Figure 6b - F8 dimer 49-96 clone.Tif]

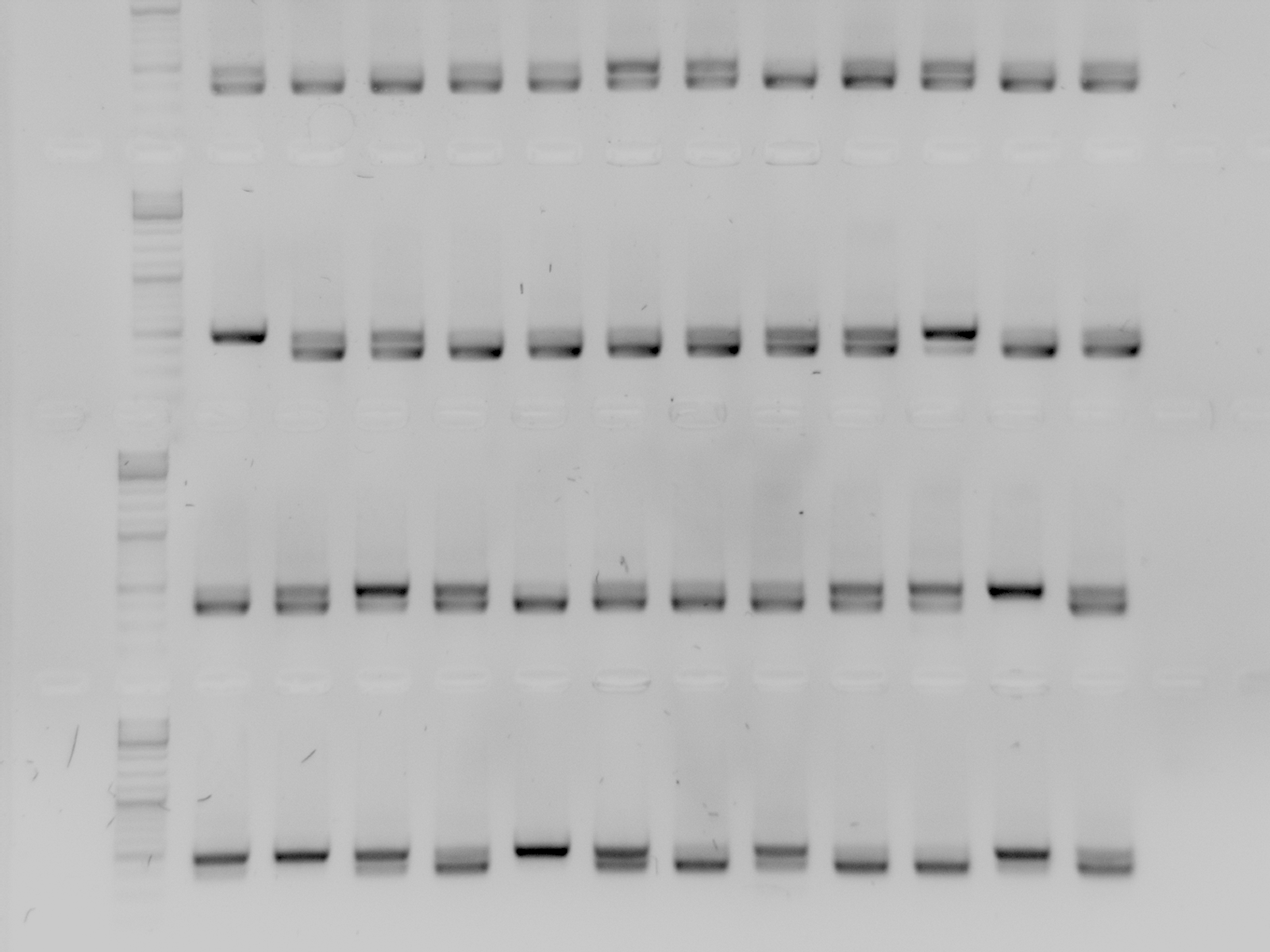

Supplement: Supplementary file 9 — Source data [file 41467_2022_28080_MOESM9_ESM.zip › Source Data/Supp. Figure 6b/Supp. Figure 6b - F8 dimer 1-48 clone.Tif]

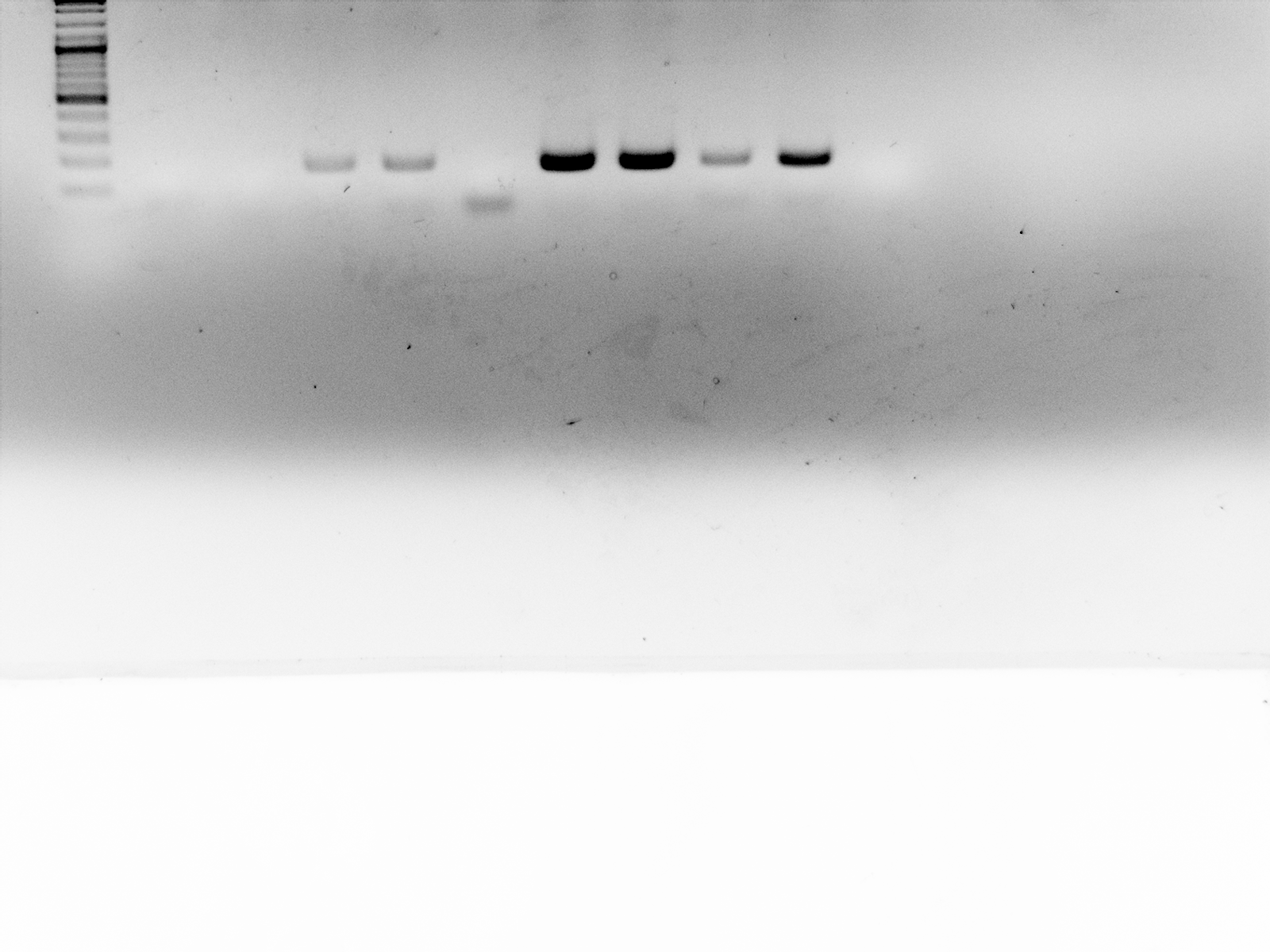

Supplement: Supplementary file 9 — Source data [file 41467_2022_28080_MOESM9_ESM.zip › Source Data/Figure 6d/Figure 6d - PCR on cDNA.Tif]
